# Silk Gland Gene Expression during Larval-Pupal Transition in the Cotton Leaf Roller *Sylepta derogata* (Lepidoptera: Pyralidae)

**DOI:** 10.1371/journal.pone.0136868

**Published:** 2015-09-09

**Authors:** Honghua Su, Yuming Cheng, Zhongyang Wang, Zhong Li, David Stanley, Yizhong Yang

**Affiliations:** 1 School of Horticulture and Plant Protection of Yangzhou University, Yangzhou, Jiangsu, China, 225009; 2 Yangzhou Termite Control Center, Yangzhou, Jiangsu, China, 225001; 3 USDA – Agricultural Research Service, Biological Control of Insects Research Laboratory, 1503 S. Providence Road, Columbia, MO, United States of America, 65203; Institute of Plant Physiology and Ecology, CHINA

## Abstract

The cotton leaf roller, *Sylepta derogata*, is a silk-producing insect pest. While young larvae feed on the underside of leaves, the older ones roll cotton leaves and feed on the leaf edges, which defoliates cotton plants. The larvae produce silk to stabilize the rolled leaf and to balloon from used to new leaves. Despite the significance of silk in the biology of pest insect species, there is virtually no information on the genes involved in their silk production. This is a substantial knowledge gap because some of these genes may be valuable targets for developing molecular pest management technologies. We addressed the gap by posing the hypothesis that silk gland gene expression changes during the transition from larvae to pupae. We tested our hypothesis using RNA-seq to investigate changes in silk gland gene expression at three developmental stages, 5^th^ instar larvae (silk producing; 15,445,926 clean reads), prepupae (reduced silk producing; 13,758,154) and pupae (beyond silk producing; 16,787,792). We recorded 60,298 unigenes and mapped 50,158 (larvae), 48,415 (prepupae) and 46,623 (pupae) of them to the NCBI database. Most differentially expressed genes in the 5^th^ instar larvae/prepupae libraries were relevant to nucleotide synthesis and maintenance of silk gland function. We identified down-regulated transcriptional factors and several genes involved in silk formation in the three libraries and verified the expression pattern of eight genes by qPCR. The developmental- and tissue-specific expression patterns of the fibroin light chain gene showed it was highly expressed during the larval silk-producing stage. We recorded highest expression of this gene in the larval silk gland, compared to other tissues, including midgut, hindgut, epidermis, Malpighian tubes, hemolymph and fat body. These data are a genetic resource to guide selection of key genes that may be targeted for *in planta* and other gene-silencing technologies for sustainable cotton agriculture.

## Introduction

Insects representing at least 20 orders produce silk thread. Some produce silk only in the larval or adult stages and others in both [1]. In a narrow sense, the term ‘silk-producing insects’ refers to domesticated and wild species used in commercial silk industries, however, the silks of many insects, particularly pest species, are not commercially useful. Some silk-producing insects inflict tremendous damage on crops and forests. For example, neonates of the lackey moth, *Malacosoma neustria testacea* Motsch, a forest and fruit tree pest, produce silk, form a larval web, and then feed on new buds and leaves. All the leaves can be destroyed during population outbreaks [2]. The rice leaf folder, *Cnaphalocrocis medinalis* Guenee (Lepidoptera: Pyralidae) folds rice leaves vertically with silk, creating a sort of tent in which larvae feed on the rice epicuticle and leaf tissue. The resulting damage leads to substantial rice production losses [3]. The cotton leaf roller (CLR), *Sylepta derogata* Fabricius, rolls cotton leaves into conical structures and feeds on the edges of the rolled leaf, resulting in serious defoliation. The larvae use silk to stabilize their leaf cones and to balloon to other leaves. High pest populations significantly decrease cotton production [4].

Although silk plays fundamental roles in the biology of these and other pests, information on their silk glands and mechanisms of silk production remains relatively scarce, particularly compared to the abundant literature on silk production in the domesticated silk worm, *Bombyx mori* [5]. This is a crucial lacuna because silk glands of insects in various orders develop from different cell types and they are associated with different anatomical structures [6]. More to the point, due to their actions in the biology of pest species, genes involved in silk production are potential targets for crop- and pest-specific molecular technologies designed to manage pests by silencing specific genes. We addressed the lacuna by posing the hypothesis that silk gland gene expression changes during the developmental transition from larvae to pupae. Here, we report on the outcomes of our transcriptomic analyses designed to test our hypothesis.

## Materials and Methods

### Insects

CLRs were collected from local velvetleaf, *Abutilon theophrasti* Medic (Malvaceae), a cosmopolitan weed which grew naturally near the field in the Yangzhou University experimental farm located at 32°42′N, 119°4′E. Neither the CLRs nor the velvetleaf are endangered and protected species. Thus, no specific permissions were required for this research). The insects were maintained on fresh velvetleaf leaves *ab lib* at 27±1°C, 70%±7% relative humidity (RH), and 14L:10D in the laboratory. Fifth-instar larvae, prepupae and pupae (day 2) were anesthetized and the silk glands were isolated (20 glands/preparation, two biologically independent preparations) for transcriptome sequencing. The resulting transcriptomes represent phases of the CLR life cycle characterized by high, reduced and no silk production.

### cDNA library construction and Illumina sequencing

Total RNA was extracted with the SV Total RNA Isolation System (Promega, Madison, WI, USA) following the manufacturer’s protocol. Quality and quantity of the RNA was determined on a Nanodrop spectrophotomter (Thermo Fisher, Massachusetts, USA). cDNA library construction and Illumina sequencing were performed at the Beijing Berry Genomics Co. Ltd. (Beijing, China). 20 μg of pooled total RNA was fragmented in a Biorupter Cracker for 2 min following the manufacturer’s protocol (Diagenode, Liège, Belgium). Fragments of 250–300 bp were recovered and purified using the Oligotex mRNA MiniKit (Qiagen, Dusseldorf, Germany). Using these fragments as templates, random hexamer primers (6 bp) were employed to synthesize first-strand cDNA using Superscript ∏ reverse transcriptase (Invitrogen, California, USA). Second-strand cDNA was generated in reactions composed of buffer (10× blue buffer, Enzymatics, Boston, USA), dNTPs (500μM), RNase H (2,000 UN, 1μl in 50 μl reaction), and DNA polymerase I (5 μl in 50 μl reaction). Following end repair and adaptor ligation, short sequences were amplified by PCR and purified with a QIAquick PCR extraction kit (Qiagen, Venolo, the Netherlands), and sequenced on a HiSeq 2000 platform (San Diego, CA, USA). The reaction conditions were: 25°C, 10 min; 42°C, 50 min; hold at 4°C (for first-strand cDNA synthesis); 25°C, 2.5h (for second-strand cDNA synthesis); 20°C, 30 min (for end repair); 37°C, 30 min (for A attachment) and adaptor ligation was conducted at 20°C for 30 min.

The deep-sequencing dataset was deposited in NCBI GenBank Short Read Archive and the accession number SRP058214.

### Assembly and functional annotation

Transcriptome *de novo* assembly was carried out with the short read assembly program, Trinity (version 2012-06-08), which generated two classes of transcripts: clusters (prefix CL) and singletons (prefix U). Transcripts larger than 200 bp were aligned by BLASTX to the Nr (release-2012-10-05) (ftp://ftp.uniprot.org/pub/databases/uniprot/previous_releases/), KEGG(release 63.0)) (http://www.genome.jp/), and COG (release-2009-03-31) (http://www.ncbi.nlm.nih.gov/COG/))(E-value<10^-5^) databases. These databases retrieved proteins with the highest sequence similarity to the given transcripts, along with their protein functional annotations. We then used the Blast2Go program for GO annotation and WEGO software to plot the GO annotation results.

### Analysis of transcript expression differences between developmental stages

The transcript expression abundances were calculated by Reads per Kilobase of exon model per Million mapped reads (RPKM), which eliminates the influence of different gene lengths and sequencing discrepancy on the calculation of expression abundance. The formula is:

$$RPKM\;=\;\frac{total\;exon\;reads}{mapped\;reads\;(millions)\times exon\;length\;(KB)}$$

We set the q-value at<0.05 as the minimum false discovery rate for the statistically significance differences in gene expression.

### qPCR and data analysis

We isolated larvae, prepupae and pupae as described, collecting 30, 45, 60 silk glands from each group, with three independent biological replicates. RNA was isolated and cDNA prepared as described. qPCR was performed using the Mastercycler ep realplex (Eppendorf, Germany). Gene specific primers (Table 1) were designed using Beacon Designer 7.6 and synthesized by Sangon Biotech Co., Ltd (Shanghai, China). Glyceraldehyde 3-phosphate dehydrogenase (GAPDH) was used as a stably expressed reference gene [7]. The reaction program was: 2 min at 95°C for 15 s, 55°C for 30 s, and 72°C for 30 s. Melting curves confirmed a single gene-specific peak and the absence of primer dimmer peaks. GoTaq qPCR Master Mix (Promega, Madison, WI, USA) was used to measure the mRNA levels according to the manufacturer’s instructions. A five-fold dilution series was used to construct a relative standard curve to determine the PCR efficiencies and for quantification. Each reaction was run in triplicate (technical replications) with three independent biological replicates. Relative quantification of 8 genes was calculated by the comparative 2^-ΔΔCT^ method [8] to identify the relative mRNA levels of the samples from different life stages.

**Table 1:** Primers used for qRT-PCR validation, all with Tm = 55°C. Gene IDs are in parenthesis.

Gene	FForward primer (5’-3’)	Reverse primer (5’-3’)	Product (bp)
RIR2 (33086)	GCCCTGTGTCAAGAAGAAGG	AAATGGAGGCGAAACTACCG	131
ACSL5 (26685)	CTTCTACAGCGGCGACATTC	ACAGCGCCATATTGAACAGC	161
RPP40 (35571)	TACAGCCAGCCAGAAAGTGA	CTTTGAATGCTTATGCTTGTCC	167
XDH (53478)	CGGTGTTGGGCTTTACTACAAT	CAATGTTCCTGGTGCTCTGC	112
GLCM (16903)	AATGACGCTTGACGACCAAC	GCACCGCCACTCCATCTAT	101
BRC1 (27009)	GCCGCATCATCATAGACCAC	GCGGGAATTTAGTGTCAGCA	131
FMBP-1 (25262)	AGCACCAGCATGAGCCAATA	AACCGAACCTTGCGTTCTTC	127
ESTJ (31375)	CAGGCTCAACGTGTTTGGAT	GTGACTTCTTCCGGGTCTCC	138

### Development- and tissue-specific expression of a gene encoding a fibroin light chain (FLC)

Larvae, instars 1–5, prepupae, pupae, and adult females and males were collected (about 0.08 g tissue/sample) with 3 independent biological replicates, frozen in liquid nitrogen and kept under -70°C until analysis.

The midgut, hindgut, silk gland, fat body, epidermis, and Malpighian tubes were isolated from 5^th^ instar larvae (5 tissues per pool, 3 independent biological replicates). The larvae were washed with distilled water twice and anesthetized on ice. The pleopod was cut with dissecting scissors and hemolymph was collected with pressure on the body using a pipettor. All of the tissue samples were frozen in liquid nitrogen and kept under -70°C for analysis [9]. R NA was isolated and cDNA prepared as described. Development- and tissue-specific qPCR analysis of *FLC* expression was performed as described.

## Results

### Transcriptome sequencing and sequence assembly

Before mapping the tags, the transcriptomes were preprocessed, yielding 42,040,007 clean reads and 60,298 unigenes. All the raw tags were filtered with reference sequences, leaving 15,445,926, 13,758,154, and 16,787,792 clean reads from the larval, prepupal and pupal libraries, respectively (Table 2). Analysis of sequencing saturation showed that the number of detected genes increased until the sequencing reads reached 3 million or more, indicating the identified expressed reads were sufficient to represent the entire transcriptional information of the CLR genome.

**Table 2:** Summary statistics of RNA-seq library sequencing and mapping.

	Larvae	Prepupae	Pupae
Raw reads	15,556,751	13,851,700	17,189,289
Clean reads	15,445,926	13,758,154	16,787,792
Mapped reads	14,037,862	12,223,263	11,721,016
Mapped rate	90.88%	88.84%	69.81%
Mapped gene number	50,158	48,415	46,623
Total gene number	60,298	60,298	60,298
Mapped gene rate	83.18%	80.29%	77.32%

### Gene identification and annotation

**Fig 1:**
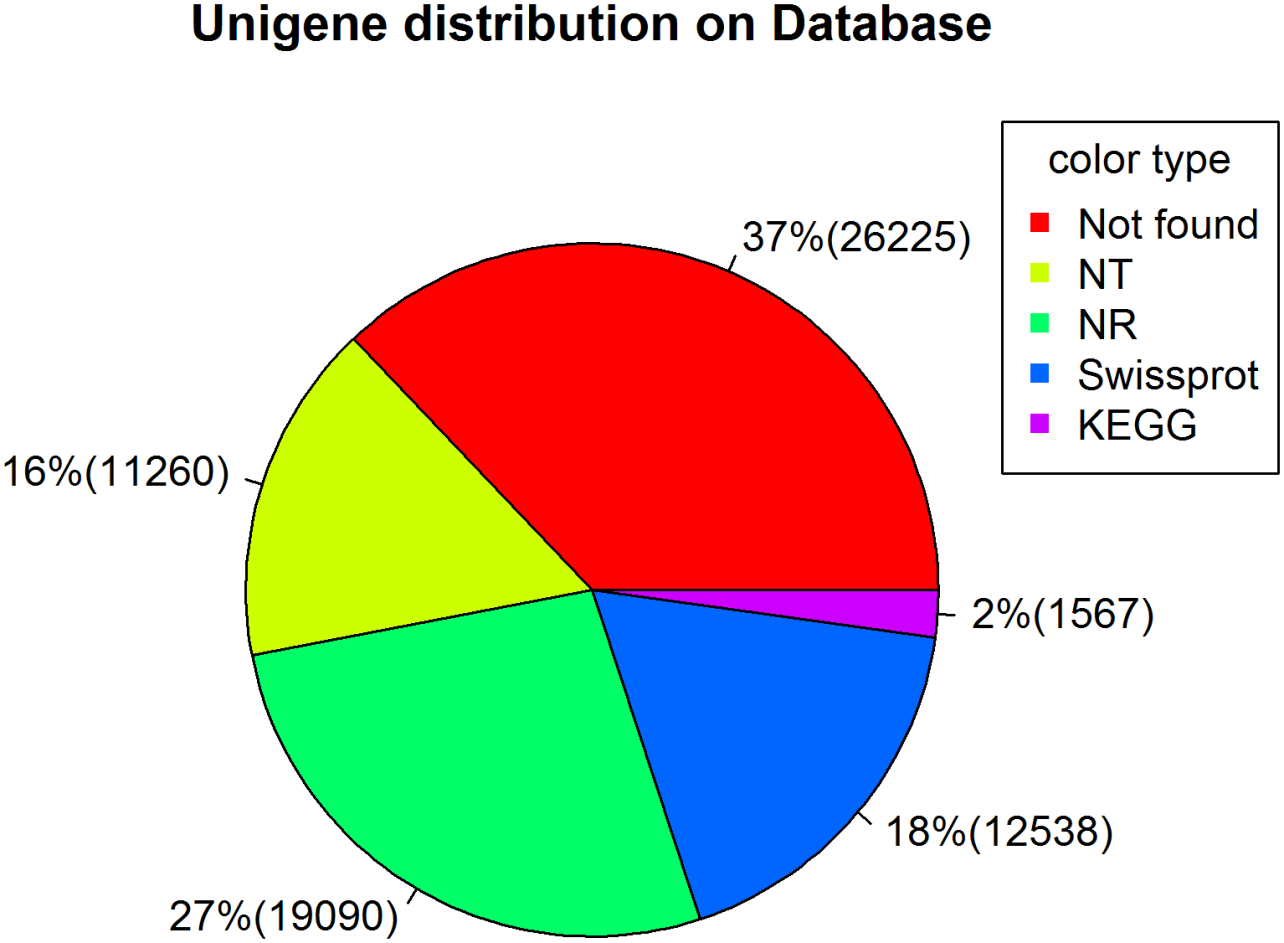
A pie chart showing proportions (with numbers in parentheses) of transcripts annotated by NR, Swiss-prot, NT and KEGG databases.

All distinct sequences longer than 200 bp were searched against the NR, Swiss-prot, and KEGG protein databases by BLASTX with a cut-off E-value of 1e^-10^. We recorded 19,090 (41.1% of all distinct sequences; by NR), 12,538 (27.0%; Swiss-prot), 11,260 (24.3%; NT) and 1,567 (3.4%; KEGG) transcripts (Fig 1). Identities of many genes (about 37%) were not found in available databases, as expected due to the lack of transcriptomic information in tissues within pest species.

### Gene expression profiles

**Fig 2:**
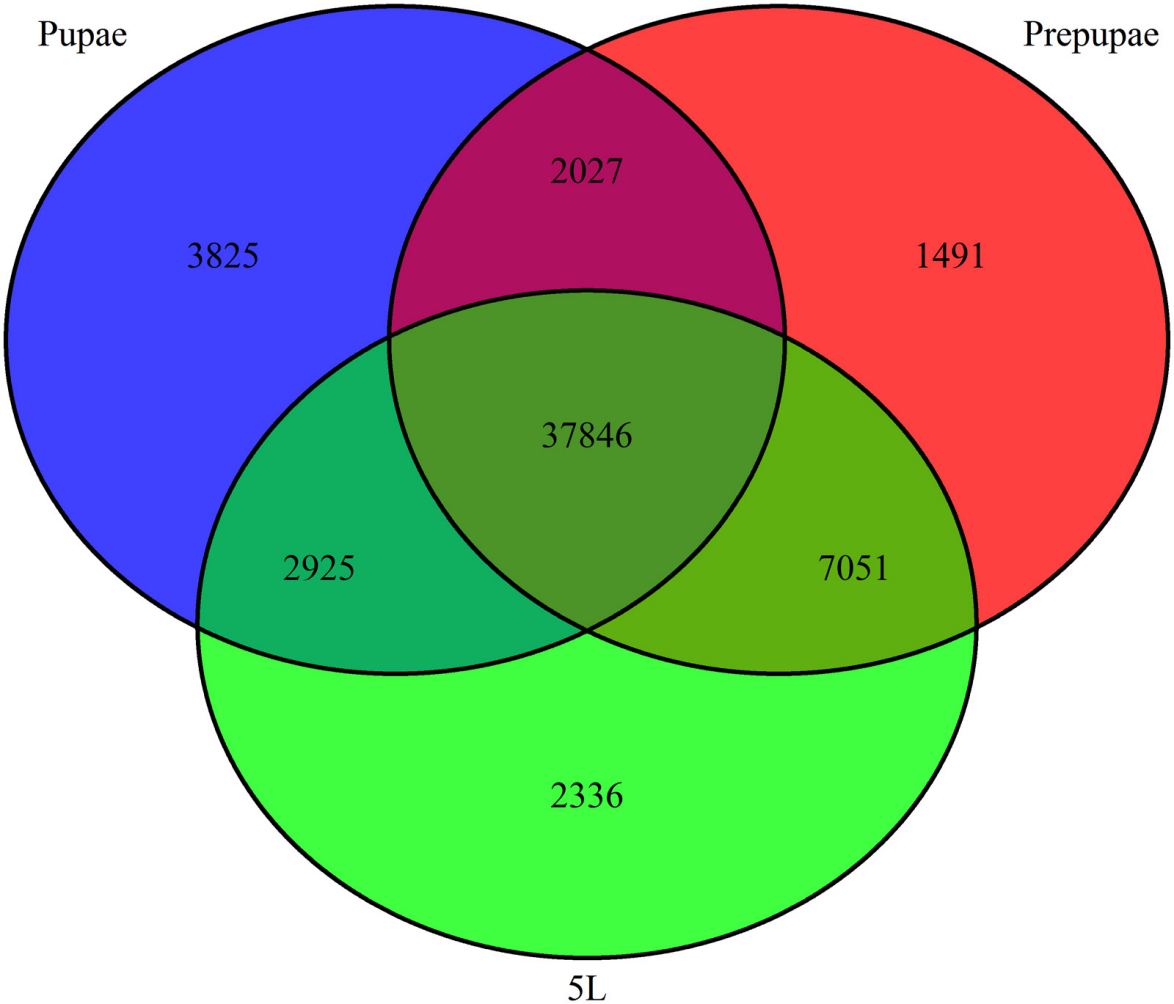
A Venn diagram showing overlap of silk gland genes expressed in larval, prepupal and pupal stages. 5L represents fifth instar larvae.

We identified 50,158, 48,415, and 46,623 transcripts from the silk gland libraries. Among these, 37,846 transcripts were expressed in all three libraries, 44,897 were expressed in larval and prepupal libraries, and 39,873 were expressed in prepupal and pupal libraries. We recorded 40,771 transcripts expressed in larvae and pupae (Fig 2).

Expression of 38 genes changed (27↑, 11↓) during the larval/prepupal transition. The prepupal-pupal transition led to expression changes in 355 genes (137↑, 218↓). The broader excursion from larvae to pupae included up-regulation of 140 and down-regulation of 169 genes (Table 3; specific genes listed in S1 Table).

**Table 3:** Differentially Expressed Genes across all libraries.

	5^th^ instar larvae: Prepupae	Prepupae:Pupae	5^th^ instar larvae:Pupae
Total	38	355	309
Up-regulated	27	137	140
Down-regulated	11	218	169

### Genes related to silk formation

We selected seven silk gland genes that changed in expression during the developmental excursion as directly or indirectly involved in silk formation (Table 4; partial sequences of specific genes listed in S2 Table). Expression of genes encoding the heavy- and light-chain fibroin proteins declined as the larvae moved beyond the silk-producing larval stage. A gene encoding a fibroin-modulator-binding protein-1 is a transcriptional regulatory factor operating in the *B*. *mori* silk gland. Expression of this gene increased by almost 3-fold as the larvae went into the pre-pupal stage. Three genes act in endocrine signaling, which we take to indirectly influence silk production via their influence on silk gland developmental physiology as the insects moved from larvae to prepupae to pupae. We included a gene encoding a phosphatidylethanolamine binding protein (PEBP) because it acts in cell signaling and may indirectly influence silk production. Expression of this gene declined by a wide extent as the larvae advanced to prepupae.

**Table 4:** Expression abundance of genes related to silk formation identified in the indicated citations. 5L = 5th instar larvae.

Gene ID	5L (RPKM)	Prepupae (RPKM)	Pupae (RPKM)	Function annotation	Cell function	Reference
8810	1089.58	255.37	123.48	fibroin heavy chain gene	Silk protein	[15]
23951	34112	25935	1604	fibroin light chain	Silk protein	[25]
25262	8.99	23.91	12.82	fibroin-modulator-binding protein-1	Metabolism	[26]
31375	16.23	215.41	1.23	juvenile hormone esterase	Endocrine signaling	[27]
43038	2.29	3.91	0.21	juvenile hormone esterase precursor	Endocrine signaling	[12]
44378	22.01	0.02	0.58	ecdysone oxidase	Endocrine signaling	[28]
33585	429.51	0.26	2.03	Phosphatidylethanolamine binding protein (PEBP)	Cell signaling	[16]

### Genes encoding transcription factors

Development from larvae to pupae was attended by changes in expression of genes encoding a range of transcription factors. Expression of 17 genes changed expression (14↑, 3↓) going from larvae to prepupae; 21 genes (14↑, 7↓) changed expression in the transition from prepupae to pupae. We listed these genes in a separate table (Table 5; specific gene sequences listed in S2 Table) to highlight the actions of their cognate proteins as transcription factors.

**Table 5:** Expression abundance of most differentially expressed transcription factors during larval to pupal development. Silk formation is highest in 5th instar larvae, lower in prepupae and does not occur in pupae.

Gene ID	Fold change of gene expression	Function annotation
**The 5** ^**th**^ **instar larvae/Prepupae**		
GFI1(37831)	-3.55	protein binding/zinc ion binding/transcription regulatory region DNA binding/metal ion binding
ARRB1(44963)	3.42	protein binding/transcription factor
PTEN(33140)	3.56	cyclin-dependent kinase 3 (CDK3)
UHRF1(15676)	-3.55	equence-specific DNA binding transcription factor activity/protein binding
APBB2(41696)	3.43	protein binding/transcription factor binding
APBB2(41695)	3.41	protein binding/transcription factor binding
MMP14(35672)	7.77	sequence-specific DNA binding transcription factor activity
MMP14(35673)	8.40	sequence-specific DNA binding transcription factor activity
MMP14(35674)	8.24	sequence-specific DNA binding transcription factor activity
MMP14(35675)	8.10	sequence-specific DNA binding transcription factor activity
MMP14(35676)	8.11	sequence-specific DNA binding transcription factor activity
KLF7(35676)	6.21	sequence-specific DNA binding transcription factor activity/transcription coactivator activity
TCP4(19504)	-4.28	putative RNA polymerase II transcriptional coactivator
HR3(37751)	6.43	molt-regulating transcription factor
HR3(37750)	6.41	molt-regulating transcription factor
HR3(37749)	7.14	molt-regulating transcription factor HHR3
HR3(37748)	7.13	molt-regulating transcription factor HHR3
**Prepupae/pupae**		
CLOCK(46823)	-4.25	RNA polymerase II core promoter proximal region sequence-specific DNA binding transcription factor activity
CLOCK(46824)	-4.34	RNA polymerase II core promoter proximal region sequence-specific DNA binding transcription factor activity
MDN1(52909)	4.22	transcription factor binding
MDN1(52910)	4.18	transcription factor binding
MDN1(52911)	4.61	transcription factor binding
MDN1(52912)	4.55	transcription factor binding
REST(43754)	4.21	RNA polymerase II core promoter proximal region sequence-specific DNA binding transcription factor
DNMT1(7480)	4.19	DNA cytosine-5 methyltransferase
KDM1A(23518)	4.12	transcription factor binding
35426	4.08	putative gonadotropin inducible transcription factor
HR3(37751)	-4.01	molt-regulating transcription factor
HR3(37750)	-3.99	molt-regulating transcription factor
HR3(37749)	-3.79	molt-regulating transcription factor HHR3
HR3(37748)	-3.78	molt-regulating transcription factor HHR3
ZNF83(44937)	-4.24	putative KRAB box and zinc finger C2H2 type domain containing protein
ZGPAT(26481)	3.93	sequence-specific DNA binding transcription factor activity
ZGPAT(26482)	3.90	sequence-specific DNA binding transcription factor activity
RTF1(50796)	3.98	hypothetical protein KGM_10227
RTF1(50798)	3.78	hypothetical protein KGM_10227
35424	3.64	putative gonadotropin inducible transcription factor
MYNN(27584)	4.92	sequence-specific DNA binding transcription factor activity

Some genes underwent very large changes in expression, 20 of which are listed in Table 6 (specific gene sequences listed in S2 Table). A gene encoding PEBP decreased in expression by about 1400-fold and expression of a gene encoding a possible serine protease inhibitor increased by over 1200-fold. Apparent development regulation of several other genes, including genes encoding a sugar transporter protein, a peroxidase, and an aldehyde oxidase I, went through similar large-scale expression changes. We list these in Table 6 to emphasize the scale of expression changes.

**Table 6:** Twenty of highly differentially expressed annotated genes in the 5th instar larvae/ prepupae and prepupae/pupae libraries based on expressed tag frequency. “-”indicates down regulation.

Gene ID	Fold Change of FPKM	Function Annotation
**5** ^**th**^ **instarlarvae/prepupae**		
33585	-1395.299	phosphatidylethanolamine binding protein isoform 2
41682	1245.514	putative serine protease inhibitor dipetalogastin precursor
31019	-1081.801	chemosensory protein
30647	906.37	yellow-d
44378	-899.634	Putative ecdysone oxidase
19984	-559.535	antennal esterase CXE10
26273	-379.028	hypothetical protein KGM_09786
20474	187.549	hypothetical protein KGM_12610
46291	172.554	AGAP010734-PA
19938	-157.702	phosphatidylethanolamine-binding protein
40494	-137.058	storage protein 1
12186	-133.29	Delta(11)-desaturase
20329	130.737	cytochrome P450
31363	102.857	storage protein
53481	87.582	putative aldehyde oxidase
53475	66.045	aldehyde oxidase 1
41137	-58.726	dermal papilla derived protein 13
52992	56.416	hypothetical protein KGM_05615
30920	56.4	RNA-directed DNA polymerase from mobile element jockey-like
**Prepupae/pupae**		
22648	1268.855	32 kDa apolipoprotein precursor
35675	1171.312	interstitial collagenase (mmp1 gene), isoform 1
47659	-1142.501	glycosyltransferase 2
49378	-1011.838	aldehyde oxidase 1
46154	-922.463	peroxidase
36399	-605.506	sugar transporter protein 3
36495	-423.661	unknownunsecreted protein
46824	-410.681	*B*.*mori* mRNA, clone: ftes09M08
20329	-394.512	cytochrome P450
45414	-359.119	serine protease inhibitor 012
45413	-338.997	serine protease inhibitor 28
31409	-279.435	alanyl-tRNAsynthetase-like
53012	-298.819	sugar transporter
16572	265.327	polyprotein-like
41322	-258.813	dopa decarboxylase (DDC)
45639	-253.115	voucher Ppy122 glucose phosphate dehydrogenase
25098	-242.412	laccase 2
33067	-229.186	WAP, kazal, immunoglobulin, kunitz and NTR domain-containing protein 2
20474	-191.098	hypothetical protein KGM_12610
45335	-186.907	GTP-binding protein sar1
30704	168.487	zinc finger protein 57

### Confirmation of gene expression

**Fig 3:**
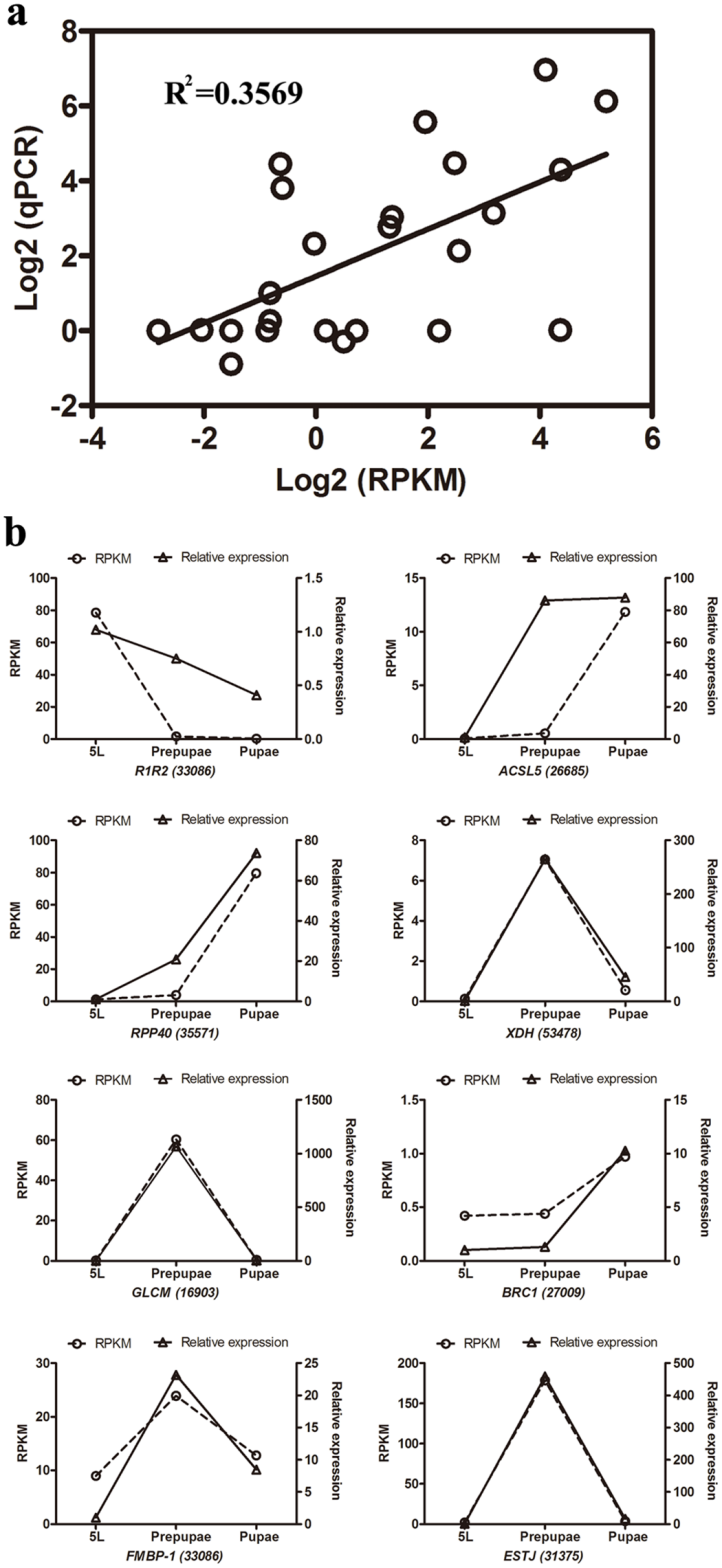
qPCR validation of 8 genes from three CLR developmental stages. (a) Comparison of expression levels measured by RNAseq and qPCR for the selected 8 transcripts in three libraries (5L, Prepupae and Pupae; R^2^ = 0.3569). (b) Comparison of expression levels of eight genes recorded by RNAseq and qPCR. Each panel shows gene expression on the left y-axis, determined by RPKM, and relative gene expression determined by qPCR on the right y-axis.

Expression analysis of 8 genes demonstrated parallel expression changes recorded by RNA-seq and qPCR (Fig 3). Of particular interest, genes encoding a xanthine dehydrogenase (XDH), a possible CRCT domain protein BRC1 (Brc1), and ribonuclease P protein subunit p40 (RPP0) were expressed in parallel.

**Fig 4:**
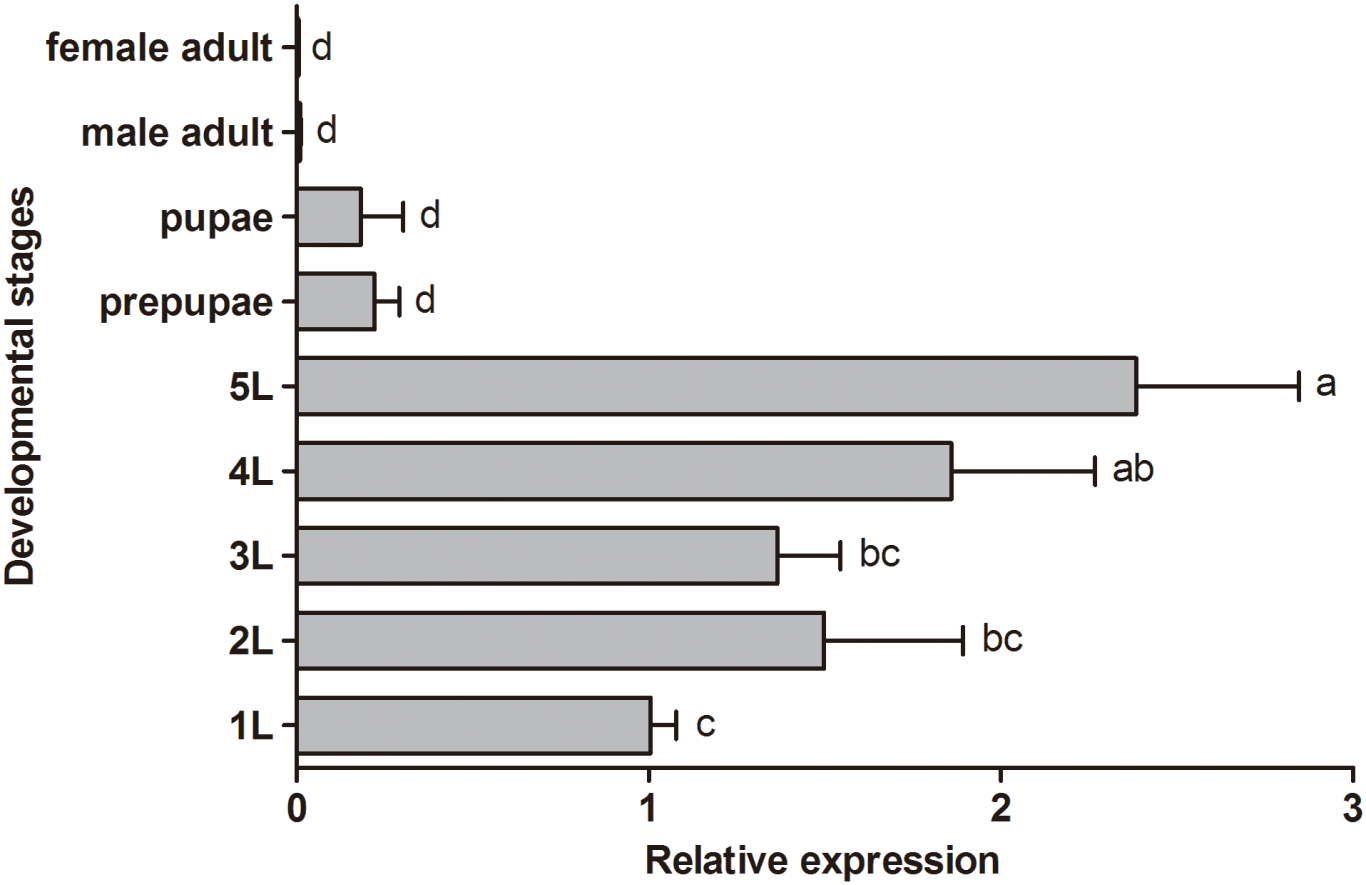
Expression pattern of a fibroin light chain gene at the indicated CLR developmental stages. Insect life stages are indicated on the vertical axis and relative gene expression (as fold-change) on the horizontal axis. Histogram bars annotated with the same letter are not significantly different.

*FLC* (ORF = 804 bp, encoding 267 amino acids) was obtained by RACE (GenBank accession number: JX456150 [10]). During the larval stage, *FLC* expression increased with larval development, reaching a peak at the 5^th^ instar, then declined during the prepupal, pupal and adult stages (Fig 4). *FLC* was expressed, at very low levels, in the midgut, hindgut, fat body, epidermis, Malpighian tubes and hemolymph. Highest expression, by about 646 to 13,249-fold, occurred in the silk gland (*F* = 20.629, df = 6, 20, *P* = 0.0001); *FLC* expression was similar among the other six tissues (*F* = 1.723, df = 5, 17, *P* = 0.2039) (Table 7).

**Table 7:** Expression pattern of fibroin light chain gene in different tissues of CLR.

Tissue	Relative expression	df	F	p
midgut	1.20±0.424 bB			
hindgut	3.67±1.995 bB			
fat body	1.89±0.465 bB			
epidermis	0.18±0.043 bB	6,20	20.629	0.0001
Malpighian tubes	1.19±0.457 bB			
silk gland	2368.95±521.214 aA			
hemolymph	1.56±0.293 bB			

## Discussion

The data reported in this paper strongly support our hypothesis that silk gland gene expression changes during the developmental transition from larvae to pupae. Three points form a solid argument. One, we generated transcriptomes from silk glands prepared from larvae, prepupae and pupae, from which we identified 355 silk gland genes that changed expression during development from 5^th^ instar larvae to pupae, understanding that not all 355 genes act directly in silk production. Two, we confirmed expressed changes in 8 genes by qPCR, indicating our RNA-seq data reliably represents changes in gene expression. Three, 38 of the expression-altered genes encode transcription factors, which would influence expression of other genes. Taken together, these points support our inference that gene expression in CLR silk glands is under strong developmental control.

In silk gland biology, the transition represents a period of high silk production in 5^th^ instar larvae, to reduced production in pre-pupae to atrophy of the silk gland in pupae. We surmise developmental regulation of gene expression makes up the proximal mechanism of these changes in silk gland biology. The biological significance of regulating silk gland gene expression touches on several areas. As seen in many areas of biology, such as development of reproductive systems, silk production is restricted to the life stages in which silk contributes to CLR biology. Overall CLR fitness would be substantially reduced due to biological costs of allocating resources toward maintaining unnecessary silk glands and to protein-costly silk production. Perhaps more to the point, unnecessary silk could be directly deleterious to CLR and many other silk-producing insect species. We infer that silk gland gene expression is tightly regulated.

The major developmental hormones, juvenile hormone (JH) and 20-ecdysteroid (20E), signal the transition from larvae to pupae and also influence silk gland biology [11]. The JH analog, methoprene, is applied to silkworms, *B*. *mori*, to extend the last larval instar and enhance silk production [6]. JH esterase is responsible for the developmentally-regulated decline in JH titers associated with pupation [12]. Our data show that JH esterase is expressed at low levels in 5^th^ instar larval silk glands, then increases by 13-fold in pre-pupal silk glands before declining to near-zero in pupae. This is consistent with high silk production during cocoon formation and subsequent atrophy of the silk gland. Similarly, ecdysone oxidase (EO) reduces 20E titers by converting 20E into inactive 3-dehydroecdysone [13]. Expression of the gene encoding EO in silk glands declined by about 1100-fold to virtually zero in the larval-pre-pupal transition. We infer the decline in silk gland EO expression is consistent with increased 20E titers during silk production and preparation for the pupal molt. Altered expression of silk gland genes encoding enzymes that regulate JH and 20E titers aligns with the actions of these hormones in silk production during development.

Gene expression is influenced by a large number of transcription factors. We recorded substantial changes in expression of genes encoding transcription factors in the larval-pupal transition. Among these, expression of 10 genes increased by > 6-fold and expression of another 10 decreased by >3-fold. Genes encoding sequence-specific DNA-binding transcription factor activities, MMP14 (35673–35676) increased by > 8-fold. These large changes in expression of an array of genes indicate the dynamic metabolic and developmental changes in the silk gland during silk production and silk gland atrophy during subsequent development into pupae. In particular, expression of the molt-regulating transcription factors HR3 (37748–37751) increased by 6- to 7-fold in the larval-pre-pupal transition, then decreased by circa 3-fold in the pre-pupal-pupal period. These proteins act in gene regulation during metamorphosis. Silk gland-specific changes in expression of these genes are associated with the changes in the structure and function of the gland during the larval-pupal metamorphosis. HR3 genes are crucial to molting physiology at the organismal level and they trigger hypotheses about the actions of these and other transcription factors in the silk gland [14].

Fibroin and sericin are the main silk proteins. Fibroin is made of heavy- and light-chain polypeptides incorporated with the 25 kDa protein, P25. Sericin is a glycoprotein responsible for agglomerating the silk proteins [15]. Silk gland expression of gene 8810, encoding a fibroin heavy chain, was highest in larvae, lower by about 4.3-fold in pre-pupae and still lower, by about 9-fold, in pupae. We recorded similar data for a *FLC*, high expression in larvae, slightly lower in pre-pupae and reduced by about 21-fold in pupae. This pattern is consistent with the silk-producing biology of the silk gland.

Our qPCR analysis shows *FLC* expression gradually increased during larval development to a peak at the 5^th^ instar. Because older larvae depend on silk, we infer the *FLC* expression pattern is consistent with the silk-producing and leave-rolling behavior of CLR larvae. Similarly, silk gland *FLC* expression was much higher compared to other tissues. Again, our interpretation is *FLC* acts in silk production.

Developmental regulation of silk gland gene expression led to very large changes in some genes. These rather striking changes are not unexpected because the silk glands undergo fundamental changes from metabolically active, silk-producing tissues to regulated atrophy in a limited time frame. The functions of some genes, such as yellow-d (30647), are not yet known well enough to generate meaningful commentary. Changes in other genes lead to useful hypotheses about their specific functions in the context of silk gland biology. The purpose of this first description of silk gland transcriptomes is to report on the large changes in gene expression. Detailed discussion of each gene would amount to speculations and we limit our discussion of individual genes to a phosphatidylethanolamine binding protein (PEBP), a peroxidase and a chemosensory protein.

PEBPs form a protein family, members of which occur in a very wide range of organisms, including bacteria, plants and animals [16]. These proteins are mostly signal moieties with substantial importance in biomedicine [17]. Silk gland gene 33585 encodes a PEBP that declined in expression (by almost 1400-fold) during the larval-pre-pupal excursion. The biological functions of PEBP in insects are not fully appreciated, but they probably act in intracellular signaling roles, as reported in biomedical research. Reumer et al. [18] discovered the *Drosophila* PEBP1 (CG18594) is expressed in larval fat bodies and in the *Drosophila* larval hemocyte cell line, l(2)mbn. The authors investigated the idea that PEBP conveys immune protection against bacterial infections. They showed that larvae overexpressing PBEP1 synthesized and released increased anti-microbial proteins into the hemolymph and that infection stimulated a 6.5-fold increase in PBEP1 expression. They inferred that *Drosophila* PEBP1 acts in immunity. We do not yet appreciate the specific role of this gene in silk glands, but surmise that PEBP acts in various signaling functions.

Expression of a peroxidase (POX; 46154) was down-regulated by nearly 1,000-fold in the transition from prepupae to pupae. POXs act in many areas of insect biology, including cuticle tanning and anti-oxidant metabolism. A new role of POXs has recently come to light. POXs also generate prostaglandins (PGs) that mediate follicle maturation [19] and coordinate expression of eggshell genes in *Drosophila* [20]. Most recently, Park et al. [21] reported that POXs act in PGE_2_-mediated immune reactions to infection. We speculate that PGs act in silk gland physiology and down-regulation of POX is congruent with the loss of function and break down of the silk gland.

Insects sense chemicals via soluble proteins, which act by binding air-borne chemicals, such as sex pheromones and plant volatiles. The soluble proteins, bound with their signal ligands, interact with specific chemoreceptors located on sensory nerve membranes, which leads to the transduction from chemical to electrical signals and subsequent integration within the central nervous system [22]. Insects express two families of soluble olfactory proteins, odorant binding proteins (OBPs) and chemosensory proteins (CSPs). CSPs are smaller than OBPs (100–120, compared to 150–160 amino acids), although they act in similar functions [22]. CSPs are expressed in antennae, but also in other, non-sensory, tissues and body parts, such as antennae-removed heads, thoraces, abdomens, gonads, wings and pheromone glands [23]. The broad distribution of these proteins led to suggestions that CSPs act in additional roles, possibly female reproduction, limb regeneration and embryonic development. We report that silk glands express one gene encoding a CSP, 31019, which declined in expression by about 1,000-fold during the larval-pre-pupal transition. We infer this protein acts in the silk gland during the silk-producing phase of pupation, possibly directly in silk production.

Arthropods, including pest insects, use silk in a very wide range of biological functions, including food capture, nests, guidelines, dispersal, reproduction, egg coatings and protective cocoons. Some silk-producing insects influence the structure of their environments. For an unusual example, the aquatic insect *Hydropsyche siltalai* (Tricoptera, Hydropsychidae, net-spinning caddisflies) produces silk cup-like nets to capture small prey organisms. Statzner et al. [24] reported that their silk is fixed to gravel pieces that results in gravel consolidation in stream beds, which influences sediment erosion. The concept that insect silk production can influence local environments prompts our suggestion of substantial research into pest insect silk production.

## Supporting Information

S1 Table Differentially Expressed Genes across all libraries. (DOC)

S2 Table Gene IDs and the partial sequences. (DOC)

## References

[1] Wang MQ, Cai WZ (2004) Silk and silk glands of insects. Entomol. Knowl 40(1): 90–95.

[2] Klapwijk MJ, Csóka G, Hirka A, Björkman C (2013) Forest insects and climate change: long-term trends in herbivore damage. Ecol Evol 3(12): 4183–4196. 10.1002/ece3.717 24324869 PMC3853563

[3] Xu J, Wang QX, Wu JC (2010) Resistance of cultivated rice varieties to *Cnaphalocrocis medinalis* (Lepidoptera: Pyralidae). J Econ Entomol 103(4):1166–1171. 20857724 10.1603/ec09265

[4] Di JX, Chen XS, Wu QJ, Xu NY, Xiao SH, Liu JG, et al (2007) Preliminary study of damage caused by *Sylepta derogate* Fabricius (Lepidoptera: Pyralidae). Jiangsu Agricultural Sciences 5: 82–84.

[5] Hou Y, Xia QY, Zhao P, Zou Y, Liu HL, Guan J, et al (2007) Studies on middle and posterior silk glands of silk worm (*Bombyx mori*) using two dimensional electrophoresis and mass spectrometry. Insect Biochem Molec 37: 486–496. 10.1016/j.ibmb.2007.02.011 17456443

[6] Sehnal F, Akai H (1990) Insect silk glands: their types, development and function, and effects of environmental factors and morphogenetic hormones on them. Int J Insect Morphol Embryol 19 (2): 79–132.

[7] Zheng YT, Li HB, Lu MX, Du YZ (2014) Evaluation and validation of reference genes for qRT-PCR normalization in *Frankliniella occidentalis* (Thysanoptera: Thripidae). PLoS One 9(10): e111369 10.1371/journal.pone.0111369 25356721 PMC4214748

[8] Livak KJ, Schmittgen TD (2001) Analysis of relative gene expression data using real-time quantitative PCR and the 2^(-ΔΔ C(T))^ method. Methods 25(4): 402–408. 11846609 10.1006/meth.2001.1262

[9] Wang X (2011) Cloning and expression of Csaqp1genes in rice stem borer, *Chilo Suppressalis* Walker (Lepidoptera: Pyralidae). M.Sc. Thesis, Yangzhou University. Available: http://www.shangxueba.com/lunwen/v1473369.html. Accessed 19 December 2014.

[10] Chen CX, Wang ZY, Gu GX, Yang YZ, Liang GH (2015). Cloning of a silk-producing gene in the cotton leaf roller *Sylepta derogata* (Lepidoptera: Pyralidae) and its function. J Environ Entomol: In press.

[11] Zhao XM, Liu C, Jiang LJ, Li QY, Zhou MT, Cheng TC, et al (2014) A juvenile hormone-transcription factor Bmdimm-fibroin H chain pathway is involved in the synthesis of silk protein in silkworm, *Bombyx mori* . J Biol Chem Pii:jbc.M114 606921. [Epub ahead of print] 10.1074/jbc.M114.606921 PMC4294524 25371208

[12] Kamimura M, Takahashi M, Kikuchi K, Reza AM, Kiuchi M (2007) Tissue-specific regulation of juvenile hormone esterase gene expression by 20-hydroxyecdysone and juvenile hormone in *Bombyx mori* . Arch Insect Biochem Physiol 65(3):143–151. 17570489 10.1002/arch.20186

[13] Yang HJ, Wang MX, Zhang P, Sabhar A, Malik FA, Bhaskar R, et al (2011) Cloning and characterization of the *Bombyx mori* ecdysone oxidase. Arch Insect Biochem Physiol 78: 17–29. 10.1002/arch.20436 21678487

[14] Xiong Y, Zeng H, Zhang Y, Xu D, Qiu D (2013) Silencing the HaHR3 gene by transgenic plant-mediated RNAi to disrupt *Helicoverpa armigera* development. Int J Biol Sci 9(4): 370–381. 10.7150/ijbs.5929 23630449 PMC3638292

[15] Shimizu K, Ogawa S, Hino R, Adachi T, Tomita M, Yoshizato K (2007) Structure and function of 5 '-flanking regions of *Bombyx mori* fibroin heavy chain gene: Identification of a novel transcription enhancing element with a homeodomain protein-binding motif. Insect Biochem Mol Biol 37(7): 713–725. 17550827 10.1016/j.ibmb.2007.03.016

[16] Li H, Huang F, Fan L, Jiang Y, Wang X, Li J, et al (2014) Phosphatidylethanolamine-binding protein 4 is associated with breast cancer metastasis through Src-mediated Akt tyrosine phosphorylation. Oncogene 33(37): 4589–4598. 10.1038/onc.2013.408 24276246

[17] Zhao J, O’Donnell VB, Balzar S, Croix CM St., Trudeau JB, Wenzel SE (2011) 15-lipoxgnease 1 interacts with phosphatidylethanolamine-binding protein to regulate MAPK signaling in human airway epithelial cells. Proc Natl Acad Sci USA 108(34): 14246–14251. 10.1073/pnas.1018075108 21831839 PMC3161579

[18] Reumer A, Bogaerts A, Van Loy T, Husson SJ, Temmerman L, Choi C, et al (2009) Unraveling the protective effect of a *Drosophila* phosphatidylethanolamine-binding protein upon bacterial infection by means of proteomics. Dev Comp Immunol 33: 1186–1195. 10.1016/j.dci.2009.06.010 19545586

[19] Tootle TL, Spradling AC (2008) *Drosophila* Pxt: a cyclooxygenase-like facilitator of follicle maturation. Develop 135: 839–847. 10.1242/dev.017590 PMC2818214 18216169

[20] Tootle TL, Williams D, Hubb A, Frederick R, Spradling A (2011) *Drosophila* eggshell production: identification of new genes and coordination by Pxt. PLoS One 6: e19943 10.1371/journal.pone.0019943 21637834 PMC3102670

[21] Park J, Stanley D, Kim Y (2014) Role of peroxinectin in PGE_2_-mediated cellular immunity in *Spodoptera exigua* . PLoS One 9: e105717 10.1371/journal.pone.0105717 25191834 PMC4156296

[22] Pelosi P, Lovinella I, Felicioli A, Dani FR (2014) Soluble proteins of chemical communication: an overview across arthropods. Front Physiol 5: 320 10.3389/fphys.2014.00320 25221516 PMC4145409

[23] Qiao HL, Deng PY, Li DD, Chen M, Jiao ZJ, Liu ZC, et al (2013) Expression analysis and binding experiments of chemosensory proteins indicate multiple roles in *Bombyx mori* . J Insect Physiol 59(7): 667–675. 10.1016/j.jinsphys.2013.04.004 23624070

[24] Statzner B, Arens MF, Champagne JY, Morel R, Herouin E (1999) Silk-producing stream insects and gravel erosion: significant biological effects on critical shear stress. Water Resources Res 35: 3495–3506.

[25] Chaitanya RK, Dutta-Gupta A (2010) Light chain fibroin and P25 genes of *Corcyra cephalonica*: Molecular cloning, characterization, tissue-specific expression, synchronous developmental and 20-hydroxyecdysone regulation during the last instar larval development. Gen Comp Endocrinol 167(1):113–121. 10.1016/j.ygcen.2010.02.007 20171223

[26] Takiya S, Saito S, Yokoyama T, Matsumoto D, Aizawa T, Kamiya M, et al (2009) DNA-binding property of the novel DNA-binding domain STPR in FMBP-1 of the silkworm *Bombyx mori* . J Biochem 146 (1):103–111. 10.1093/jb/mvp053 19304790

[27] Kontogiannatos D, Swevers L, Maenaka K, Park EY, Iatrou K, Kourti A (2013) Functional characterization of a juvenile hormone esterase related gene in the moth *Sesamia nonagrioides* through RNA interference. PLoS One 20138 (9): e73834. 10.1371/journal.pone.0073834 PMC3770702 24040087

[28] Sun W, Shen YH, Qi DW, Xiang ZH, Zhang Z (2012) Molecular cloning and characterization of Ecdysone oxidase and 3-dehydroecdysone-3α-reductase involved in the ecdysone inactivation pathway of silkworm, Bombyx mori. Int J Biol Sci 8(1):125–138. 22215981 10.7150/ijbs.8.125 PMC3248655

