# Supplementary material for: Silk Gland Gene Expression during Larval-Pupal Transition in the Cotton Leaf Roller Sylepta derogata (Lepidoptera: Pyralidae)
Source: PLoS One. 2015 Sep 9;10(9):e0136868. doi: 10.1371/journal.pone.0136868 (PMC4564283; doi:10.1371/journal.pone.0136868)
Supplement: S1 Table — (DOC) [file pone.0136868.s001.doc]

Supporting Information Table S1. Differentially Expressed Genes across all libraries

| Gene_Id | Gene Name | 5L（FPKM） | Prepupae (FPKM) | q value |
| --- | --- | --- | --- | --- |
| Up-regulated |  |  |  |  |
| Total.Unigene_16903 | GLCM | 0.222472 | 60.3561 | 0.002882 |
| Total.Unigene_20329 | CP18A | 4.08956 | 534.658 | 0.018793 |
| Total.Unigene_20474 | CYB5 | 0.207503 | 38.917 | 0.004669 |
| Total.Unigene_28757 | S12A6 | 0.052469 | 2.89643 | 0.037659 |
| Total.Unigene_30647 | YELL | 0.291658 | 264.35 | 0.000143 |
| Total.Unigene_30920 | GAGXE | 1.75097 | 98.7546 | 0.049572 |
| Total.Unigene_31363 | BJSB2 | 0.184834 | 19.0115 | 0.013231 |
| Total.Unigene_41682 | DPGN | 9.10861 | 11344.9 | 0.000893 |
| Total.Unigene_43463 | PERO | 0.170723 | 32.8834 | 0.004669 |
| Total.Unigene_43464 | PERO | 0.130646 | 30.1894 | 0.004267 |
| Total.Unigene_46290 | PERO | 0.374698 | 61.726 | 0.006868 |
| Total.Unigene_46291 | PERO | 0.35643 | 61.5034 | 0.006194 |
| Total.Unigene_50775 | LIPR2 | 0.249515 | 29.0111 | 0.011637 |
| Total.Unigene_50776 | LIPR2 | 0.230101 | 27.8292 | 0.010711 |
| Total.Unigene_50777 | LIPR2 | 0.238626 | 23.1409 | 0.015082 |
| Total.Unigene_50778 | LIPR2 | 0.217411 | 21.8257 | 0.014048 |
| Total.Unigene_51614 | ARSB | 0.406318 | 22.0668 | 0.03801 |
| Total.Unigene_52992 | LIP3 | 0.251938 | 14.2134 | 0.037466 |
| Total.Unigene_53471 | ADO | 0.133521 | 8.72095 | 0.028471 |
| Total.Unigene_53472 | ADO | 0.134895 | 8.43539 | 0.030707 |
| Total.Unigene_53473 | ADO | 0.131327 | 7.98002 | 0.032343 |
| Total.Unigene_53474 | ADO | 0.112503 | 7.64831 | 0.026624 |
| Total.Unigene_53475 | XDH | 0.107276 | 7.08505 | 0.028052 |
| Total.Unigene_53476 | XDH | 0.139627 | 7.58469 | 0.037459 |
| Total.Unigene_53477 | XDH | 0.116534 | 6.97232 | 0.033442 |
| Total.Unigene_53478 | XDH | 0.128446 | 7.05644 | 0.037324 |
| Total.Unigene_53481 | XDH | 0.893512 | 78.2555 | 0.018028 |
| Down-regulated |  |  |  |  |
| Total.Unigene_12186 | ACO11 | 37.6645 | 0.282576 | 0.008701 |
| Total.Unigene_19938 | PEBPH | 286.042 | 1.81381 | 0.006907 |
| Total.Unigene_19984 | EST1 | 34.8503 | 0.062284 | 0.002759 |
| Total.Unigene_26273 | ELOV7 | 213.775 | 0.564008 | 0.001551 |
| Total.Unigene_31019 | PEB3 | 465.775 | 0.430555 | 9.53E-05 |
| Total.Unigene_33585 | OV16 | 400.783 | 0.287238 | 0.000462 |
| Total.Unigene_39314 | DHGL | 19.0064 | 0.139538 | 0.008821 |
| Total.Unigene_40494 | ARYA | 15.2245 | 0.111081 | 0.022917 |
| Total.Unigene_41137 | CG010 | 34.2133 | 0.582589 | 0.034641 |
| Total.Unigene_44378 | DHGL | 20.5345 | 0.022825 | 0.01293 |
| Total.Unigene_44379 | ALKJ | 23.6915 | 0.081513 | 0.0474 |

| Gene_Id | Gene Name | 5L（FPKM） | Pupae (FPKM) | q value |
| --- | --- | --- | --- | --- |
| Up-regulated |  |  |  |  |
| Total.Unigene_15686 | CORO7 | 0.092656 | 14.004 | 0.04494 |
| Total.Unigene_16560 | MOV10 | 0.258793 | 18.9982 | 0.02318 |
| Total.Unigene_17067 | KEN2 | 0.492501 | 25.7144 | 0.039954 |
| Total.Unigene_17609 | PHTF | 0.088939 | 15.828 | 0.036675 |
| Total.Unigene_18130 | TRH | 0.196402 | 14.1353 | 0.027265 |
| Total.Unigene_18135 | CI030 | 0.263406 | 20.6811 | 0.023291 |
| Total.Unigene_18256 | BGB | 0.03433 | 12.8031 | 0.013823 |
| Total.Unigene_19683 | MYO9A | 0.281264 | 32.5728 | 0.015625 |
| Total.Unigene_20335 | ABCG1 | 0.135318 | 42.8959 | 0.002892 |
| Total.Unigene_20701 | RTXE | 0.613319 | 28.2359 | 0.049469 |
| Total.Unigene_20732 | RTXE | 0.811647 | 38.2355 | 0.048404 |
| Total.Unigene_20839 | MYO9A | 0.259257 | 16.9968 | 0.034222 |
| Total.Unigene_21187 | RTXE | 0.229916 | 15.9456 | 0.028845 |
| Total.Unigene_21390 | LIMK1 | 0.323132 | 45.8254 | 0.007836 |
| Total.Unigene_22359 | POL | 0.032922 | 43.6836 | 0.024938 |
| Total.Unigene_23123 | CFDP2 | 0.237376 | 12.3294 | 0.04003 |
| Total.Unigene_23375 | CUP52 | 0.103547 | 31.9098 | 0.018028 |
| Total.Unigene_25042 | TRX | 1.78241 | 177.588 | 0.022149 |
| Total.Unigene_25125 | TC3A | 0.080174 | 26.1759 | 0.005072 |
| Total.Unigene_25783 | YI31B | 0.203582 | 9.49207 | 0.049505 |
| Total.Unigene_25838 | ADA10 | 0.997565 | 51.9228 | 0.047693 |
| Total.Unigene_26290 | CCDCX | 0.405862 | 33.4062 | 0.020386 |
| Total.Unigene_26291 | CCDCX | 0.479815 | 34.7361 | 0.026292 |
| Total.Unigene_26685 | ACSL5 | 0.055784 | 11.853 | 0.005564 |
| Total.Unigene_26710 | DSCL | 0.037503 | 2.50257 | 0.03074 |
| Total.Unigene_26711 | DSCL | 0.043892 | 2.50735 | 0.036954 |
| Total.Unigene_26712 | DSCL | 0.038185 | 2.46137 | 0.032602 |
| Total.Unigene_26713 | DSCL | 0.044694 | 2.46604 | 0.038597 |
| Total.Unigene_26715 | DSCL | 0.020432 | 3.09743 | 0.034286 |
| Total.Unigene_26717 | DSCL | 0.020981 | 3.05253 | 0.036007 |
| Total.Unigene_26736 | ZFP26 | 0.380043 | 53.7098 | 0.01159 |
| Total.Unigene_27311 | GTPB2 | 0.171713 | 8.57066 | 0.04593 |
| Total.Unigene_27312 | GTPB2 | 0.153435 | 10.4808 | 0.032445 |
| Total.Unigene_27584 | MYNN | 0.872014 | 42.9923 | 0.047693 |
| Total.Unigene_28125 | 41 | 0.177972 | 14.5556 | 0.019322 |
| Total.Unigene_29116 | ACO11 | 0.057724 | 13.5588 | 0.008927 |
| Total.Unigene_29117 | ACO11 | 0.057984 | 13.7715 | 0.008737 |
| Total.Unigene_29651 | PERQ1 | 1.06113 | 98.8347 | 0.020565 |
| Total.Unigene_29652 | PERQ1 | 1.02672 | 89.3033 | 0.022724 |
| Total.Unigene_30009 | ZN323 | 0.39503 | 35.6173 | 0.016633 |
| Total.Unigene_30010 | ZN323 | 0.301188 | 41.2413 | 0.008288 |
| Total.Unigene_30439 | ASM | 0.119734 | 6.18396 | 0.041791 |
| Total.Unigene_30441 | ASM | 0.110284 | 13.8181 | 0.022917 |
| Total.Unigene_30442 | ASM | 0.162138 | 11.4969 | 0.034269 |
| Total.Unigene_30704 | ZG20 | 0.474057 | 56.5282 | 0.010571 |
| Total.Unigene_31101 | HSP68 | 0.055703 | 10.9336 | 0.032803 |
| Total.Unigene_31236 | ZG26 | 0.967177 | 78.4197 | 0.020401 |
| Total.Unigene_31363 | BJSB2 | 0.184834 | 20.3774 | 0.011904 |
| Total.Unigene_31393 | TCRG1 | 0.455525 | 43.7885 | 0.015626 |
| Total.Unigene_31395 | TCRG1 | 0.56114 | 60.0678 | 0.013243 |
| Total.Unigene_31396 | TCRG1 | 0.525159 | 61.2814 | 0.011569 |
| Total.Unigene_31469 | MMP25 | 0.044429 | 4.30804 | 0.018179 |
| Total.Unigene_31470 | MMP17 | 0.047742 | 4.21699 | 0.023999 |
| Total.Unigene_32569 | DCR1 | 0.5295 | 48.3636 | 0.017702 |
| Total.Unigene_32651 | VAT1L | 0.545103 | 48.1086 | 0.020527 |
| Total.Unigene_32807 | H1B | 0.224619 | 12.3831 | 0.037064 |
| Total.Unigene_32811 | H1B | 0.342806 | 16.8478 | 0.045083 |
| Total.Unigene_33335 | C3390 | 0.253203 | 15.9786 | 0.032642 |
| Total.Unigene_33661 | AZI1 | 0.110502 | 10.1076 | 0.019855 |
| Total.Unigene_33662 | AZI1 | 0.087453 | 8.64008 | 0.033202 |
| Total.Unigene_35126 | TLN2 | 0.063285 | 20.9931 | 0.002773 |
| Total.Unigene_35164 | AFAD | 0.644231 | 57.5525 | 0.017875 |
| Total.Unigene_35165 | AFAD | 0.534911 | 59.4973 | 0.012113 |
| Total.Unigene_35571 | RPP40 | 1.19283 | 79.5545 | 0.033418 |
| Total.Unigene_35621 | MANBA | 0.192237 | 10.2116 | 0.038791 |
| Total.Unigene_35672 | MMP14 | 0.054488 | 242.038 | 3.17E-06 |
| Total.Unigene_35673 | MMP14 | 0.133513 | 368.772 | 7.55E-06 |
| Total.Unigene_35674 | MMP14 | 0.086281 | 346.773 | 3.51E-06 |
| Total.Unigene_35675 | MMP14 | 0.100103 | 369.385 | 3.51E-06 |
| Total.Unigene_35676 | MMP14 | 0.104509 | 366.876 | 3.54E-06 |
| Total.Unigene_36466 | WDR65 | 0.167749 | 44.5777 | 0.002652 |
| Total.Unigene_36467 | WDR65 | 0.244047 | 67.9351 | 0.0025 |
| Total.Unigene_38151 | CHIA | 0.095992 | 110.526 | 0.000108 |
| Total.Unigene_38152 | CHIA | 0.100602 | 113.543 | 0.000112 |
| Total.Unigene_39868 | ARIP4 | 0.794572 | 70.6625 | 0.023886 |
| Total.Unigene_39869 | ARIP4 | 0.595124 | 52.1633 | 0.020003 |
| Total.Unigene_39889 | Y9776 | 0.648456 | 33.3249 | 0.044074 |
| Total.Unigene_40052 | CHS2 | 0.615464 | 50.969 | 0.021695 |
| Total.Unigene_40753 | FAF1 | 1.14334 | 113.462 | 0.017462 |
| Total.Unigene_40754 | FAF1 | 1.16042 | 120.852 | 0.015997 |
| Total.Unigene_40884 | U183 | 0.927794 | 54.1696 | 0.038597 |
| Total.Unigene_4300 | CF168 | 0.122311 | 39.133 | 0.002882 |
| Total.Unigene_43602 | CB029 | 0.327349 | 19.2048 | 0.034228 |
| Total.Unigene_44419 | MOT12 | 0.112927 | 6.00941 | 0.038597 |
| Total.Unigene_44420 | MOT12 | 0.115806 | 7.27391 | 0.030723 |
| Total.Unigene_45172 | HEP2 | 0.112734 | 6.12208 | 0.041781 |
| Total.Unigene_45263 | YMD3 | 0.156663 | 46.4336 | 0.018811 |
| Total.Unigene_45905 | GAGXE | 0.652748 | 30.798 | 0.04956 |
| Total.Unigene_45929 | PPID | 0.663487 | 42.3257 | 0.034184 |
| Total.Unigene_46045 | SOX14 | 0.866743 | 177.823 | 0.006189 |
| Total.Unigene_46196 | HTS | 0.285048 | 13.9334 | 0.046037 |
| Total.Unigene_46197 | HTS | 0.287585 | 14.0907 | 0.045835 |
| Total.Unigene_47020 | RSBNL | 0.263628 | 22.1114 | 0.019494 |
| Total.Unigene_47021 | RSBNL | 0.26572 | 22.2869 | 0.019494 |
| Total.Unigene_47022 | RSBNL | 0.266779 | 22.3343 | 0.019548 |
| Total.Unigene_47023 | RSBNL | 0.236895 | 21.7124 | 0.016369 |
| Total.Unigene_47027 | CADE | 0.099386 | 5.79956 | 0.035007 |
| Total.Unigene_47994 | LIMA1 | 0.039226 | 5.15861 | 0.012723 |
| Total.Unigene_48060 | POL4 | 0.532044 | 56.9158 | 0.013838 |
| Total.Unigene_48179 | RBCC1 | 0.640218 | 41.6344 | 0.034582 |
| Total.Unigene_48180 | RBCC1 | 0.514203 | 31.9806 | 0.033611 |
| Total.Unigene_48181 | RBCC1 | 0.865031 | 75.8667 | 0.020092 |
| Total.Unigene_48496 | SCLXB | 0.114838 | 14.2152 | 0.010197 |
| Total.Unigene_48497 | SCLXB | 0.11538 | 14.21 | 0.010351 |
| Total.Unigene_48498 | SCLXB | 0.118062 | 14.0621 | 0.010966 |
| Total.Unigene_48499 | SCLXB | 0.118635 | 14.056 | 0.01107 |
| Total.Unigene_4889 | RNO | 1.00907 | 64.0552 | 0.03803 |
| Total.Unigene_48935 | YG31B | 0.486886 | 53.4995 | 0.01372 |
| Total.Unigene_48936 | POL2 | 0.21658 | 23.9533 | 0.011933 |
| Total.Unigene_49057 | ZNF83 | 0.567284 | 28.8674 | 0.046437 |
| Total.Unigene_49058 | ZNF83 | 0.5732 | 29.1122 | 0.046649 |
| Total.Unigene_49388 | RTXE | 0.303356 | 16.4921 | 0.038017 |
| Total.Unigene_49390 | RTXE | 0.312273 | 18.7358 | 0.033137 |
| Total.Unigene_52909 | MDN1 | 0.488669 | 24.9977 | 0.048902 |
| Total.Unigene_52910 | MDN1 | 0.490124 | 25.5371 | 0.0474 |
| Total.Unigene_52911 | MDN1 | 0.512691 | 33.0534 | 0.033004 |
| Total.Unigene_52912 | MDN1 | 0.515503 | 34.07 | 0.031624 |
| Total.Unigene_53275 | VINC | 0.033253 | 3.11357 | 0.01754 |
| Total.Unigene_53276 | VINC | 0.040256 | 3.02286 | 0.023683 |
| Total.Unigene_53277 | VINC | 0.028812 | 3.31721 | 0.013838 |
| Total.Unigene_53278 | VINC | 0.036383 | 3.22591 | 0.01938 |
| Total.Unigene_53706 | PIF1 | 0.263689 | 15.563 | 0.036589 |
| Total.Unigene_53707 | PIF1 | 0.272551 | 15.6745 | 0.037741 |
| Total.Unigene_53708 | PIF1 | 0.265236 | 15.6595 | 0.036577 |
| Total.Unigene_53709 | PIF1 | 0.272147 | 15.7656 | 0.037377 |
| Total.Unigene_53710 | PIF1 | 0.277871 | 16.5984 | 0.035689 |
| Total.Unigene_53711 | PIF1 | 0.272165 | 16.6271 | 0.034587 |
| Total.Unigene_53712 | PIF1 | 0.286486 | 16.72 | 0.037049 |
| Total.Unigene_53713 | PIF1 | 0.277483 | 16.7081 | 0.035364 |
| Total.Unigene_53714 | PIF1 | 0.279562 | 16.7502 | 0.03552 |
| Total.Unigene_53715 | PIF1 | 0.27054 | 16.7341 | 0.033938 |
| Total.Unigene_53716 | PIF1 | 0.286163 | 16.8291 | 0.036765 |
| Total.Unigene_53717 | PIF1 | 0.280383 | 16.8589 | 0.035364 |
| Total.Unigene_53718 | RTBS | 0.307096 | 19.8171 | 0.031629 |
| Total.Unigene_53719 | RTBS | 0.326906 | 21.5652 | 0.030346 |
| Total.Unigene_53720 | RTBS | 0.319485 | 21.6136 | 0.028876 |
| Total.Unigene_54256 | SCAPE | 0.719298 | 68.0012 | 0.021046 |
| Total.Unigene_54259 | SCAPE | 1.29971 | 114.462 | 0.022271 |
| Total.Unigene_54514 | RTXE | 0.662895 | 40.7079 | 0.031595 |
| Total.Unigene_55648 | RTBS | 0.183304 | 12.9528 | 0.028011 |
| Down-regulated |  |  |  |  |
| Total.Unigene_16362 | CUD5 | 18.7084 | 0.243192 | 0.030688 |
| Total.Unigene_17806 | MOGT2 | 21.334 | 0.422986 | 0.042152 |
| Total.Unigene_17941 | DPGN | 16443.6 | 82.1916 | 0.025533 |
| Total.Unigene_18697 | ATPK | 1239.02 | 17.0544 | 0.037049 |
| Total.Unigene_19253 | PERO | 25.0354 | 0.266346 | 0.015564 |
| Total.Unigene_19938 | PEBPH | 286.042 | 0.279472 | 0.000216 |
| Total.Unigene_19984 | EST1 | 34.8503 | 0.086371 | 0.002126 |
| Total.Unigene_20048 | TM9S4 | 29.0606 | 0.416363 | 0.027001 |
| Total.Unigene_20150 | PRS42 | 312.435 | 0.748683 | 0.001067 |
| Total.Unigene_20151 | TM11F | 273.149 | 0.869317 | 0.002126 |
| Total.Unigene_20226 | BRE | 15.7624 | 0.273799 | 0.047461 |
| Total.Unigene_20240 | LYM4B | 24.2315 | 0.377484 | 0.030346 |
| Total.Unigene_21083 | HINT3 | 16.7388 | 0.273799 | 0.042793 |
| Total.Unigene_21631 | ARL1 | 13.7872 | 0.181626 | 0.031365 |
| Total.Unigene_22513 | LIP3 | 315.452 | 2.50502 | 0.014069 |
| Total.Unigene_22639 | ESTJ | 42.2591 | 0.391267 | 0.012608 |
| Total.Unigene_22722 | RNFT2 | 3.98637 | 0.069863 | 0.048167 |
| Total.Unigene_23325 | MIFH | 46.7316 | 1.02061 | 0.049996 |
| Total.Unigene_23433 | IVBI2 | 5408.14 | 15.972 | 0.002892 |
| Total.Unigene_23923 | PUR6 | 21.5598 | 0.452123 | 0.0474 |
| Total.Unigene_24048 | JHBP | 83.7704 | 0.854178 | 0.014434 |
| Total.Unigene_24804 | UDPE | 13.4761 | 0.176935 | 0.022617 |
| Total.Unigene_25098 | LAC5 | 104.49 | 0.748536 | 0.011133 |
| Total.Unigene_25100 | MLEC | 98.2386 | 0.457752 | 0.003702 |
| Total.Unigene_25144 | BACC2 | 103.079 | 0.748812 | 0.008288 |
| Total.Unigene_26273 | ELOV7 | 213.775 | 0.684358 | 0.00236 |
| Total.Unigene_26700 | DJC22 | 16.774 | 0.246938 | 0.026647 |
| Total.Unigene_26871 | ISCA1 | 16.135 | 0.344246 | 0.049532 |
| Total.Unigene_27169 | PKHA3 | 25.1962 | 0.290493 | 0.018273 |
| Total.Unigene_27407 | KDSR | 27.8821 | 0.587951 | 0.047352 |
| Total.Unigene_27961 | PSME3 | 8.74631 | 0.165074 | 0.043896 |
| Total.Unigene_28108 | PDI | 1579.32 | 13.4842 | 0.031937 |
| Total.Unigene_28275 | NT56 | 12.4382 | 0.078756 | 0.009647 |
| Total.Unigene_28519 | LEG1 | 13324.4 | 67.7713 | 0.016543 |
| Total.Unigene_28758 | TPIS | 60.1542 | 0.286171 | 0.004063 |
| Total.Unigene_28764 | LIN7C | 13.0761 | 0.23123 | 0.036814 |
| Total.Unigene_29196 | CT024 | 24.6721 | 0.337868 | 0.023235 |
| Total.Unigene_29604 | MMSB | 288.134 | 4.62315 | 0.036506 |
| Total.Unigene_29623 | DNK | 9.03294 | 0.125747 | 0.034011 |
| Total.Unigene_30109 | THIL | 46.9868 | 0.631701 | 0.024002 |
| Total.Unigene_30110 | THIL | 43.1766 | 0.665075 | 0.030346 |
| Total.Unigene_30845 | CU08 | 208.641 | 0.321748 | 0.000374 |
| Total.Unigene_31019 | PEB3 | 465.775 | 0.597061 | 0.000228 |
| Total.Unigene_31322 | CUE | 6.17612 | 0.051587 | 0.014987 |
| Total.Unigene_31409 | SYAC | 77.0469 | 0.248942 | 0.002159 |
| Total.Unigene_32166 | LIP3 | 61.3181 | 0.869871 | 0.028538 |
| Total.Unigene_32167 | LIP3 | 64.4132 | 0.954618 | 0.029612 |
| Total.Unigene_32208 | ARAE | 21.3614 | 0.117121 | 0.007507 |
| Total.Unigene_32447 | PNPO | 20.2074 | 0.348696 | 0.035389 |
| Total.Unigene_33066 | IVBIC | 402.194 | 1.9988 | 0.004519 |
| Total.Unigene_33067 | IVBIC | 440.746 | 1.97496 | 0.003621 |
| Total.Unigene_33086 | RIR2 | 78.6232 | 0.222629 | 0.001573 |
| Total.Unigene_33087 | RIR2 | 65.8088 | 0.231493 | 0.002415 |
| Total.Unigene_33585 | OV16 | 400.783 | 1.9916 | 0.004376 |
| Total.Unigene_33633 | MDH | 7.54396 | 0.132805 | 0.048476 |
| Total.Unigene_34130 | HGD | 56.7574 | 0.464581 | 0.01038 |
| Total.Unigene_34131 | HGD | 48.9762 | 0.958934 | 0.041586 |
| Total.Unigene_34377 | LIPI | 25.4676 | 0.270945 | 0.015997 |
| Total.Unigene_34510 | HXK1 | 3.85044 | 0.044069 | 0.024847 |
| Total.Unigene_34695 | ESTJ | 31.5268 | 0.083176 | 0.002313 |
| Total.Unigene_35118 | ADPGK | 110.883 | 1.31424 | 0.026284 |
| Total.Unigene_35323 | O16G2 | 7.10131 | 0.149559 | 0.047215 |
| Total.Unigene_35473 | RPIA | 16.6714 | 0.093496 | 0.007815 |
| Total.Unigene_35575 | BETA | 26.7603 | 0.336925 | 0.020408 |
| Total.Unigene_35576 | BETA | 28.9103 | 0.203272 | 0.007529 |
| Total.Unigene_36398 | TRET1 | 37.1751 | 0.192836 | 0.004435 |
| Total.Unigene_36399 | TRET1 | 33.2934 | 0.126321 | 0.002956 |
| Total.Unigene_36484 | CD034 | 24.4824 | 0.235278 | 0.027816 |
| Total.Unigene_36485 | CD034 | 24.6861 | 0.235792 | 0.027636 |
| Total.Unigene_36486 | CD034 | 24.6861 | 0.235792 | 0.027636 |
| Total.Unigene_36495 | CD034 | 46.6566 | 0.166358 | 0.005771 |
| Total.Unigene_36497 | CD034 | 24.4824 | 0.235278 | 0.027816 |
| Total.Unigene_37025 | FABG | 95.4328 | 0.937944 | 0.014434 |
| Total.Unigene_37026 | FABG | 93.1308 | 0.979798 | 0.01625 |
| Total.Unigene_37312 | TRET1 | 13.6402 | 0.173856 | 0.021309 |
| Total.Unigene_37595 | MTND | 45.738 | 0.489917 | 0.016255 |
| Total.Unigene_37596 | MTND | 49.4871 | 0.248113 | 0.00635 |
| Total.Unigene_38361 | KBL | 233.316 | 0.927055 | 0.004376 |
| Total.Unigene_38374 | SERA | 594.538 | 8.23076 | 0.042332 |
| Total.Unigene_38375 | SERA | 567.658 | 3.70176 | 0.011301 |
| Total.Unigene_38722 | TSPOA | 15.1087 | 0.223821 | 0.037049 |
| Total.Unigene_38752 | NLTP | 21.5748 | 0.318407 | 0.026724 |
| Total.Unigene_38753 | NLTP | 17.061 | 0.182331 | 0.017875 |
| Total.Unigene_38831 | ARSI | 290.522 | 3.12182 | 0.025006 |
| Total.Unigene_38985 | SERA | 106.418 | 1.31216 | 0.026206 |
| Total.Unigene_39314 | DHGL | 19.0064 | 0.309601 | 0.031814 |
| Total.Unigene_40774 | ACDSB | 31.3355 | 0.402592 | 0.020995 |
| Total.Unigene_40780 | ERDL7 | 12.2183 | 0.098019 | 0.011933 |
| Total.Unigene_40781 | ERDL7 | 11.7107 | 0.072147 | 0.009553 |
| Total.Unigene_40782 | ERDL7 | 12.725 | 0.102243 | 0.011933 |
| Total.Unigene_40783 | EDL15 | 13.7955 | 0.103612 | 0.010711 |
| Total.Unigene_40784 | ERDL7 | 12.2024 | 0.075299 | 0.009567 |
| Total.Unigene_40785 | EDL15 | 13.281 | 0.076321 | 0.008338 |
| Total.Unigene_40786 | ERDL7 | 12.8541 | 0.094423 | 0.012432 |
| Total.Unigene_40787 | ERDL7 | 12.2085 | 0.060883 | 0.018811 |
| Total.Unigene_41137 | CG010 | 34.2133 | 0.50493 | 0.027281 |
| Total.Unigene_41322 | L2AM | 217.788 | 2.88746 | 0.034011 |
| Total.Unigene_41660 | ARMET | 497.599 | 5.40582 | 0.022724 |
| Total.Unigene_42009 | COPD | 12.0352 | 0.246397 | 0.045051 |
| Total.Unigene_42039 | SV2C | 22.5565 | 0.314008 | 0.023964 |
| Total.Unigene_42170 | FKB1A | 11.6659 | 0.168372 | 0.029062 |
| Total.Unigene_42171 | FKB1A | 11.5328 | 0.168934 | 0.029925 |
| Total.Unigene_42186 | DHGL | 114.519 | 0.404353 | 0.00253 |
| Total.Unigene_42188 | BETA | 37.5374 | 0.114767 | 0.017595 |
| Total.Unigene_42210 | TBA1B | 50.1306 | 1.03036 | 0.045356 |
| Total.Unigene_42879 | DHGL | 40.9662 | 0.31608 | 0.009298 |
| Total.Unigene_43026 | DHB12 | 15.7354 | 0.244107 | 0.028876 |
| Total.Unigene_43027 | DHB12 | 14.8933 | 0.246637 | 0.032479 |
| Total.Unigene_43496 | HYEP | 53.8984 | 0.815599 | 0.029362 |
| Total.Unigene_44106 | TRABD | 5.55148 | 0.11341 | 0.046111 |
| Total.Unigene_44107 | TRABD | 5.49303 | 0.116144 | 0.048814 |
| Total.Unigene_44276 | ARF2 | 513.375 | 3.41876 | 0.00782 |
| Total.Unigene_44440 | DPM1 | 48.4612 | 0.695593 | 0.025606 |
| Total.Unigene_45077 | GOLP3 | 48.9053 | 0.82922 | 0.040106 |
| Total.Unigene_45335 | SAR1B | 69.116 | 0.644106 | 0.012507 |
| Total.Unigene_45348 | OCTL | 33.2704 | 0.501244 | 0.029117 |
| Total.Unigene_45410 | IMPI | 18.4798 | 0.297122 | 0.034269 |
| Total.Unigene_45411 | IMPI | 21.5811 | 0.323631 | 0.030565 |
| Total.Unigene_45412 | IMPI | 28.2063 | 0.478408 | 0.036954 |
| Total.Unigene_45413 | IMPI | 108.955 | 1.03925 | 0.017509 |
| Total.Unigene_45414 | IMPI | 119.416 | 1.0695 | 0.015619 |
| Total.Unigene_45421 | PAI1 | 860.016 | 14.5745 | 0.04956 |
| Total.Unigene_45639 | G6PD | 12.8184 | 0.079875 | 0.009364 |
| Total.Unigene_45935 | YELL | 82.0161 | 0.366186 | 0.00363 |
| Total.Unigene_46823 | CLOCK | 3.55361 | 0.039745 | 0.023858 |
| Total.Unigene_46824 | CLOCK | 3.65399 | 0.039957 | 0.022917 |
| Total.Unigene_46912 | ADK | 12.6888 | 0.20167 | 0.031397 |
| Total.Unigene_47076 | ERD6 | 7.86441 | 0.05048 | 0.013502 |
| Total.Unigene_47077 | ERD6 | 7.65335 | 0.079568 | 0.028538 |
| Total.Unigene_47081 | VPS25 | 11.1594 | 0.152644 | 0.033237 |
| Total.Unigene_47270 | DHGL | 45.3764 | 0.825708 | 0.041245 |
| Total.Unigene_47272 | DHGL | 50.0135 | 1.00838 | 0.043896 |
| Total.Unigene_47629 | TRE12 | 9.07994 | 0.162137 | 0.036007 |
| Total.Unigene_47630 | TRE12 | 10.886 | 0.197848 | 0.036954 |
| Total.Unigene_47631 | TRE12 | 11.3688 | 0.179567 | 0.02993 |
| Total.Unigene_47632 | TRE12 | 14.6132 | 0.235112 | 0.03088 |
| Total.Unigene_47659 | UDPE | 7.45165 | 0.062775 | 0.015238 |
| Total.Unigene_48093 | ELOV1 | 81.6645 | 0.28122 | 0.003326 |
| Total.Unigene_48142 | CIN | 5.09977 | 0.088355 | 0.038791 |
| Total.Unigene_48143 | CIN | 5.44071 | 0.09197 | 0.037633 |
| Total.Unigene_48214 | S36A4 | 8.12664 | 0.147698 | 0.036954 |
| Total.Unigene_48311 | AFMID | 7.64196 | 0.117417 | 0.033898 |
| Total.Unigene_48312 | AFMID | 7.74307 | 0.118971 | 0.033898 |
| Total.Unigene_48386 | PPB | 65.9599 | 0.724915 | 0.018736 |
| Total.Unigene_48826 | DHGL | 12.4225 | 0.03664 | 0.016698 |
| Total.Unigene_48827 | DHGL | 12.9432 | 0.038348 | 0.016821 |
| Total.Unigene_48838 | DHGL | 43.7023 | 0.464729 | 0.016505 |
| Total.Unigene_49332 | BHA15 | 17.7589 | 0.291091 | 0.032093 |
| Total.Unigene_49364 | AQP | 11.7102 | 0.03666 | 0.018067 |
| Total.Unigene_49365 | AQP | 12.6692 | 0.042802 | 0.019822 |
| Total.Unigene_49377 | XDH | 45.777 | 0.257362 | 0.005714 |
| Total.Unigene_49378 | XDH | 47.2619 | 0.153991 | 0.002159 |
| Total.Unigene_49700 | TRE12 | 138.347 | 0.292247 | 0.000878 |
| Total.Unigene_49701 | TRE12 | 132.659 | 0.165433 | 0.00021 |
| Total.Unigene_49802 | AADAT | 30.4924 | 0.213689 | 0.007595 |
| Total.Unigene_49803 | AADAT | 26.0703 | 0.146998 | 0.005072 |
| Total.Unigene_49804 | AADAT | 26.4316 | 0.148842 | 0.005072 |
| Total.Unigene_49834 | SV2B | 24.1348 | 0.465202 | 0.041634 |
| Total.Unigene_50358 | AP2S1 | 14.3825 | 0.112823 | 0.023291 |
| Total.Unigene_50359 | AP2S1 | 16.2838 | 0.147976 | 0.029354 |
| Total.Unigene_50825 | PERO | 311.013 | 4.8655 | 0.048694 |
| Total.Unigene_51476 | TRET1 | 19.8568 | 0.221875 | 0.016891 |
| Total.Unigene_53012 | TRET1 | 5.10158 | 0.047038 | 0.015476 |
| Total.Unigene_53013 | TRET1 | 6.36011 | 0.059957 | 0.01409 |
| Total.Unigene_53014 | TRET1 | 5.34168 | 0.069962 | 0.024019 |
| Total.Unigene_53016 | TRET1 | 4.937 | 0.070788 | 0.028134 |
| Total.Unigene_53017 | TRET1 | 5.96919 | 0.085733 | 0.026724 |
| Total.Unigene_53019 | TRET1 | 6.1488 | 0.024915 | 0.018812 |
| Total.Unigene_9455 | PP2B3 | 44.0267 | 0.777228 | 0.042134 |

| Gene_Id | Gene Name | Prepupae（FPKM） | Pupae (FPKM) | q value |
| --- | --- | --- | --- | --- |
| Up-regulated |  |  |  |  |
| Total.Unigene_10478 | LUCI | 0.187907 | 15.1134 | 0.049572 |
| Total.Unigene_11461 | RTXE | 0.145037 | 28.3587 | 0.013618 |
| Total.Unigene_12964 | PELET | 0.387413 | 23.4771 | 0.033938 |
| Total.Unigene_15246 | BOP1 | 0.460518 | 24.2672 | 0.039954 |
| Total.Unigene_15686 | CORO7 | 0.131152 | 14.004 | 0.033442 |
| Total.Unigene_16560 | MOV10 | 0.29305 | 18.9982 | 0.028538 |
| Total.Unigene_16572 | YRD6 | 0.135176 | 35.8658 | 0.002678 |
| Total.Unigene_18135 | CI030 | 0.217491 | 20.6811 | 0.018767 |
| Total.Unigene_18634 | MSH6 | 0.326599 | 26.4948 | 0.022684 |
| Total.Unigene_19683 | MYO9A | 0.597179 | 32.5728 | 0.038006 |
| Total.Unigene_21597 | AIFM1 | 0.128473 | 8.6703 | 0.035466 |
| Total.Unigene_22242 | RTXE | 0.271977 | 24.5152 | 0.041765 |
| Total.Unigene_22359 | POL | 0.279598 | 43.6836 | 0.006525 |
| Total.Unigene_22648 | APOD | 0.100623 | 127.676 | 0.000557 |
| Total.Unigene_22901 | POLX | 0.224549 | 10.5872 | 0.0474 |
| Total.Unigene_24732 | TCRG1 | 0.155221 | 12.4485 | 0.049874 |
| Total.Unigene_25042 | TRX | 2.29939 | 177.588 | 0.035007 |
| Total.Unigene_25125 | TC3A | 0.151312 | 26.1759 | 0.006304 |
| Total.Unigene_26290 | CCDCX | 0.229793 | 33.4062 | 0.007503 |
| Total.Unigene_26291 | CCDCX | 0.279655 | 34.7361 | 0.009995 |
| Total.Unigene_26618 | H4 | 1.61589 | 105.411 | 0.035007 |
| Total.Unigene_26710 | DSCL | 0.044018 | 2.50257 | 0.037064 |
| Total.Unigene_26711 | DSCL | 0.048615 | 2.50735 | 0.042152 |
| Total.Unigene_26712 | DSCL | 0.044818 | 2.46137 | 0.038754 |
| Total.Unigene_26713 | DSCL | 0.049503 | 2.46604 | 0.044953 |
| Total.Unigene_26714 | DSCL | 0.018094 | 3.08676 | 0.037909 |
| Total.Unigene_26715 | DSCL | 0.024636 | 3.09743 | 0.028988 |
| Total.Unigene_26716 | DSCL | 0.018577 | 3.04187 | 0.039873 |
| Total.Unigene_26717 | DSCL | 0.025298 | 3.05253 | 0.030911 |
| Total.Unigene_26736 | ZFP26 | 0.806906 | 53.7098 | 0.02812 |
| Total.Unigene_26931 | PCX | 0.411936 | 32.1935 | 0.023712 |
| Total.Unigene_26999 | CLVS1 | 0.132199 | 7.63844 | 0.045035 |
| Total.Unigene_27584 | MYNN | 0.508243 | 42.9923 | 0.018767 |
| Total.Unigene_27633 | AKC1H | 0.12225 | 25.429 | 0.012523 |
| Total.Unigene_27713 | GSTT1 | 0.721954 | 40.046 | 0.036765 |
| Total.Unigene_28125 | 41 | 0.167942 | 14.5556 | 0.017722 |
| Total.Unigene_29116 | ACO11 | 0.081707 | 13.5588 | 0.008288 |
| Total.Unigene_29117 | ACO11 | 0.027358 | 13.7715 | 0.025638 |
| Total.Unigene_29124 | RTXE | 0.331221 | 19.7312 | 0.033045 |
| Total.Unigene_29651 | PERQ1 | 0.835788 | 98.8347 | 0.013648 |
| Total.Unigene_29652 | PERQ1 | 0.73962 | 89.3033 | 0.012737 |
| Total.Unigene_30009 | ZN323 | 0.243109 | 35.6173 | 0.007354 |
| Total.Unigene_30010 | ZN323 | 0.289898 | 41.2413 | 0.00782 |
| Total.Unigene_30439 | ASM | 0.084739 | 6.18396 | 0.027281 |
| Total.Unigene_30440 | ASM | 0.088077 | 4.99242 | 0.039873 |
| Total.Unigene_30704 | ZG20 | 0.335505 | 56.5282 | 0.005495 |
| Total.Unigene_31236 | ZG26 | 0.421232 | 78.4197 | 0.004655 |
| Total.Unigene_31393 | TCRG1 | 0.386867 | 43.7885 | 0.011854 |
| Total.Unigene_31395 | TCRG1 | 0.496608 | 60.0678 | 0.010792 |
| Total.Unigene_31396 | TCRG1 | 0.471801 | 61.2814 | 0.009364 |
| Total.Unigene_32192 | ESMC | 0.274445 | 15.128 | 0.036935 |
| Total.Unigene_32569 | DCR1 | 0.649557 | 48.3636 | 0.025417 |
| Total.Unigene_32806 | H1B | 0.146129 | 13.1266 | 0.018415 |
| Total.Unigene_32807 | H1B | 0.12608 | 12.3831 | 0.0171 |
| Total.Unigene_32808 | H1B | 0.162153 | 13.017 | 0.021926 |
| Total.Unigene_32809 | H1B | 0.140103 | 12.1892 | 0.020371 |
| Total.Unigene_32810 | H1B | 0.219158 | 18.0068 | 0.022149 |
| Total.Unigene_32811 | H1B | 0.186627 | 16.8478 | 0.020565 |
| Total.Unigene_33334 | C3390 | 0.15846 | 12.0216 | 0.025502 |
| Total.Unigene_33335 | C3390 | 0.16128 | 15.9786 | 0.019241 |
| Total.Unigene_33661 | AZI1 | 0.062565 | 10.1076 | 0.018179 |
| Total.Unigene_33662 | AZI1 | 0.082524 | 8.64008 | 0.034269 |
| Total.Unigene_33874 | RTXE | 0.695727 | 44.7934 | 0.029612 |
| Total.Unigene_35164 | AFAD | 0.584543 | 57.5525 | 0.014886 |
| Total.Unigene_35165 | AFAD | 0.630956 | 59.4973 | 0.015997 |
| Total.Unigene_35672 | MMP14 | 0.193996 | 242.038 | 8.79E-05 |
| Total.Unigene_35673 | MMP14 | 0.294837 | 368.772 | 8.79E-05 |
| Total.Unigene_35674 | MMP14 | 0.282266 | 346.773 | 8.79E-05 |
| Total.Unigene_35675 | MMP14 | 0.31536 | 369.385 | 0.000102 |
| Total.Unigene_35676 | MMP14 | 0.293239 | 366.876 | 8.79E-05 |
| Total.Unigene_36407 | DDX52 | 0.542584 | 32.128 | 0.035007 |
| Total.Unigene_36466 | WDR65 | 0.219178 | 44.5777 | 0.004227 |
| Total.Unigene_36467 | WDR65 | 0.314036 | 67.9351 | 0.003591 |
| Total.Unigene_38151 | CHIA | 1.38591 | 110.526 | 0.026206 |
| Total.Unigene_38152 | CHIA | 1.33854 | 113.543 | 0.023077 |
| Total.Unigene_38754 | YI31B | 0.269371 | 14.5682 | 0.049984 |
| Total.Unigene_39284 | VPS72 | 0.974502 | 73.6763 | 0.024494 |
| Total.Unigene_39285 | VPS72 | 0.651892 | 34.7626 | 0.038597 |
| Total.Unigene_39595 | HNRL1 | 1.38561 | 117.509 | 0.027644 |
| Total.Unigene_39596 | HNRL1 | 1.29041 | 136.9 | 0.018421 |
| Total.Unigene_39597 | HNRL1 | 1.42616 | 155.751 | 0.01764 |
| Total.Unigene_39868 | ARIP4 | 0.986477 | 70.6625 | 0.035007 |
| Total.Unigene_39869 | ARIP4 | 0.697026 | 52.1633 | 0.026943 |
| Total.Unigene_39887 | Y9776 | 0.367843 | 28.2035 | 0.022113 |
| Total.Unigene_39888 | Y9776 | 0.338929 | 25.1735 | 0.023119 |
| Total.Unigene_39889 | Y9776 | 0.313857 | 33.3249 | 0.012682 |
| Total.Unigene_39890 | Y9776 | 0.278865 | 29.7726 | 0.012507 |
| Total.Unigene_40052 | CHS2 | 0.414842 | 50.969 | 0.010922 |
| Total.Unigene_41732 | RTF22 | 0.127412 | 12.6624 | 0.015615 |
| Total.Unigene_42246 | ZN642 | 0.379429 | 21.6423 | 0.035364 |
| Total.Unigene_42247 | ZN177 | 0.281566 | 24.288 | 0.017875 |
| Total.Unigene_43602 | CB029 | 0.360384 | 19.2048 | 0.038597 |
| Total.Unigene_45260 | YMD2 | 0.996001 | 49.7224 | 0.042793 |
| Total.Unigene_45263 | YMD3 | 0.221751 | 46.4336 | 0.012432 |
| Total.Unigene_45929 | PPID | 0.531529 | 42.3257 | 0.022814 |
| Total.Unigene_47020 | RSBNL | 0.41489 | 22.1114 | 0.042793 |
| Total.Unigene_47021 | RSBNL | 0.418184 | 22.2869 | 0.042793 |
| Total.Unigene_47022 | RSBNL | 0.404944 | 22.3343 | 0.040272 |
| Total.Unigene_47023 | RSBNL | 0.34463 | 21.7124 | 0.031785 |
| Total.Unigene_47434 | ENTK | 0.318618 | 15.4642 | 0.045725 |
| Total.Unigene_48060 | POL4 | 0.553743 | 56.9158 | 0.014987 |
| Total.Unigene_48061 | POL4 | 0.35507 | 28.0659 | 0.020296 |
| Total.Unigene_48181 | RBCC1 | 1.29399 | 75.8667 | 0.040102 |
| Total.Unigene_48496 | SCLXB | 0.238636 | 14.2152 | 0.03334 |
| Total.Unigene_48497 | SCLXB | 0.239761 | 14.21 | 0.033483 |
| Total.Unigene_48498 | SCLXB | 0.231113 | 14.0621 | 0.032373 |
| Total.Unigene_48499 | SCLXB | 0.232234 | 14.056 | 0.032479 |
| Total.Unigene_4889 | RNO | 0.42545 | 64.0552 | 0.008288 |
| Total.Unigene_49057 | ZNF83 | 0.542005 | 28.8674 | 0.042668 |
| Total.Unigene_49058 | ZNF83 | 0.547657 | 29.1122 | 0.042761 |
| Total.Unigene_50796 | RTF1 | 0.128722 | 11.5014 | 0.04232 |
| Total.Unigene_50798 | RTF1 | 0.134043 | 14.0216 | 0.034286 |
| Total.Unigene_52909 | MDN1 | 0.133485 | 24.9977 | 0.004655 |
| Total.Unigene_52910 | MDN1 | 0.136273 | 25.5371 | 0.004655 |
| Total.Unigene_52911 | MDN1 | 0.086392 | 33.0534 | 0.001426 |
| Total.Unigene_52912 | MDN1 | 0.088985 | 34.07 | 0.001426 |
| Total.Unigene_53706 | PIF1 | 0.260454 | 15.563 | 0.035689 |
| Total.Unigene_53707 | PIF1 | 0.265125 | 15.6745 | 0.036496 |
| Total.Unigene_53708 | PIF1 | 0.258963 | 15.6595 | 0.035071 |
| Total.Unigene_53709 | PIF1 | 0.263658 | 15.7656 | 0.035689 |
| Total.Unigene_53710 | PIF1 | 0.282748 | 16.5984 | 0.036884 |
| Total.Unigene_53711 | PIF1 | 0.278844 | 16.6271 | 0.035704 |
| Total.Unigene_53712 | PIF1 | 0.287903 | 16.72 | 0.037265 |
| Total.Unigene_53713 | PIF1 | 0.281328 | 16.7081 | 0.036027 |
| Total.Unigene_53714 | PIF1 | 0.283979 | 16.7502 | 0.03662 |
| Total.Unigene_53715 | PIF1 | 0.277391 | 16.7341 | 0.035222 |
| Total.Unigene_53716 | PIF1 | 0.286514 | 16.8291 | 0.036827 |
| Total.Unigene_53717 | PIF1 | 0.282556 | 16.8589 | 0.035704 |
| Total.Unigene_53718 | RTBS | 0.310971 | 19.8171 | 0.032479 |
| Total.Unigene_53719 | RTBS | 0.344928 | 21.5652 | 0.03334 |
| Total.Unigene_53720 | RTBS | 0.339911 | 21.6136 | 0.032479 |
| Total.Unigene_53811 | RRP5 | 0.972084 | 67.1598 | 0.033339 |
| Total.Unigene_54256 | SCAPE | 0.931455 | 68.0012 | 0.033898 |
| Total.Unigene_54259 | SCAPE | 1.15288 | 114.462 | 0.018223 |
| Total.Unigene_55648 | RTBS | 0.172973 | 12.9528 | 0.026182 |
| Total.Unigene_8937 | POL4 | 0.350301 | 20.8881 | 0.033531 |
| Total.Unigene_9383 | DJC16 | 0.203563 | 10.3034 | 0.048278 |
| Down-regulated |  |  |  |  |
| Total.Unigene_12603 | PERO | 12.0946 | 0.152471 | 0.029062 |
| Total.Unigene_16903 | GLCM | 60.3561 | 0.436681 | 0.007838 |
| Total.Unigene_17941 | DPGN | 27486.8 | 82.1916 | 0.026284 |
| Total.Unigene_19384 | ESTF | 5.8475 | 0.079499 | 0.033004 |
| Total.Unigene_19976 | UD11 | 19.213 | 0.296034 | 0.03879 |
| Total.Unigene_20048 | TM9S4 | 24.5776 | 0.416363 | 0.035034 |
| Total.Unigene_20150 | PRS42 | 107.776 | 0.748683 | 0.007507 |
| Total.Unigene_20151 | TM11F | 90.7418 | 0.869317 | 0.013034 |
| Total.Unigene_20226 | BRE | 20.3367 | 0.273799 | 0.032526 |
| Total.Unigene_20329 | CP18A | 534.658 | 1.35524 | 0.002501 |
| Total.Unigene_20474 | CYB5 | 38.917 | 0.203649 | 0.004655 |
| Total.Unigene_21631 | ARL1 | 11.7877 | 0.181626 | 0.03879 |
| Total.Unigene_21896 | AEDO | 4.70454 | 0.08364 | 0.049294 |
| Total.Unigene_22173 | ODB2 | 27.8251 | 0.363159 | 0.021444 |
| Total.Unigene_22722 | RNFT2 | 4.83648 | 0.069863 | 0.035689 |
| Total.Unigene_24020 | LIP3 | 139.125 | 1.72978 | 0.024635 |
| Total.Unigene_24804 | UDPE | 26.2839 | 0.176935 | 0.0074 |
| Total.Unigene_24826 | DPGN | 2968.7 | 23.9462 | 0.045055 |
| Total.Unigene_25098 | LAC5 | 181.454 | 0.748536 | 0.004459 |
| Total.Unigene_25144 | BACC2 | 34.9912 | 0.748812 | 0.049034 |
| Total.Unigene_25806 | MK14B | 6.695 | 0.130029 | 0.049996 |
| Total.Unigene_26314 | RAB7A | 105.733 | 2.01589 | 0.047352 |
| Total.Unigene_26700 | DJC22 | 25.1676 | 0.246938 | 0.013363 |
| Total.Unigene_26871 | ISCA1 | 18.3701 | 0.344246 | 0.039798 |
| Total.Unigene_27050 | S41A1 | 19.3616 | 0.296131 | 0.028538 |
| Total.Unigene_27051 | S41A1 | 18.784 | 0.299788 | 0.030861 |
| Total.Unigene_27169 | PKHA3 | 24.6665 | 0.290493 | 0.018866 |
| Total.Unigene_28275 | NT56 | 8.0078 | 0.078756 | 0.019395 |
| Total.Unigene_28277 | TRET1 | 9.80003 | 0.025028 | 0.013648 |
| Total.Unigene_28278 | TRET1 | 7.9321 | 0.038326 | 0.032241 |
| Total.Unigene_28764 | LIN7C | 12.7561 | 0.23123 | 0.037811 |
| Total.Unigene_29224 | NPRL2 | 7.51354 | 0.13024 | 0.0474 |
| Total.Unigene_29260 | CBR1 | 67.2105 | 1.20261 | 0.036506 |
| Total.Unigene_29638 | LIP1 | 8.9064 | 0.09428 | 0.02162 |
| Total.Unigene_29639 | LIP1 | 8.50179 | 0.095078 | 0.023858 |
| Total.Unigene_30253 | S35A3 | 7.44544 | 0.148915 | 0.048404 |
| Total.Unigene_30920 | GAGXE | 98.7546 | 1.66791 | 0.045712 |
| Total.Unigene_30963 | DHGL | 52.0654 | 0.396341 | 0.009126 |
| Total.Unigene_31322 | CUE | 4.65004 | 0.051587 | 0.023498 |
| Total.Unigene_31375 | ESTJ | 234.356 | 1.19921 | 0.005769 |
| Total.Unigene_31409 | SYAC | 69.5632 | 0.248942 | 0.002527 |
| Total.Unigene_31856 | A1A1A | 8.2352 | 0.156706 | 0.047055 |
| Total.Unigene_31857 | A1A1A | 9.43044 | 0.161201 | 0.039022 |
| Total.Unigene_31972 | DJC25 | 157.393 | 1.40748 | 0.014434 |
| Total.Unigene_32076 | KAPC | 17.3283 | 0.246037 | 0.024847 |
| Total.Unigene_32166 | LIP3 | 126.655 | 0.869871 | 0.009006 |
| Total.Unigene_32167 | LIP3 | 157.261 | 0.954618 | 0.006957 |
| Total.Unigene_32208 | ARAE | 11.1486 | 0.117121 | 0.02134 |
| Total.Unigene_32775 | MIOX | 12.5334 | 0.124145 | 0.016604 |
| Total.Unigene_32776 | MIOX | 16.6708 | 0.148428 | 0.013823 |
| Total.Unigene_33066 | IVBIC | 255.679 | 1.9988 | 0.009924 |
| Total.Unigene_33067 | IVBIC | 452.634 | 1.97496 | 0.003463 |
| Total.Unigene_33206 | HSBP1 | 57.0311 | 1.15736 | 0.043896 |
| Total.Unigene_33455 | IP3KB | 10.9513 | 0.136405 | 0.020408 |
| Total.Unigene_33456 | IP3KB | 12.4437 | 0.151923 | 0.020003 |
| Total.Unigene_34695 | ESTJ | 25.3115 | 0.083176 | 0.003004 |
| Total.Unigene_35118 | ADPGK | 77.7137 | 1.31424 | 0.043896 |
| Total.Unigene_35190 | TRET1 | 109.471 | 0.672454 | 0.006868 |
| Total.Unigene_35451 | CRYL1 | 86.9343 | 1.23899 | 0.028416 |
| Total.Unigene_35473 | RPIA | 6.20284 | 0.093496 | 0.037741 |
| Total.Unigene_36084 | VLDLR | 26.144 | 0.460754 | 0.038017 |
| Total.Unigene_36398 | TRET1 | 86.3557 | 0.192836 | 0.000892 |
| Total.Unigene_36399 | TRET1 | 76.4881 | 0.126321 | 0.000546 |
| Total.Unigene_36484 | CD034 | 35.3115 | 0.235278 | 0.015815 |
| Total.Unigene_36485 | CD034 | 35.4949 | 0.235792 | 0.015761 |
| Total.Unigene_36486 | CD034 | 35.7074 | 0.235792 | 0.015625 |
| Total.Unigene_36495 | CD034 | 70.4794 | 0.166358 | 0.002956 |
| Total.Unigene_36497 | CD034 | 35.3115 | 0.235278 | 0.015815 |
| Total.Unigene_36708 | SOX21 | 3.60546 | 0.056207 | 0.0368 |
| Total.Unigene_36709 | SOX21 | 3.5867 | 0.056551 | 0.037179 |
| Total.Unigene_37187 | S39AE | 17.2535 | 0.279019 | 0.031365 |
| Total.Unigene_37750 | HR3 | 5.32075 | 0.074529 | 0.027816 |
| Total.Unigene_37751 | HR3 | 5.453 | 0.050581 | 0.017875 |
| Total.Unigene_38178 | XYLS | 56.3474 | 0.454522 | 0.011933 |
| Total.Unigene_38361 | KBL | 68.1893 | 0.927055 | 0.033004 |
| Total.Unigene_38373 | ACH2 | 407.467 | 2.95868 | 0.014535 |
| Total.Unigene_38689 | RDHE2 | 20.5576 | 0.316274 | 0.028538 |
| Total.Unigene_38690 | RDHE2 | 20.7522 | 0.319268 | 0.028538 |
| Total.Unigene_40780 | ERDL7 | 6.86851 | 0.098019 | 0.030346 |
| Total.Unigene_40781 | ERDL7 | 6.58476 | 0.072147 | 0.023792 |
| Total.Unigene_40782 | ERDL7 | 7.16446 | 0.102243 | 0.030346 |
| Total.Unigene_40783 | EDL15 | 7.67095 | 0.103612 | 0.027783 |
| Total.Unigene_40784 | ERDL7 | 6.87245 | 0.075299 | 0.023792 |
| Total.Unigene_40785 | EDL15 | 7.38215 | 0.076321 | 0.021432 |
| Total.Unigene_40786 | ERDL7 | 6.7528 | 0.094423 | 0.034493 |
| Total.Unigene_40787 | ERDL7 | 6.38365 | 0.060883 | 0.046111 |
| Total.Unigene_40898 | TRET1 | 9.822 | 0.116414 | 0.026487 |
| Total.Unigene_41322 | L2AM | 747.313 | 2.88746 | 0.005165 |
| Total.Unigene_41682 | DPGN | 11344.9 | 52.1145 | 0.022149 |
| Total.Unigene_41796 | C1GLT | 9.42988 | 0.19767 | 0.046437 |
| Total.Unigene_41797 | C1GLT | 9.15945 | 0.050138 | 0.013639 |
| Total.Unigene_41940 | GALT9 | 12.2725 | 0.172777 | 0.024693 |
| Total.Unigene_41941 | GALT9 | 12.0193 | 0.172943 | 0.025638 |
| Total.Unigene_42039 | SV2C | 31.7769 | 0.314008 | 0.013474 |
| Total.Unigene_42124 | DPGN | 1022.33 | 5.84843 | 0.012273 |
| Total.Unigene_42176 | DHGL | 131.117 | 2.24082 | 0.049962 |
| Total.Unigene_42186 | DHGL | 41.1936 | 0.404353 | 0.013831 |
| Total.Unigene_42188 | BETA | 18.9524 | 0.114767 | 0.041545 |
| Total.Unigene_43111 | SCP | 51.4498 | 0.958386 | 0.041586 |
| Total.Unigene_43112 | SCP | 50.4112 | 0.761033 | 0.029215 |
| Total.Unigene_43463 | PERO | 32.8834 | 0.072849 | 0.003208 |
| Total.Unigene_43464 | PERO | 30.1894 | 0.078502 | 0.004304 |
| Total.Unigene_43480 | SO5A1 | 3.60022 | 0.059376 | 0.043544 |
| Total.Unigene_43707 | PCKG | 52.819 | 0.434604 | 0.011236 |
| Total.Unigene_43708 | PCKG | 50.2068 | 0.4463 | 0.012531 |
| Total.Unigene_43786 | SIK2 | 19.8125 | 0.19971 | 0.015071 |
| Total.Unigene_43885 | GALE | 40.71 | 0.429667 | 0.015476 |
| Total.Unigene_43886 | GALE | 53.8397 | 0.616336 | 0.018028 |
| Total.Unigene_43925 | PDE6D | 10.4426 | 0.067668 | 0.045712 |
| Total.Unigene_43926 | PDE6D | 14.7201 | 0.28351 | 0.045712 |
| Total.Unigene_44106 | TRABD | 7.54443 | 0.11341 | 0.028528 |
| Total.Unigene_44107 | TRABD | 7.49601 | 0.116144 | 0.030045 |
| Total.Unigene_44152 | FA46A | 7.24923 | 0.119013 | 0.033045 |
| Total.Unigene_44153 | FA46A | 7.30076 | 0.119859 | 0.033045 |
| Total.Unigene_44276 | ARF2 | 584.597 | 3.41876 | 0.006264 |
| Total.Unigene_44440 | DPM1 | 53.6722 | 0.695593 | 0.021217 |
| Total.Unigene_44853 | CECR1 | 19.8745 | 0.321405 | 0.031365 |
| Total.Unigene_45077 | GOLP3 | 67.2549 | 0.82922 | 0.025135 |
| Total.Unigene_45335 | SAR1B | 120.388 | 0.644106 | 0.004655 |
| Total.Unigene_45410 | IMPI | 155.699 | 0.297122 | 0.000893 |
| Total.Unigene_45411 | IMPI | 183.11 | 0.323631 | 0.000789 |
| Total.Unigene_45412 | IMPI | 248.919 | 0.478408 | 0.000893 |
| Total.Unigene_45413 | IMPI | 352.303 | 1.03925 | 0.002827 |
| Total.Unigene_45414 | IMPI | 384.078 | 1.0695 | 0.00253 |
| Total.Unigene_45639 | G6PD | 20.2176 | 0.079875 | 0.004337 |
| Total.Unigene_45935 | YELL | 52.1492 | 0.366186 | 0.007911 |
| Total.Unigene_46152 | PERO | 935.032 | 6.54486 | 0.018767 |
| Total.Unigene_46153 | PERO | 1379.21 | 2.54873 | 0.001393 |
| Total.Unigene_46154 | PERO | 4882.88 | 5.29331 | 0.000411 |
| Total.Unigene_46823 | CLOCK | 16.0359 | 0.039745 | 0.002126 |
| Total.Unigene_46824 | CLOCK | 16.4095 | 0.039957 | 0.002063 |
| Total.Unigene_47362 | MOT14 | 51.9698 | 0.677589 | 0.028073 |
| Total.Unigene_47363 | MOT14 | 52.7714 | 0.683754 | 0.02786 |
| Total.Unigene_47603 | T184C | 6.18073 | 0.069683 | 0.024125 |
| Total.Unigene_47659 | UDPE | 71.7203 | 0.062775 | 0.000163 |
| Total.Unigene_48002 | MED4 | 5.22359 | 0.090546 | 0.0474 |
| Total.Unigene_48142 | CIN | 4.59873 | 0.088355 | 0.046437 |
| Total.Unigene_48143 | CIN | 4.78688 | 0.09197 | 0.046437 |
| Total.Unigene_48214 | S36A4 | 34.1184 | 0.147698 | 0.003121 |
| Total.Unigene_48309 | AFMID | 16.754 | 0.301794 | 0.037064 |
| Total.Unigene_48310 | AFMID | 16.7589 | 0.30452 | 0.037439 |
| Total.Unigene_48311 | AFMID | 12.8861 | 0.117417 | 0.014434 |
| Total.Unigene_48312 | AFMID | 12.8421 | 0.118971 | 0.014874 |
| Total.Unigene_48316 | AFMID | 20.5587 | 0.283392 | 0.042859 |
| Total.Unigene_48480 | SPIN | 11.6182 | 0.135374 | 0.018012 |
| Total.Unigene_48481 | SPIN | 13.0936 | 0.149065 | 0.017386 |
| Total.Unigene_48482 | SPIN | 11.4166 | 0.123033 | 0.015862 |
| Total.Unigene_48483 | SPNS1 | 9.94138 | 0.111149 | 0.018255 |
| Total.Unigene_48484 | SPIN | 11.0679 | 0.196843 | 0.035765 |
| Total.Unigene_48485 | SPIN | 10.6164 | 0.166469 | 0.030189 |
| Total.Unigene_48486 | SPIN | 12.7089 | 0.136937 | 0.017453 |
| Total.Unigene_48487 | SPIN | 10.0992 | 0.150771 | 0.029419 |
| Total.Unigene_48488 | SPIN | 13.7825 | 0.067476 | 0.015625 |
| Total.Unigene_48489 | SPIN | 11.3719 | 0.068713 | 0.02069 |
| Total.Unigene_48718 | BGAL | 13.6113 | 0.252509 | 0.038017 |
| Total.Unigene_48826 | DHGL | 18.4556 | 0.03664 | 0.009924 |
| Total.Unigene_48827 | DHGL | 19.1778 | 0.038348 | 0.009995 |
| Total.Unigene_48838 | DHGL | 122.565 | 0.464729 | 0.002932 |
| Total.Unigene_49054 | TM104 | 22.0656 | 0.303334 | 0.023646 |
| Total.Unigene_49055 | TM104 | 22.128 | 0.345264 | 0.029395 |
| Total.Unigene_49137 | GFPT1 | 41.2029 | 0.375284 | 0.012113 |
| Total.Unigene_49139 | GFPT2 | 15.4897 | 0.202641 | 0.021405 |
| Total.Unigene_49326 | BHA15 | 25.8468 | 0.499878 | 0.04323 |
| Total.Unigene_49327 | BHA15 | 29.2817 | 0.358961 | 0.019548 |
| Total.Unigene_49332 | BHA15 | 33.7172 | 0.291091 | 0.011047 |
| Total.Unigene_49364 | AQP | 19.6244 | 0.03666 | 0.009048 |
| Total.Unigene_49365 | AQP | 20.8136 | 0.042802 | 0.010428 |
| Total.Unigene_49377 | XDH | 162.033 | 0.257362 | 0.000546 |
| Total.Unigene_49378 | XDH | 155.814 | 0.153991 | 0.000108 |
| Total.Unigene_49552 | XDH | 35.2641 | 0.629521 | 0.043876 |
| Total.Unigene_49553 | ALDO1 | 25.2707 | 0.230465 | 0.012197 |
| Total.Unigene_49555 | ADO | 17.6113 | 0.145803 | 0.011796 |
| Total.Unigene_49672 | TM110 | 32.7257 | 0.321854 | 0.015264 |
| Total.Unigene_49676 | S22A1 | 37.7538 | 0.398433 | 0.015615 |
| Total.Unigene_49700 | TRE12 | 72.6444 | 0.292247 | 0.002956 |
| Total.Unigene_49701 | TRE12 | 77.633 | 0.165433 | 0.000829 |
| Total.Unigene_49802 | AADAT | 25.473 | 0.213689 | 0.010532 |
| Total.Unigene_49803 | AADAT | 23.8116 | 0.146998 | 0.006148 |
| Total.Unigene_49804 | AADAT | 24.159 | 0.148842 | 0.006123 |
| Total.Unigene_50097 | RNOY | 12.5477 | 0.068147 | 0.036701 |
| Total.Unigene_50099 | RNOY | 12.5943 | 0.071773 | 0.038405 |
| Total.Unigene_50358 | AP2S1 | 10.414 | 0.112823 | 0.037352 |
| Total.Unigene_50359 | AP2S1 | 13.801 | 0.147976 | 0.036954 |
| Total.Unigene_50526 | COPT1 | 576.676 | 5.31159 | 0.017722 |
| Total.Unigene_50662 | MOC2B | 16.9479 | 0.117945 | 0.007815 |
| Total.Unigene_50663 | MOC2B | 17.7523 | 0.137564 | 0.00929 |
| Total.Unigene_50664 | MOC2B | 18.6151 | 0.12503 | 0.007434 |
| Total.Unigene_50665 | MOC2B | 16.948 | 0.165694 | 0.013433 |
| Total.Unigene_50666 | MOC2B | 17.8723 | 0.190071 | 0.015472 |
| Total.Unigene_50667 | MOC2B | 18.871 | 0.177173 | 0.012639 |
| Total.Unigene_50668 | MOC2B | 17.7001 | 0.237997 | 0.022917 |
| Total.Unigene_50669 | MOC2B | 19.1459 | 0.279188 | 0.026292 |
| Total.Unigene_50670 | MOC2B | 20.7424 | 0.263396 | 0.020557 |
| Total.Unigene_50672 | MOC2B | 18.3259 | 0.29276 | 0.031365 |
| Total.Unigene_50674 | MOC2B | 20.358 | 0.353606 | 0.035007 |
| Total.Unigene_50675 | MOC2B | 22.6617 | 0.336331 | 0.02775 |
| Total.Unigene_50775 | LIPR2 | 29.0111 | 0.149927 | 0.004435 |
| Total.Unigene_50776 | LIPR2 | 27.8292 | 0.150552 | 0.004669 |
| Total.Unigene_50777 | LIPR2 | 23.1409 | 0.098035 | 0.003029 |
| Total.Unigene_50778 | LIPR2 | 21.8257 | 0.09848 | 0.003463 |
| Total.Unigene_50825 | PERO | 1127.65 | 4.8655 | 0.007769 |
| Total.Unigene_50945 | CANB2 | 15.3819 | 0.186781 | 0.019519 |
| Total.Unigene_51193 | ALDO2 | 31.8716 | 0.047549 | 0.000654 |
| Total.Unigene_51194 | ALDO2 | 24.2101 | 0.050638 | 0.001434 |
| Total.Unigene_51901 | MOCOS | 36.8064 | 0.27441 | 0.008699 |
| Total.Unigene_52529 | TRET1 | 3.42013 | 0.053895 | 0.040326 |
| Total.Unigene_52530 | TRET1 | 3.42368 | 0.054571 | 0.041105 |
| Total.Unigene_53012 | TRET1 | 14.0559 | 0.047038 | 0.002839 |
| Total.Unigene_53013 | TRET1 | 16.4225 | 0.059957 | 0.002839 |
| Total.Unigene_53014 | TRET1 | 11.7453 | 0.069962 | 0.006304 |
| Total.Unigene_53015 | TRET1 | 11.8789 | 0.009899 | 0.038781 |
| Total.Unigene_53016 | TRET1 | 12.505 | 0.070788 | 0.005766 |
| Total.Unigene_53017 | TRET1 | 12.6788 | 0.085733 | 0.007503 |
| Total.Unigene_53018 | TRET1 | 12.6449 | 0.010017 | 0.037352 |
| Total.Unigene_53019 | TRET1 | 12.8202 | 0.024915 | 0.006724 |
| Total.Unigene_53072 | PGBD4 | 15.579 | 0.318061 | 0.045036 |
| Total.Unigene_7786 | ARSB | 19.3092 | 0.354655 | 0.038543 |
| Total.Unigene_9455 | PP2B3 | 52.4047 | 0.777228 | 0.033094 |
