# Supplementary material for: Silk Gland Gene Expression during Larval-Pupal Transition in the Cotton Leaf Roller Sylepta derogata (Lepidoptera: Pyralidae)
Source: PLoS One. 2015 Sep 9;10(9):e0136868. doi: 10.1371/journal.pone.0136868 (PMC4564283; doi:10.1371/journal.pone.0136868)
Supplement: S2 Table — (DOC) [file pone.0136868.s002.doc]

Supporting Information Table S1. Gene IDs and the partial sequences

| Gene ID | Gene sequence |
| --- | --- |
| 16903 | ATGTTGCGGACGCCGATCGGTGGGACAGACTTCTCGACGCATCCTTACGCATACAACGAA  TTACCTAAAGATGACGCTAACCTTAGCAACTATACACTGTCTACCGAGGATTATCAGTAC  AAGATTCCGATGATCAAAGCGATCTTGGAGACGTCGACCGTCCCTGTTCACATAGTAGCG  ACCTGCTGGTCCCCTCCGCCCTGGATGAAGACCAACGACAATATTACAGGATTCAGCCGA  CTTAAGCCCCAGTACTTCCAGACCTACGCGGATTACCATCTCAAATTCTTGGAAAAATAC  GCAGTGGAAGGCATATCAATTTGGGGCATCACCACTACCAACGAACCAATCAATGGAGTA  TTCAATTTGGCTTCTTTTAACACCCTTGGGTGGACTCCGGGAGAAATGGGAAAGTGGATA  GCAACCAACTTTGGGCCGTTGATTCGTAATTCCACCTTCAAGGACATAAAGATAATGACG  CTTGACGACCAACGGATCGCCATACCATTTTGGTTTAACTTGATGTTGAAGAAGCACCCT  GAAGCTCTGCAGTACATAGATGGAGTGGCGGTGCACTACTATACCGACATGATCATACCA  CCTTCAGTTCTCTCTCTGATCTCGAAGAATTACCCGGATAAGTTCATATTGGCGACTGAG  GCTTGTGAAGGTTCTGTACCATGGGTAACCGAGAAGGTCCTGCTCGGATCATGGCAAAGA  GCTAAGAATTATATCCATGATATTATAGAGGATATGAACCATAATGTGGTTGGCTGGATA  GACTGGAACCTTTGCCTGAAC |
| 25262 | ATGGGTGATATGAATTCATCCGATTGTTCCTGGAAAAAGATTGTGATAGCAAAATCAAAT  AATGAATGGATATCTTCCATCAAACAAGAGCGTTGTGATACTGACGATTCTATGAACATT  ACAAATTTTAGTTTAGACGGTGATCAAGTTGATGAAGACAATGTAAGCGTTAAAACTGAA  AATTACAGCAGCCATCAGCACCAGCATGAGCCAATATCACTTATGCAGGAGGTCGACCCT  CTCATGATCATCTGCGAGCCTCGAAGACGTCGTAATGGGTCAGGACCCAAGATCGAAACC  CCGGAAGAACGCAAGGTTCGGTTGGCGAAAATGTCTGCTTATGCCGCTAAAAGAATAGCC  AACGAGACACCCGAACAACGCGCTGTGAGACTTAAGAGAATGTCCGAATACGCAGCGCGA  AGATTGGCGCAGGAAACCAGTGAACAAAGAGCTGCCCGCTTGGCGAAGATGTCTGCATAT  GCAGCAAAGCGGCTGGCTACGGAGACGCAGGAGCAGAGGCAAGCGCGCCTAGCCCGAATG  TCCGCCTACTCTGCTAAGAGAACAAAGTCTAAGGCCACAGAGAAAAAAATATTCCTCAGT  ACCTTATCAAAACATAAACAT |
| 26685 | ATGGGAAGCGGCAAGTTCGGTTTCAATGCTAAGAAGGACAAAGTGTCAGTGAGTGATACA  AAGACTGAGTTTAGTGTTAAGGAAAAATGTCACAAAATACAGAAAAAACTAAAGAAGACT  GAAAAAAAGAAGACTGATGTTGACAAACAAGAGAAGACTATTTGGTGGAATCAATGCAGC  AGTTTGAGCGAAATAGACCAGTACCTATGGATGGTTGGTGGGTCAGCCGGCGCCCTGGCG  CTGACAGGGGTCGCCGCCGCCTCAGCCTTCTACCTAAGCACAAGGCCAAAACCAGAGAAG  CCACTTGTCACCTTACACGAACAGAGTCTACTCGAAGTGGGTCCTGAGATGGTTCGTGTA  TCAAAATTCTACAAAGATGCGAAAAATGGTCGCTATCTCCGCTACTTACATGAAGATACG  CGTACACTTTATGAAACTTTCCGTAGAGGTGTTAAAGAATCAAATAATGGCAACTGTCTG  GGTTGGCGCGAGGGACCAAACAAACCCTACATTTGGCAAACCTACAATGAAACATTGCTG  CGGGCGAAAAACTTCGGCTCTGGTCTTATATGCCAAGGGCTTGCCCCAGGACAAAATACC  TTCGTTGGTATATACTCACAGAATTGCCCCGAGTGGGTCATGACAGAACAAGCCGCCTAC  TGCTATTCAATGGTCATTGTACCATTGTATGACACGTTAGGGGCTAACGCATGTGCCTTC  ATTGTAAATCAAACCGAAATGACAGTAGTAATTTGTGAAGATGACAAGAAAGCAAATCTC  CTTCTTGACCAATCACCTCGTTGCTTGAGAAAGCTAATCACCATCAAAGAAGTGTCACCC  TCAACGCACCAGAGAGCGAAGAGTCGTGGCGTGGAAATTATGAAATTTAGTGACGTCGAA  ATACAAGGAGCGCAAAAGGATCAACCCTGTATACCACCTAAACCGGAGAGCATATGCACA  ATTTGCTACACGTCAGGCACGACGGGCATGCCGAAAGGCGTGATGCTCACGCACGAGAAC  GTGGTCGCTTCGATGTGCAGCGTGATTATGCAGTTGGGCGAGCATCGGCCGGCTAAGACT  GATGTGATGATTTCCTTCCTGCCACTAGCGCATATGCTCGAGCGGTGTTGTGAGAATGCT  CTCTACACGGTAGGAGGCGCGGTGGGCTTCTACAGCGGCGACATTCGTCGGCTGGCGGAT  GACCTCAGCGCCCTAAAGCCTACCATGATGCCGGCGGTACCTAGGCTACTCAATAGGCTC  TATGATAAGGCGCAATCGGAAATCTCCAATTCGAGAATAAAGAAGCTGCTGTTCAATATG  GCGCTGTCGGCCAAAGAATCCGAATTGAAAAGAGGGATCGTTCGCGGCGACTCGATATGG  GATAAGCTTGTGTTCCGCAAAGTAAGAGAAGGGATGGGTGGCCGTCTCCGCATCATGGTC  GTCGGGTCCGCCCCTCTCGCCGGGAATGTGCTGACTTTCGCTAGATGCGCACTTGGTTGC  CTGATTGTCGAAGGTTATGGCCAGACGGAGTGCACCGCGCCGGTCACTCTGACAGTGCAG  GGCGACCACGTACCTGAACATGTCGGGCCTCCCGTCGCTTGTTGCAAAGTGAAGCTAGTG  GACGTGCCCGAGATGGAGTATTACGCAGCGCAAGGTCAAGGCGAGGTGTGCGTGCAGGGC  GCGAACGTGTTCAAGGGTTACTTCAAGGAGCCCGAGAAGACCCGTGAAGTCATCGACAAC  GATGGATGGCATCATACCGGTGACGTCGGCAAGTGGCTACCGAATGGCACACTTAAAATT  ATAGACAGAAGAAAACACATCTTCAAACTATCGCAAGGCGAGTACATAGTTCCTGAGAAA  ATTGAAAACATATACATAAGAAGTCAATACGTCGAGCAAGTCTTCGTGCACGGAGAATCG  TTAAAGTCGTGCGTAGTAGCAGTAGTGGTTCCTGACGTCGACGTAGTCAAGTGCTGGGCG  CTGGAGAACGGCATCAAAGGCACCTTCTCCAATCTGTGTGAAGACGAGCGAGTGAAGAAG  GTCATCCTAGAAGACATGCTTCAGTGGGGCAGAACTGCTGGCCTGAAAACCTTTGAGCAG  GTGAAAGACATATACCTCCACCCAGACCCATTCTCGGTGCAAAACGGCCTCCTCACACCG  ACTTTGAAGGCGAAACGACCCGAAATCAGAAGCTACTTCAAGCCACAAATAGAGGACATG  TACAAACAACTCGAA |
| 27009 | ATGGTGGACACACAGCACTTCTGCCTGCGCTGGAACAACTACCAGAGCAGCATCACCAGC  GCGTTCGAGAACCTGAGAGACGACGAAGACTTCGTCGATGTGACGCTGGCGTGCGACGGC  AAGAGCCTGAAGGCGCACCGAGTCGTCCTCTCGGCATGCAGCCCATATTTTAGGGAACTA  TTGAAGTCAACACCATGCAAGCACCCAGTCATCGTGCTCCAAGACGTGGCCTTCACCGAC  CTGCACGCGCTCGTCGAGTTCATCTACCACGGCGAGGTGAACGTGCACCAGAGGAGCCTG  TCCTCCTTCCTCAAGACCGCGGAGGTGCTCCGCGTCTCCGGCCTCACGCAGAATGAGGAC  GTGCAGGTGCCACTCCTGCAGACAGTCCGGGCCACCACCACGGCGCCCTCGCCGCACACG  CCGCCCCACCCGGCGCACACGCCACACGCCGCCCACACGCCCAGCTACTCCGAGAAACTC  GAGGAGGCCCTCTCAATCCCTCCCAGTGTACACTCCATGCGCCGTATCCCTCTCCCCCCA  CGCCGCATGAGCCGCTCCGCAGACAACTCCCCTGACGTCATCAAGCGTCCTCGCCACGAC  AACAACAACGAACAGCCTCAAATCCACGACTTCTCAACCAAGAACCACTCGTTGGTGAAC  AACACTCGGGCCCACGAATCGGGGAACAATGGGAATGGAATCTCTAACAGCAGCTCGTCG  CCGTCTCCACGGCTGATGGAGGAAGTGAAGAATGAACCGCTGGACATGATATGCGTGCCG  AATGCTGACATCGACAGGAGCACGGATGACACGCCGCCGCATCATCATAGACCACTTGGA  GGAGGCCCGCCATCTCGCGGCAGCTCGGCCGAAGCGGACGACCGTACGCCGCCGCCCCAA  ATGCCACCATCGCCGTTCGTGCAACCTGCTGACACTAAATTCCCGCCTTACCACTTCAGC  ATGGCGCCACTCTCTGACACGTCTCTTTCTGGGCTGACGAACCCCCTGGTCCCCGACAGC  ATGGCCAGCACATCTCAAGGTGGCGCCCGCACCCCCCAGGAAGATTACCGTTGCGAGCCG  TGTAACAAGAGCTTGTCTTCCCTGACGCGGCTCAAACGGCATATTCAGAACGTTCACATG  AGACCCTCCAGGGAGCCAGTGTGTAACATTTGCCGGCGGGTTTACTCCAGCCTGAACAGC  CTCAGGAACCACAAGTCGATCTACCACCGCAAGCAGCAACAGCCCTCCGCGTCCACCCAG  GGCCCCTTCTACCCCGTCAAC |
| 31375 | ATGCCTCCTAATTGTGATGTGCTAGCAAAAACCGAGTCTGGAATGGTCTGCGGAAAGATT  AGGAAAGCCACAAATGGAGACAAATACGCCAGCTTCCGAGGGATTCCTTATGCGAAGGCG  CCTCTTAAGGAACTAAGGTTTGCGGAACCACAAAAACGAGAACCTTTCAACGGTACCTTC  GATGCATCAGAGTTGGGTCCTATTTGTCCCCAAATTGGTTCAGTCTACGGAGCTCTCCAG  CAGCCGCTCTCGATGGACGAAGACTGCCTGCGCATCAACATCCATGTGCCTTTAGCCTCA  CTACCAGACATTGCCTCTAATAATAGCACGAAGCCGCTGTTACCAGTCTTCGCCTGGATC  CATGGTGGCTCCTTCTCCTACGGTTCTGGTGACACCGACCTTCACGGCCCAGAGTATCTA  CTATCAGGCAAAGGCGTCATCCTTGTAACTTTTAATTACAGGCTCAACGTGTTTGGATTT  CTCACTCTCAATTCTAAAGAGATACCTGGCAACGCTGGTCTCCGTGACGCACTCTTCGCT  TTGAAGTGGATCCAGAAGAATATCAAATTCTTCGGTGGAGACCCGGAAGAAGTCACTTTG  GCCGGTCAGAGTTCTGGTGGGGCGATGGCGCATACGCTGTCTATCTCACCATCTGTGAAA  GAGTCCAGACCGTTTAAGCGACTTATGACGTTCAGTGGAATTGCTACTTCAGGATTCTTC  AGCTCCAGCAAATTTTACTCAGGATTCATTGCAACTGCTTTCCTATTGGCAGCAAAGATA  AACATTCTTCAGAGTCCTCAGGGCATCCATAAGGCACTGATAGAAGCGCCCGTGGAAAGA  CTTCTGCAAGCAAACTCTTTCTTGTTGAATATAACAGGTTTAGTAACCTTTAGTCCTGTG  ATTGAAACCCCACAGCCAGGTTTTACCACAATTCTTCCGAAAGACCCAGAACTACTAGTC  AATGAAAGTTATGGGAAAGAGTATAGAGCCTATCTGAGCTTTACCAATAACGAATGCTAC  ACCTTCAAGAAACGGTTGGAAGAAGTTCGTATAGAAATCCTGTTTAATCTCCTCCCACCT  CTGGTCACACCTTTTAATATTCTTTTCGCGGCGAATATTTTGACAGTGCCGATCTTGGGG  ATAAAGATGAAGAACGAATACTTCAATAACACATTAATTACTTTGGATAAGTTCATGCCT  GCTTGCATAGATGGGTATTACAAGTATCCGGCTATGAGATTAACTCAGAAACGAGCAGCT  GTTAATGCTGAGCCGACGTATCTGGCCCAATTCGGTTATGGCAATCCAAATGATCTGTTC  AAAAGATACCTGATGAGGAACTGGCCCGGCTCTGGACATGCCGAAGACATAGCTTACTTC  TTCAGAGCCAACTCCCAGTTGGGGCCAGATGTGACCAACGAATTGGCCGAAGATAATGAC  TTCTCGAGAATGAAAGCTTGGACGACAGACATCGTTACTCAGTTTATCAAAACCGGGTCT  CCAATACCACAGTACGCAAACCAATCAGCATGGCCTCCAAGTGACCAAAAGTTGTTTTTC  CAGAACTTATTAGTTCCCAACAAGTTAGACTTTCAGCAACCAACGCCATACATGTTGGAT  AAAAAGCAGTTCTTCGATAACTTGTTCAAATTTAGCAGAGGGTTG |
| 33086 | ATGTCTGCATTCAGTGACAAAGAAAATCTTAGTAATGGAATCAACAATGTGCACATCAAG  TCTCCAGTTAAAAACAACGTACTGACTGTAAGAAACGATAACGTAGCTGAAGACAAGATG  GAAGTAGAAAAAGCCAGCAAAGAAGAGGTCGAATTTGACCCGCAGCTTGAGCCGCTGCTG  CGCGAGAACCCACGTCGGTTCGTCATCTTCCCCATCCAGTACCAGGACATCTGGAACATG  TACAAGAAGGCGGAGGCGTCCTTCTGGACCGTGGAGGAGGTGGATCTCTCCAAGGATTTG  TCAGACTGGGACTCATTAAAGGACTGTGAGAGGCACTTTATCAAGCATGTGCTGGCTTTC  TTTGCTGCGTCTGACGGCATTGTCAATGAGAACCTTGTTGAGCGCTTCTCACAAGAAGTA  CAAGTGACCGAAGCAAGATGCTTCTATGGATTCCAGATTGCAATGGAGAATGTGCATTCT  GAAATGTACTCATTACTTATTGACACTTATATTAGAGATCCTAAGGAAAGGGATTTCCTA  TTCAATGCTGTTGAAACATTGCCCTGTGTCAAGAAGAAGGCTGACTGGGCCCTGCAGTGG  ATTGGCAGCAAGACTGCCACCTTCGGCGAGAGAATTGTGGCATTCGCTGCTGTGGAAGGC  ATCTTCTTCTCCGGTAGTTTCGCCTCCATTTTCTGGCTCAAGAAGCGAGGTTTGATGCCA  GGCCTGACTTTCAGTAATGAACTTATATCTAGGGACGAGGGCCTTCACACAGACTTTGCT  TGCCTCATGTTCAAACATCTGGTTCAGAAGCCTAGCAAGGAGCGTGTCCTCAACATTATC  AAGGATGCAGTTGTTATAGAGCAAGAGTTCCTCACAGATGCTCTACCGGTCAGGCTACTT  GGCATGAACTGTGACCTTATGTCTCAATACATTGAGTTTGTAGCCGACAGATTACTTGTT  GACTTGATCGGAGAAAAGCACTACAACACAAAGAATCCATTTGACTTCATGAACCTGATT  TCGCTAGAGGGCAAGACAAACTTCTTCGAAAAGAAAGTTGGTGAATACCAGAAGTGGGGA  GTGATGGCTAACCCAATGGACAATGTGTTCACACTGGACGCTGAATTC |
| 35571 | ATGTTGTGTCCAGAAGTATCTAATTTTCCACCGCCAAAAGTAGTTACTTCTTTCAAAGAA  TATGAAAACTTAGACCCCGTAATCAAAACAATTAAAATGAACAATTTCTACAAATCCCTT  GTTATCACTTGCCCAGACGAGGTGCAAACTCCACAATTAATTCAAGAAAAACTATTAGAA  GACAGTGATTATTATAGAATTTCTAATTGTTCATTGACTGCGTTTTTGGAACCAACATTT  GTGAATAACTTTGTTAAGAAAGGCAACTTATATGGCATATCAGTAGATCGGAATTGTATT  ATACAAAATTGTGCAGCAATTACTCCTGATGGTATTCTGACACTCCATGTACTGGAATAT  GTATTCCAAACTCTTGGTTTAGAAGGAACAAAACGGCCACACAACTTCTGTGAAGTGAGA  ATTGATTTAAAAGACATAAAACATGTTAACAAAATTAGCTGGAGTCTTGGAAAATTAGAA  CCATTTGACTTTTATATATCCTGGGAACCAGATCAAGAAGATGTTTGCCCTTCTTCAATT  GCCAAATACTTTCATGACAGAAATGTTAATGTTTCATTATGTTCACTACAAGTGAGTAAA  GTAAACCCTACAATAAGTGAAATACCATCTTTGGAAGATGTAGATATTGAAGAAATGGTA  GAATGGATTGGAATGTTGTGTCATGATGTGGATGTGTCACCAGTTGAAACTTATATTAGT  ACATACAGCCAGCCAGAAAGTGAAAATGCCTTGAAATCAAATAGAATGTCTGTTCTCATT  GTCAAAGGATTTCTAACTCCCACAATCTTGTCTGATGTTTGTAAGAATGTATCTGAGTAT  ACTTGTTCAAGAGAGTTGCACAATTATTGGACAAGCATAAGCATTCAAAGTGAAGATTGT  TTATGGCAATGGAGTCCAAGCAGCCCAAAAATGTTCCAAGCACATGATTCCTCATGCAAC  ATTTTCTTTACTAAATCAAAGTATAAAGTATATTCAATGGGTCAAATAAAGTATTCA |
| 53478 | ATGTATTACAACCAGCCGATAGGAATAATTGTAGCTGAAAGCCGGCATCTAGCTGATAGA  GCTTCAAAATTGGTTTACGTTACTTATAAAAATGTAACAAAGCCTGTAATTGACATCAAA  GAAGCAAAAAATGATACGAGTAGATTAACTGAGTATTTATCGATAGAAGCTCAAAATAGA  GGAGCTGACGTGGATAAAGTAATAAAAGGCGGGCAAACAATATACTCGCAGTATCATTTT  AATATGGAGACTCTTGTTTGTGTGACACGACCTTCGGAAGATGGCTTGGAAGTGCATAGT  TCTACACAATGGATGGATGGAACACAATTAATGATATCCCGGGCATTGAAACTGGATTCA  AACAGCATAGATGTATACGTTCGTCGCCTAGGGGGCTCATACGGTATCAAAATATCCCGA  GCTTCACAGGTAGCTATTGCATGCAGTCTTGCCTCGTGGAAGTTGAATCGCCCCTGCAGA  TTTATACAATCGCTAACAACTAATATGAGAGCCGTCGGTAAAAGGTTTCCTAGTTGTAAT  GATTTTGAGGTAGCTGTCAACAAAAAAGGTCAAATACAATATGTAGATTATACGTTATAT  GAAGATAATGGCTATAAAATCAATGAAACACTAGCTCAACTCGGTGTTGGGCTTTACTAC  AATATGTATGATAGCTCACGTTGGCGCTTTCGTGGATATAATGTAACAACAGACACTCAT  AAAAACACGTGGTGCAGAGCACCAGGAACATTGGAAGCAGTATCTTTGTGCGAATTTATG  CTGGAACGAATTTCCTACGAACAAGATTTAGATCCTGTAAAAGTACGCTTAGTAAATGTA  GATACAGAGAAACATAATTCCATTATAGAAATGTACAACTTTTTAGAAGCAAAATCAGAT  TACAAGGCAAGAAGAGTTGCTGTTGATAAATTTAACACAGAAAACAGGTGGATAAAGCGA  GGTCTAAGAGTTTCTTTCATGCGCTGGTCACCAGTGGGCGGGCAACGTCTTAATTGCAGC  GTCTCAGTATATCATGGGGATGGGACCGTTGCTATTATTCATTCTGGTATAGAAATGGGT  CAAGGAATAAACACGAAAGCAGTCCAAATTGCTGCATATTTCTTAGGTATATCCGTCGAA  AAAATTATAATTAAGGGGAATAATACTACATGTGGTCCTAATGCATTTATTACTGGTGGC  AGTATTGCCTCACAAAATGTCGGTATAGTAGTGGAAAGAGCTTGTAAAGATTTACAATCA  AATATATCTCCCATTAAAACCCAAATGCCAAATGCCACCTGGGAAGAAATTATTAAAGCT  GCGTACGAAGCAGATATTAGTTTGCAAGGACATGGATTTGTGGGTTTTAATGATGCACAG  GAATATGACATCTATGGTGTTGCTATCGCCGAAGTTGAAGCTGATATATTAACAGGACAG  ACTGAAGTACGTAGAGTAGATTTGATAGAAGATGTTGGACGATCGGTTAATCCTGAGGTA  GATATTGGACAAATTGAAGGTGCTTTTATAATGTCCATGGGGTATTGGACTAGTGAAAAA  ATAGTCCATAATCCGAAAAATGGGGAAATTATCACAGATCGAACATGGAACTACTATGTA  CCACAAGCAAAGGATATTCCTCAGGACTTCAGAGTACATTTGAGGAAAAACTCTTACAGT  ACAGATGCTATTCTTGGGACAAAAGCTGTTGGTGAACCTCCAATGTGTTTAGGAACCGTA  ATACCTTTCGCTATTAGAGAAGCTATTACATCAGCAAGGGTTGAAGCTGGAAAATCTAAA  AAAGAATGGTTTCCAGTAGATGGACCTTATACGGCAGAGGTAATATGCATGAACGCGGCG  ACGAAAACAGAACATTTTAAATTTTATACT |
| 8810 | ATGAGAGTCACAACCTTCGTGATCCTGTGCTGTGCTCTGCAGTATGTTGCTGCAGATGCT  ATTGGGGACAGCATGAAAAACATAGAAAAAAATTACCGCGAAGTAAACGAAACCAAAACA  GACGAATATCAATTGGGCAAAGATGGAGCAATGACGGAAAGATTAACCACCCGAAAGAAG  TTTGAACGCAACGTAGAGCCATCTAAGAACATCTCTGGTGAAGAAAAGATAGTAAGGACC  TTCGTCATCGAGACCGATGCAGATGGTCACGAAACTATCTACGAGGAAGATGTTGTGATC  AAAAAGGTGCCAGGAGCACATGGTGGTAGCTCAGCTAGTTCAGCCGCTGCCGCTAGCTCT  GGTGCTAGCGCTGCTAGCGGTGCTGCTGGAGCCTACGGGGCTGGTGCAGGATCAGCCGCT  AATGCCGCCGCCAATGCTGGACCAGGTGGGCCATATGGACCATACGGACCACGTGGACCA  GGATCAGCT |
| 15676 | ATGCATGTTCGAGTAAGAATATTTGGAAAACCTGACACTATAGTAGTGGTGGAATCTAAA  TTGACGAAGATTGACCAATTCCGAAAGATTATTCGGGATAAGTTTGACGTGGAACCGAAG  TTGCAACGTTTATTTTACGGTGGGAAATTGCTCGAGAATGGCTACACATTTCATGATTAC  AACATCAAGCTCAATGATGTTATCCAACTGATGGTCAAGCTGCAGCCTGAGGAGGAACAT  AAAAAAGAGACTAATAATGTTAAAAAGAGTGAAGACATTGGTAACAGTAAAGAGGAAGAA  AAAAATAAAGTAAACTATGAAGATGCTGAAAGTACTCTGTATGCAATTGGTGATTTGATT  GACATGAGGGATATGGAACAAGGAGCTTGGTTTGAGGGAAAAATTGTCCGAATAGTCTAT  GACAGCAAACACCCTCATAAAAATGAAACAGAAAATAATTCAACTAGTGCTAACCTTGAC  AATGGGGATGCAAAACCTGGCTCAGACAAGGAAAGTGATTTAGAGAATAAACCTCCAGAA  GAATCATTAAGTCCTGAAAGTAAGGCAAAGAAGAAAGGTATAGCAAGATATTTTACTAAG  AGTCCAAAGAGTGTAAAGAAAAAACAAAATGATAAAGAAAGCAGTAATACAGAGAGTACA  CAGAAAACAAATGATGCCAATTTGTTGTACAAAATACAATTGGATTCTGACGAAGATGAC  TCTAATCTTTATTGTAAATTAAAAGATATAAGACCAAGGGCTCGAAATGAAATTGATATA  ACAGACTTGAAAGTTGGACAAAAAGTCATGATTAACCACAACATAGAGGAGATACTGGAG  AAAGGATACTGGTATGACTTTAAAATTGCTGAAATAAAGAAGCTAAGAACAATTCATGAG  CTGGTAGGAACCCTATACTTGGGCCCTGATGCTGTTCCCCAGAACGACACAAGGGCTAGA  GTTCATGATAAAATCTATGCTATTGAGGAAGTAGTTCCCTTGGATCAGAGAACTGAAGAG  TACAGCAAAATGATGGTCACACCACCTCCAAAAAGAGCTTTACCATTGAACTGTTTGACT  TGTCGTGATGATGAGGACGCGCCGTGCAAAGATTGTGGTTGCTACCTATGTTCTGGAAAA  GAATTTCCTGAAAAAATTGTCCTCTGTGATGAGTGCAACAATGGCTACCACATGACTTGC  CTCTCTCCTCCTCTGAAGGAGTTACCTGAAGAAGATTGGTACTGTCCCTCTTGCAAGCGA  GACACCAAGGAGGTGATCGCGCCCGGAGCTGCCAAGCAAGTTAAGAAGTCTAATGCTTCC  AAGAGTAACAGAGATTGGGGTCGAGGCATGGCTTGTGTCGGTAAAACAAAGACATGCGCA  ATGCCAGCTAATCACTTCGGCCCCATTCCTGGTATTGAAGTGGGCATGTGCTGGAGGTTT  AGAATACAGCTATCAGAATCGGGAGTGCATCGTCCGCCAGTGTCGGGCATTCACGGGCGT  GACGTGGAGGGTGCGTACAGCATCGTTTTATCGGGTGGCTACGAAGACGATGTGGACTAC  GGGAACGAGTTCACGTACACGGGCAGCGGCGGCCGCGATCTCTCCGGGAATAAGCGCACG  GCGGAGCAGTCCTGCGATCAGACGCTTACTAGGGAGAACAAGGCGCTAGCACGCAACTGC  GCAGTGAAACAGATCAGTGAGGAGGGTGGGGACGCGGGCGAAGACTGGCGCGGCGGGAAA  CCTGTACGAGTAGTGCGCTCCTACAAGATGCTCAAGCACTTCCCCAAGTTTGCACCAAAA  GAAGGCATACGATATGACGGGATCTACAAAGTAGTGAAATACTAT |
| 19504 | ATGCCGAAAAATAAGAAAAAACAAGAGAGCTCTAGCAGTGATAGTGATGAGGGCCCTGTA  GATAGAAACCCTCCTCCAGAAAAGAAGGCCAAGATGGGATCGAGAACTGATGATAAGGAA  CCTACATGGGTGCTGCAAGGCAAAAAATTGGTCAAAGTTCGTGAATTCAAAGGGAAAGTT  TATGTAGATGTCAGAGAATTCTATGAGAAAAATGGTGAATTGTTACCAGGAAAGAAAGGA  ATCAGTATGACACCAGAACAATGGCGTAAGCTACTGTCTTTAGGTGATGAGATAAATGAA  ACAATAAGTTCTTTATGC |
| 23951 | ATGCTGCCTTTCGTTTTGGTATCGCTCTTTGTGTCGGGCGCGCTCGCCGTGCCAGTAGTT  AACGTCAACCAATACAGCATCAACGAGGTTGCTCCAGTTGGTGACAATGGAAGACTCGTG  TCAAGCTTCTTAACAGACCGTTCATTCGAAGCCGTAGATGGAGGTGACCAAAACATCTAC  ATCCTCACCATCGAACAAATCCTCAATGACCTGGCCAACCAGCCCGACTCTCTCAGCCAA  GCTCTGGCTGTCGGACAAACCATCGCTGTCCTTGGAGAACTCGCCAATGGCGTGCCAGGA  GACTCTTGCGAAGCTGCTGCTCTCGTCAACGCTTACGCTAACGGCGTGAGGTCCGGAAAC  TTCGCCGGAGTCCGTGGAGCTCTCAACAACTTCCTCGGCCGTCTCGCCTCAAACATCGAC  CTCATCGCTCAAGTCGCCGCCAACCCCAACGCTCTGCGTTTCTCAAGCGGACCAAAGGGC  AACTGCGCCGGAGGAGGCAGAACCTACCAATTCGAAGCAGCTTGGGATGCTGTCCTCTCA  AGCGCTAACGCGTACCAAATTGGACTCATCAACGAGGAATACTGCGCTGCCAAGCGCCTC  TACAGCGCCGTGAACATCCGCAGCAACAACGTCGGTGCTGCCGTGTCCGCCGCCGCCGTG  GCTCCAGTCACCCAAGCCGTCCAAGGCGCTCTTGGACCACTCGCCAACTTCCTGAGGGCC  GTTGCCAACGGTGCCAACGCTGCTTCAGTTGCTGGTGCTGCGAAGAGCGCCCTCCTCCAA  GCCGGCGGACGCGTCCAGCTC |
| 25262 | ATGGGTGATATGAATTCATCCGATTGTTCCTGGAAAAAGATTGTGATAGCAAAATCAAAT  AATGAATGGATATCTTCCATCAAACAAGAGCGTTGTGATACTGACGATTCTATGAACATT  ACAAATTTTAGTTTAGACGGTGATCAAGTTGATGAAGACAATGTAAGCGTTAAAACTGAA  AATTACAGCAGCCATCAGCACCAGCATGAGCCAATATCACTTATGCAGGAGGTCGACCCT  CTCATGATCATCTGCGAGCCTCGAAGACGTCGTAATGGGTCAGGACCCAAGATCGAAACC  CCGGAAGAACGCAAGGTTCGGTTGGCGAAAATGTCTGCTTATGCCGCTAAAAGAATAGCC  AACGAGACACCCGAACAACGCGCTGTGAGACTTAAGAGAATGTCCGAATACGCAGCGCGA  AGATTGGCGCAGGAAACCAGTGAACAAAGAGCTGCCCGCTTGGCGAAGATGTCTGCATAT  GCAGCAAAGCGGCTGGCTACGGAGACGCAGGAGCAGAGGCAAGCGCGCCTAGCCCGAATG  TCCGCCTACTCTGCTAAGAGAACAAAGTCTAAGGCCACAGAGAAAAAAATATTCCTCAGT  ACCTTATCAAAACATAAACAT |
| 31375 | AGGAAAGCCACAAATGGAGACAAATACGCCAGCTTCCGAGGGATTCCTTATGCGAAGGCG  CCTCTTAAGGAACTAAGGTTTGCGGAACCACAAAAACGAGAACCTTTCAACGGTACCTTC  GATGCATCAGAGTTGGGTCCTATTTGTCCCCAAATTGGTTCAGTCTACGGAGCTCTCCAG  CAGCCGCTCTCGATGGACGAAGACTGCCTGCGCATCAACATCCATGTGCCTTTAGCCTCA  CTACCAGACATTGCCTCTAATAATAGCACGAAGCCGCTGTTACCAGTCTTCGCCTGGATC  CATGGTGGCTCCTTCTCCTACGGTTCTGGTGACACCGACCTTCACGGCCCAGAGTATCTA  CTATCAGGCAAAGGCGTCATCCTTGTAACTTTTAATTACAGGCTCAACGTGTTTGGATTT  CTCACTCTCAATTCTAAAGAGATACCTGGCAACGCTGGTCTCCGTGACGCACTCTTCGCT  TTGAAGTGGATCCAGAAGAATATCAAATTCTTCGGTGGAGACCCGGAAGAAGTCACTTTG  GCCGGTCAGAGTTCTGGTGGGGCGATGGCGCATACGCTGTCTATCTCACCATCTGTGAAA  GAGTCCAGACCGTTTAAGCGACTTATGACGTTCAGTGGAATTGCTACTTCAGGATTCTTC  AGCTCCAGCAAATTTTACTCAGGATTCATTGCAACTGCTTTCCTATTGGCAGCAAAGATA  AACATTCTTCAGAGTCCTCAGGGCATCCATAAGGCACTGATAGAAGCGCCCGTGGAAAGA  CTTCTGCAAGCAAACTCTTTCTTGTTGAATATAACAGGTTTAGTAACCTTTAGTCCTGTG  ATTGAAACCCCACAGCCAGGTTTTACCACAATTCTTCCGAAAGACCCAGAACTACTAGTC  AATGAAAGTTATGGGAAAGAGTATAGAGCCTATCTGAGCTTTACCAATAACGAATGCTAC  ACCTTCAAGAAACGGTTGGAAGAAGTTCGTATAGAAATCCTGTTTAATCTCCTCCCACCT  CTGGTCACACCTTTTAATATTCTTTTCGCGGCGAATATTTTGACAGTGCCGATCTTGGGG  ATAAAGATGAAGAACGAATACTTCAATAACACATTAATTACTTTGGATAAGTTCATGCCT  GCTTGCATAGATGGGTATTACAAGTATCCGGCTATGAGATTAACTCAGAAACGAGCAGCT  GTTAATGCTGAGCCGACGTATCTGGCCCAATTCGGTTATGGCAATCCAAATGATCTGTTC  AAAAGATACCTGATGAGGAACTGGCCCGGCTCTGGACATGCCGAAGACATAGCTTACTTC  TTCAGAGCCAACTCCCAGTTGGGGCCAGATGTGACCAACGAATTGGCCGAAGATAATGAC  TTCTCGAGAATGAAAGCTTGGACGACAGACATCGTTACTCAGTTTATCAAAACCGGGTCT  CCAATACCACAGTACGCAAACCAATCAGCATGGCCTCCAAGTGACCAAAAGTTGTTTTTC  CAGAACTTATTAGTTCCCAACAAGTTAGACTTTCAGCAACCAACGCCATACATGTTGGAT  AAAAAGCAGTTCTTCGATAACTTGTTCAAATTTAGCAGAGGGTTG |
| 33140 | ATGGGTATTTGCGTGAGCTGCCGGCGCCGCGAGCGGAAGGCACCCGAGAAGCGGTCGGGG  GCAGGGCTGGGCGCGGGCGGGCCCGGTAAGGGCCCAGCGGCGGCTGAACTGCTGCCGCGG  CGCGTCGGACACCTGCCAGCCATGGCCAACTCTATCTCCAACATCAAGATGACCAACCCT  ATCAAGGGAATAGTCAGCAAGCGTCGGAAACGATATATCAAGGACGGTTTCAACTTGGAC  TTAGCCTATATAACGGATAGGCTGATAGCGATGGGCTTCCCGGCGGAGAAGCTGGAGGGC  GTCTACAGGAACCACATAGATGAGGTGTACAGGTTTCTGGAGCAGATGCACAAGGACCAC  TACAAGATCTACAACCTCTGCTCGGAGAGGTCCTACGACTCGAGCAAGTTTCACGAAAGG  GTGGAGAGGTACGCGTTTGAGGACCACACGCCGCCGAAGATGGAGCTGATCCAGCCGTTC  TGCGAGGACGTGCACAAGTGGCTCAGCGAGGACCCAAGGAACGTGGCTGCTGTGCACTGT  AAAGCTGGCAAGGGGAGAACCGGTACAATGGTGTGCTGCTACCTCTTATACAGCGGGCAG  AAGGCAACGGCGGACGAGGCGCTACAGTTTTACGGTACCAAGAGAACGCACGACGAGAAG  GGAGTAACGATACCCTCCCAGAGGCGGTACGTGGAGTACTACGCCGAGCTCGTGCGCTCC  GGGCTGCAGTACACGGCCACGAAGGTGTACATCCGGGAGCTGATCATGTGCCCCCCGCCC  ATGCTGAACGGAGGCCAGTGCACCCTCGAGCTCACCGTCTCACAGGCGCAACCGCCCTTC  AAGGCGCCGCACGGCTGCCACGAGATGCGCAACAACGCGCGCTGCGTGCGCGTGTGCCTG  ACGCACTGCACGCCGCTGCACGGCGACGTGCGCGTGGACGTGTACAACAAGCCCAAGATG  ATGATGCGCAAGGAGAAGCTCTTCCACTTCTGGTTCAACACCTTCTTCCTCGTGGCCGAG  GTGGGCGCGCAGCGGATACCGCCGCCCGCAGACAGTCCGAATCAGGAGACGTACAAGCTA  ACGCTGAATAAGTGGCAGCTGGACGACGCGCACAAGGACAAGCAACACAAGTTATACAGC  CCGGATTTCAAGGTGGAGCTCATAGTACAAAAGCAGCCCGACACGTCCACGTTCAGCGCG  CGCCCGCCGCCGCCCTCGCCGCCCTCCTCCTCCGCCTCCGACGCCTCCGAGCCCGACGCG  CACTGGGACTCCGGGCGCGTGCCGGTTTGCGAGCGCGTGGGCCGCTACCGCCAGCTGTCG  CCCGACGCGCGCACGCACACG |
| 33585 | ATGAAGCCAACATCGAGATGTGCAATCGTACTAATCGTCGCCTGCAGTTATATCCATTTT  GGAGGAGTAGAAGCAGATTATTCAGTCGCAACTGCTTTTGAAGCCCATGAGCTGGTGCCT  GACATCATTCCACAGGCACCGAATGCTTTGGTTACCGTGGAGTACTCAGGTGGACTGTTT  GTGAATTTAGGGAATGAGTTGAAACCTAGCCAGGTTCAAAACGCGCCCTGTGTCTCTTGG  CAGGCGAGTGACAACGAGTATTACTTACTCGCCATGACCGATCCAGATGCGCCGTCCCGC  GCTTCGCCAGATTGGAGGGAAATCAATCACTGGCTGGTAGGAAACATCCCTGGCAACAAC  GTGGCTTCTGGGGAGACCCTCGCGGTCTACATTCCTTCTGCTCCACCAGCAGAT |
| 35672 | ATGAGTTTACATAGTAGGAGAGATATATCGACAAGAATGGCGTTTGTTAGTTTGAGGGTC  AGTCTGAGGTTACTGTGGACGGTGGCTGTGGCGCTGGTGTTCTTCACGAGGAGTAGCGCG  GCCCCGACATTTGGAGGCACCGATAAGGCCATGATGTACCTGGCGCAGTATGGGTATCTG  AGCCCGTCGGTGCGGAACCCTTCCAGTGGTCACATAATGGACGAAAGCTCGTGGAAGCGG  GCAATCGCTGAGTTCCAGAGCTTCGCTGGGTTGAATGCTACAGGTGAACTGGATGAAGAG  ACGACGAAAGTGATGTCGCTGCCGAGGTGCGGCGTGCGAGACAAAGTAGGCTTCGGTGAA  AGTCGTGCCAAAAGATACGCGCTTCAAGGTTCGAGGTGGCGTGTGAAGAATCTGACGTAT  AAAATCTCCAAATATCCCTCAAAACTTAACCGCGCTGAGGTAGACGCAGAGTTGGCGAAA  GCCTTCTCAGTGTGGTCTGACTACACGGATCTCACCTTCACGCAGAAGAGGTCCGGACAG  GTCCACATTGAAATTAGGTTTGAGAAAGGAGAGCACGGTGACGGAGATCCATTCGACGGA  CCCGGAGGCACCCTCGCTCACGCTTACTTCCCTGTGTACGGTGGTGACGCCCACTTCGAT  GATGCCGAGATGTGGACAATCAACTCCAGGAGAGGAACTAATCTCTTCCAGGTGGCTGCG  CACGAGTTCGGCCACTCGCTGGGTCTCTCCCACAGTGACGTGCGCTCTGCGCTCATGGCG  CCCTTCTACAGGGGCTACGACCCTGCCTTCCAGCTCGACCAGGATGACATCCAGGGTATT  CAGGCGCTCTACGGTCACAAGACACAGACCGACATTGGCGGAGGTGTAGCCCCCAGTTTG  CCGACGGGCCCGCGCGTTACCACCGCCCAGCCCTCCGCAGAAGACCCAGCTCTCTGTTCT  GACCCCAAGTTTGACACCATCTTCAACTCTGCTGACGGCGGCACTTTCATCTTCAAAGGC  GAACACTACTGGCGGTTAACGGAAGACGGCGTAGCGGCTGGTTACCCTCGCCTGATCTCT  CGCGCATGGCCTGGTCTCCCCGGCAACATTGACGCCGCTTTCACCTACAAGAATGGCAAG  ACATACTTCTTCAAAGGCTCCAAATACTGGAGGTATAACGGACAGAAGGTCGACGGGGAG  TATCCTAAAGAAATTAGTGAAGGATTCACGGGTATTCCCGACAACATCGACGCTGCGTTA  GTCTGGTCGGGCAATGGCAAGATCTACTTCTACAAAGGTTCCAAATTCTGGAGGTTCGAC  CCGGCCCAGCGCCCGCCCGTCAAGTCGACGTATCCGAAGCCACTGTCCAACTGGGAAGGC  ATCCCCGACGGCATCGACGCCGCATTGCAATATACTAATGGATACACGTACTTCTTCAAG  GGTGGATCGTATTGGCGGTTTAATGACAGGACTTTCAGTGTGGACTCAGATAACCCCGCC  TTCCCCCGGTCCACCGCGTTCTGGTGGCTGGGGTGCAGCAGCGCGCCGCGCGGCACTGTC  GGAGGTGTAAAATCATCCGCTCCCAGATCATTCTTCTGGTTCAGGAAA |
| 35673 | ATGAGTTTACATAGTAGGAGAGATATATCGACAAGAATGGCGTTTGTTAGTTTGAGGGTC  AGTCTGAGGTTACTGTGGACGGTGGCTGTGGCGCTGGTGTTCTTCACGAGGAGTAGCGCG  GCCCCGACATTTGGAGGCACCGATAAGGCCATGATGTACCTGGCGCAGTATGGGTATCTG  AGCCCGTCGGTGCGGAACCCTTCCAGTGGTCACATAATGGACGAAAGCTCGTGGAAGCGG  GCAATCGCTGAGTTCCAGAGCTTCGCTGGGTTGAATGCTACAGGTGAACTGGATGAAGAG  ACGACGAAAGTGATGTCGCTGCCGAGGTGCGGCGTGCGAGACAAAGTAGGCTTCGGTGAA  AGTCGTGCCAAAAGATACGCGCTTCAAGGTTCGAGGTGGCGTGTGAAGAATCTGACGTAT  AAAATCTCCAAATATCCCTCAAAACTTAACCGCGCTGAGGTAGACGCAGAGTTGGCGAAA  GCCTTCTCAGTGTGGTCTGACTACACGGATCTCACCTTCACGCAGAAGAGGTCCGGACAG  GTCCACATTGAAATTAGGTTTGAGAAAGGAGAGCACGGTGACGGAGATCCATTCGACGGA  CCCGGAGGCACCCTCGCTCACGCTTACTTCCCTGTGTACGGTGGTGACGCCCACTTCGAT  GATGCCGAGATGTGGACAATCAACTCCAGGAGAGGAACTAATCTCTTCCAGGTGGCTGCG  CACGAGTTCGGCCACTCGCTGGGTCTCTCCCACAGTGACGTGCGCTCTGCGCTCATGGCG  CCCTTCTACAGGGGCTACGACCCTGCCTTCCAGCTCGACCAGGATGACATCCAGGGTATT  CAGGCGCTCTACGGTCACAAGACACAGACCGACATTGGCGGAGGTGTAGCCCCCAGTTTG  CCGACGGGCCCGCGCGTTACCACCGCCCAGCCCTCCGCAGAAGACCCAGCTCTCTGTTCT  GACCCCAAGTTTGACACCATCTTCAACTCTGCTGACGGCGGCACTTTCATCTTCAAAGGC  GAACACTACTGGCGGTTAACGGAAGACGGCGTAGCGGCTGGTTACCCTCGCCTGATCTCT  CGCGCATGGCCTGGTCTCCCCGGCAACATTGACGCCGCTTTCACCTACAAGAATGGCAAG  ACATACTTCTTCAAAGGCTCCAAATACTGGAGGTATAACGGACAGAAGGTCGACGGGGAG  TATCCTAAAGAAATTAGTGAAGGATTCACGGGTATTCCCGACAACATCGACGCTGCGTTA  GTCTGGTCGGGCAATGGCAAGATCTACTTCTACAAAGGTTCCAAATTCTGGAGGTTCGAC  CCGGCCCAGCGCCCGCCCGTCAAGTCGACGTATCCGAAGCCACTGTCCAACTGGGAAGGC  ATCCCCGACGGCATCGACGCCGCATTGCAATATACTAATGGATACACGTACTTCTTCAAG  GGTGGATCGTATTGGCGGTTTAATGACAGGACTTTCAGTGTGGACTCAGATAACCCCGCC  TTCCCCCGGTCCACCGCGTTCTGGTGGCTGGGGTGCAGCAGCGCGCCGCGCGGCACTGTC  GGAGGTGTAAAATCATCCGCTCCCAGATCATTCTTCTGGTTCAGGAAA |
| 35674 | ATGAGTTTACATAGTAGGAGAGATATATCGACAAGAATGGCGTTTGTTAGTTTGAGGGTC  AGTCTGAGGTTACTGTGGACGGTGGCTGTGGCGCTGGTGTTCTTCACGAGGAGTAGCGCG  GCCCCGACATTTGGAGGCACCGATAAGGCCATGATGTACCTGGCGCAGTATGGGTATCTG  AGCCCGTCGGTGCGGAACCCTTCCAGTGGTCACATAATGGACGAAAGCTCGTGGAAGCGG  GCAATCGCTGAGTTCCAGAGCTTCGCTGGGTTGAATGCTACAGGTGAACTGGATGAAGAG  ACGACGAAAGTGATGTCGCTGCCGAGGTGCGGCGTGCGAGACAAAGTAGGCTTCGGTGAA  AGTCGTGCCAAAAGATACGCGCTTCAAGGTTCGAGGTGGCGTGTGAAGAATCTGACGTAT  AAAATCTCCAAATATCCCTCAAAACTTAACCGCGCTGAGGTAGACGCAGAGTTGGCGAAA  GCCTTCTCAGTGTGGTCTGACTACACGGATCTCACCTTCACGCAGAAGAGGTCCGGACAG  GTCCACATTGAAATTAGGTTTGAGAAAGGAGAGCACGGTGACGGAGATCCATTCGACGGA  CCCGGAGGCACCCTCGCTCACGCTTACTTCCCTGTGTACGGTGGTGACGCCCACTTCGAT  GATGCCGAGATGTGGACAATCAACTCCAGGAGAGGAACTAATCTCTTCCAGGTGGCTGCG  CACGAGTTCGGCCACTCGCTGGGTCTCTCCCACAGTGACGTGCGCTCTGCGCTCATGGCG  CCCTTCTACAGGGGCTACGACCCTGCCTTCCAGCTCGACCAGGATGACATCCAGGGTATT  CAGGCGCTCTACGGTCACAAGACACAGACCGACATTGGCGGAGGTGTAGCCCCCAGTTTG  CCGACGGGCCCGCGCGTTACCACCGCCCAGCCCTCCGCAGAAGACCCAGCTCTCTGTTCT  GACCCCAAGTTTGACACCATCTTCAACTCTGCTGACGGCGGCACTTTCATCTTCAAAGGC  GAACACTACTGGCGGTTAACGGAAGACGGCGTAGCGGCTGGTTACCCTCGCCTGATCTCT  CGCGCATGGCCTGGTCTCCCCGGCAACATTGACGCCGCTTTCACCTACAAGAATGGCAAG  ACATACTTCTTCAAAGGCTCCAAATACTGGAGGTATAACGGACAGAAGGTCGACGGGGAG  TATCCTAAAGAAATTAGTGAAGGATTCACGGGTATTCCCGACAACATCGACGCTGCGTTA  GTCTGGTCGGGCAATGGCAAGATCTACTTCTACAAAGGTTCCAAATTCTGGAGGTTCGAC  CCGGCCCAGCGCCCGCCCGTCAAGTCGACGTATCCGAAGCCACTGTCCAACTGGGAAGGC  ATCCCCGACGGCATCGACGCCGCATTGCAATATACTAATGGATACACGTACTTCTTCAAG  GGTGGATCGTATTGGCGGTTTAATGACAGGACTTTCAGTGTGGACTCAGATAACCCCGCC  TTCCCCCGGTCCACCGCGTTCTGGTGGCTGGGGTGCAGCAGCGCGCCGCGCGGCACTGTC  GGAGGTGTAAAATCATCCGCTCCCAGATCATTCTTCTGGTTCAGGAAA |
| 35675 | ATGAGTTTACATAGTAGGAGAGATATATCGACAAGAATGGCGTTTGTTAGTTTGAGGGTC  AGTCTGAGGTTACTGTGGACGGTGGCTGTGGCGCTGGTGTTCTTCACGAGGAGTAGCGCG  GCCCCGACATTTGGAGGCACCGATAAGGCCATGATGTACCTGGCGCAGTATGGGTATCTG  AGCCCGTCGGTGCGGAACCCTTCCAGTGGTCACATAATGGACGAAAGCTCGTGGAAGCGG  GCAATCGCTGAGTTCCAGAGCTTCGCTGGGTTGAATGCTACAGGTGAACTGGATGAAGAG  ACGACGAAAGTGATGTCGCTGCCGAGGTGCGGCGTGCGAGACAAAGTAGGCTTCGGTGAA  AGTCGTGCCAAAAGATACGCGCTTCAAGGTTCGAGGTGGCGTGTGAAGAATCTGACGTAT  AAAATCTCCAAATATCCCTCAAAACTTAACCGCGCTGAGGTAGACGCAGAGTTGGCGAAA  GCCTTCTCAGTGTGGTCTGACTACACGGATCTCACCTTCACGCAGAAGAGGTCCGGACAG  GTCCACATTGAAATTAGGTTTGAGAAAGGAGAGCACGGTGACGGAGATCCATTCGACGGA  CCCGGAGGCACCCTCGCTCACGCTTACTTCCCTGTGTACGGTGGTGACGCCCACTTCGAT  GATGCCGAGATGTGGACAATCAACTCCAGGAGAGGAACTAATCTCTTCCAGGTGGCTGCG  CACGAGTTCGGCCACTCGCTGGGTCTCTCCCACAGTGACGTGCGCTCTGCGCTCATGGCG  CCCTTCTACAGGGGCTACGACCCTGCCTTCCAGCTCGACCAGGATGACATCCAGGGTATT  CAGGCGCTCTACGGTCACAAGACACAGACCGACATTGGCGGAGGTGTAGCCCCCAGTTTG  CCGACGGGCCCGCGCGTTACCACCGCCCAGCCCTCCGCAGAAGACCCAGCTCTCTGTTCT  GACCCCAAGTTTGACACCATCTTCAACTCTGCTGACGGCGGCACTTTCATCTTCAAAGGC  GAACACTACTGGCGGTTAACGGAAGACGGCGTAGCGGCTGGTTACCCTCGCCTGATCTCT  CGCGCATGGCCTGGTCTCCCCGGCAACATTGACGCCGCTTTCACCTACAAGAATGGCAAG  ACATACTTCTTCAAAGGCTCCAAATACTGGAGGTATAACGGACAGAAGGTCGACGGGGAG  TATCCTAAAGAAATTAGTGAAGGATTCACGGGTATTCCCGACAACATCGACGCTGCGTTA  GTCTGGTCGGGCAATGGCAAGATCTACTTCTACAAAGGTTCCAAATTCTGGAGGTTCGAC  CCGGCCCAGCGCCCGCCCGTCAAGTCGACGTATCCGAAGCCACTGTCCAACTGGGAAGGC  ATCCCCGACGGCATCGACGCCGCATTGCAATATACTAATGGATACACGTACTTCTTCAAG  GGTGGATCGTATTGGCGGTTTAATGACAGGACTTTCAGTGTGGACTCAGATAACCCCGCC  TTCCCCCGGTCCACCGCGTTCTGGTGGCTGGGGTGCAGCAGCGCGCCGCGCGGCACTGTC  GGAGGTAACGCGCGCATCACGCACCACTCGGACTCCACCTCCGAGGACGATGTCGGAGAC  ATACTCTTCGACGCAGTGGTCAACGTTCAGTCAGGAGGGGCTAGACTG |
| 35676 | ATGAGTTTACATAGTAGGAGAGATATATCGACAAGAATGGCGTTTGTTAGTTTGAGGGTC  AGTCTGAGGTTACTGTGGACGGTGGCTGTGGCGCTGGTGTTCTTCACGAGGAGTAGCGCG  GCCCCGACATTTGGAGGCACCGATAAGGCCATGATGTACCTGGCGCAGTATGGGTATCTG  AGCCCGTCGGTGCGGAACCCTTCCAGTGGTCACATAATGGACGAAAGCTCGTGGAAGCGG  GCAATCGCTGAGTTCCAGAGCTTCGCTGGGTTGAATGCTACAGGTGAACTGGATGAAGAG  ACGACGAAAGTGATGTCGCTGCCGAGGTGCGGCGTGCGAGACAAAGTAGGCTTCGGTGAA  AGTCGTGCCAAAAGATACGCGCTTCAAGGTTCGAGGTGGCGTGTGAAGAATCTGACGTAT  AAAATCTCCAAATATCCCTCAAAACTTAACCGCGCTGAGGTAGACGCAGAGTTGGCGAAA  GCCTTCTCAGTGTGGTCTGACTACACGGATCTCACCTTCACGCAGAAGAGGTCCGGACAG  GTCCACATTGAAATTAGGTTTGAGAAAGGAGAGCACGGTGACGGAGATCCATTCGACGGA  CCCGGAGGCACCCTCGCTCACGCTTACTTCCCTGTGTACGGTGGTGACGCCCACTTCGAT  GATGCCGAGATGTGGACAATCAACTCCAGGAGAGGAACTAATCTCTTCCAGGTGGCTGCG  CACGAGTTCGGCCACTCGCTGGGTCTCTCCCACAGTGACGTGCGCTCTGCGCTCATGGCG  CCCTTCTACAGGGGCTACGACCCTGCCTTCCAGCTCGACCAGGATGACATCCAGGGTATT  CAGGCGCTCTACGGTCACAAGACACAGACCGACATTGGCGGAGGTGTAGCCCCCAGTTTG  CCGACGGGCCCGCGCGTTACCACCGCCCAGCCCTCCGCAGAAGACCCAGCTCTCTGTTCT  GACCCCAAGTTTGACACCATCTTCAACTCTGCTGACGGCGGCACTTTCATCTTCAAAGGC  GAACACTACTGGCGGTTAACGGAAGACGGCGTAGCGGCTGGTTACCCTCGCCTGATCTCT  CGCGCATGGCCTGGTCTCCCCGGCAACATTGACGCCGCTTTCACCTACAAGAATGGCAAG  ACATACTTCTTCAAAGGCTCCAAATACTGGAGGTATAACGGACAGAAGGTCGACGGGGAG  TATCCTAAAGAAATTAGTGAAGGATTCACGGGTATTCCCGACAACATCGACGCTGCGTTA  GTCTGGTCGGGCAATGGCAAGATCTACTTCTACAAAGGTTCCAAATTCTGGAGGTTCGAC  CCGGCCCAGCGCCCGCCCGTCAAGTCGACGTATCCGAAGCCACTGTCCAACTGGGAAGGC  ATCCCCGACGGCATCGACGCCGCATTGCAATATACTAATGGATACACGTACTTCTTCAAG  GGTGGATCGTATTGGCGGTTTAATGACAGGACTTTCAGTGTGGACTCAGATAACCCCGCC  TTCCCCCGGTCCACCGCGTTCTGGTGGCTGGGGTGCAGCAGCGCGCCGCGCGGCACTGTC  GGAGGTAACGCGCGCATCACGCACCACTCGGACTCCACCTCCGAGGACGATGTCGGAGAC  ATACTCTTCGACGCAGATGCGGGAGAAAGTTCCGGTAGAAGTGGTAATAGCAGTACCAAG  TGTGGCGAGATGTCGTTCGTGTTCGCGCTGGTCATGGCTGTAATAGCGCGGGTATTCGCG  CGAGCC |
| 37748 | TGAATAACAATCAATTCCAAGAGTTGTTCGGGACGCAGTGGCCGCCCGACCAGCATGGA  GGTCACTCTTCGGCCTCCACTATGCTGCATCAGGGTTTGACCCAGGGAATGTTGAAGAGG  GAACCGCATACTGACGTCCAAGGGTCGAGTATGATGCAGCATCAGATGGGAATGGACATC  ACCTCAGGGTCCGTCGCTGATAGCACATCACCGCCTCCAGGCAGCAGTGAGGGAATGTTC  GGGTCCTCAATATCCGGAATGTTTATGGATAAGAAGGCTGCCAATTCTATACGAGCTCAA  ATCGAGATCATACCATGCAAAGTATGCGGAGACAAGTCTTCGGGGGTGCATTATGGCGTC  ATCACCTGTGAGGGATGCAAAGGGTTCTTCAGGCGGTCACAGAGTACAGTGGTGAACTAC  CAGTGCCCGCGCAACAAGGCATGCGTGGTGGACCGCGTCAATCGCAACCGGTGCCAGTAC  TGCCGACTCCAGAAGTGCCTGAAGCTGGGCATGAGCCGTGATGCGGTGAAATTCGGCCGT  ATGTCAAAGAAACAGCGTGAAAAGGTGGAAGATGAGGTCCGAGAGTTACTACTACTCCAA  GGTGGGATGGAGGACCCAAAAAATGTGAAGTTCTACCGCGCACAGTCGCGTGCGCAGACG  GACGCCGCGCCCGACTCAGTGTACGACGCGCAGCAACAGACGCCAAGCTCCAGCGACCAG  TTCCACGGACACTATAACGGGTATCCCGGGTATGGTTCGCCCCTGTCCCCGTATGGGTAC  AACAACGCCGGGCCAGCGCTCACCTCCAACATGGGTTCGTTGGCGCAGGCGCCCCCCCAG  CAGCAGTCATACGACGTTTCTGCAGACTACGCTGTAGATTCGACCACATATGAGCCTAAG  CAGACAGGATTCCTCGATACAGACTTTATAGGAAACGCTGAAGGCGACATCAGCAAAGTC  CTCGTGAAGAGCCTGGCCGAGGCTCACGCCAACACCAACCCCAAGCTGGAGTACATACAT  GAGATGTTCCGGAAACCACCAGATGTCTCTAAGCTCCTATTCTACAACTCGATGACTTAC  GAGGAGATGTGGCTGGACTGTGCGGACAAGCTCACCGGGATGATCCAGAACATCATCGAG  TTCGCAAAACTGATACCAGGGTTTATGAAGCTCACGCAAGATGATCAGATTCTGCTGCTT  AAGTCAGGTTCCTTCGAGCTGGCTATCGTGCGTCTGTCGAGACTGATCGACGTGAACAGA  GACCAAGTGCTGTATGGAGACGTTGTTCTGCCTATCAGAGAATGTGTGCATGCGCGTGAT  CCACGTGATATGGCGCTAGTGGTGGGTATCTTCGACGCCGCCAAGACAATAGCGCGACTC  AAGCTCACGGAAACAGAGCTGGCGCTCTACCAGAGCCTCGTTTTATTGTGGCCAGAACGA  CATGGCGTCCGAGGCAATCCAGAGATCCAGTGTCTGTTTAATATGTCGATGGCGGCAATG  AGACACGAAATCGAGACCAATCACGCGCCGCTGAAGGGTGACGTCACCGTGCTGGACACG  CTTATCACCAAGATACCCACCTTCAGAGAACTGTCGCTGATGCATTTGGAAGCGCTCTGC  CGCTTCAAGACGGCCCATCCACATCATGTGTTCCCGGCGCTCTACAAAGAGTTATTTTCT  TTAGACAGCGTGTTAGACTACACACACGGC |
| 37749 | ATGAATAACAATCAATTCCAAGAGTTGTTCGGGACGCAGTGGCCGCCCGACCAGCATGGA  GGTCACTCTTCGGCCTCCACTATGCTGCATCAGGGTTTGACCCAGGGAATGTTGAAGAGG  GAACCGCATACTGACGTCCAAGGGTCGAGTATGATGCAGCATCAGATGGGAATGGACATC  ACCTCAGGGTCCGTCGCTGATAGCACATCACCGCCTCCAGGCAGCAGTGAGGGAATGTTC  GGGTCCTCAATATCCGGAATGTTTATGGATAAGAAGGCTGCCAATTCTATACGAGCTCAA  ATCGAGATCATACCATGCAAAGTATGCGGAGACAAGTCTTCGGGGGTGCATTATGGCGTC  ATCACCTGTGAGGGATGCAAAGGGTTCTTCAGGCGGTCACAGAGTACAGTGGTGAACTAC  CAGTGCCCGCGCAACAAGGCATGCGTGGTGGACCGCGTCAATCGCAACCGGTGCCAGTAC  TGCCGACTCCAGAAGTGCCTGAAGCTGGGCATGAGCCGTGATGCGGTGAAATTCGGCCGT  ATGTCAAAGAAACAGCGTGAAAAGGTGGAAGATGAGGTGAAGTTCTACCGCGCACAGTCG  CGTGCGCAGACGGACGCCGCGCCCGACTCAGTGTACGACGCGCAGCAACAGACGCCAAGC  TCCAGCGACCAGTTCCACGGACACTATAACGGGTATCCCGGGTATGGTTCGCCCCTGTCC  CCGTATGGGTACAACAACGCCGGGCCAGCGCTCACCTCCAACATGGGTTCGTTGGCGCAG  GCGCCCCCCCAGCAGCAGTCATACGACGTTTCTGCAGACTACGCTGTAGATTCGACCACA  TATGAGCCTAAGCAGACAGGATTCCTCGATACAGACTTTATAGGAAACGCTGAAGGCGAC  ATCAGCAAAGTCCTCGTGAAGAGCCTGGCCGAGGCTCACGCCAACACCAACCCCAAGCTG  GAGTACATACATGAGATGTTCCGGAAACCACCAGATGTCTCTAAGCTCCTATTCTACAAC  TCGATGACTTACGAGGAGATGTGGCTGGACTGTGCGGACAAGCTCACCGGGATGATCCAG  AACATCATCGAGTTCGCAAAACTGATACCAGGGTTTATGAAGCTCACGCAAGATGATCAG  ATTCTGCTGCTTAAGTCAGGTTCCTTCGAGCTGGCTATCGTGCGTCTGTCGAGACTGATC  GACGTGAACAGAGACCAAGTGCTGTATGGAGACGTTGTTCTGCCTATCAGAGAATGTGTG  CATGCGCGTGATCCACGTGATATGGCGCTAGTGGTGGGTATCTTCGACGCCGCCAAGACA  ATAGCGCGACTCAAGCTCACGGAAACAGAGCTGGCGCTCTACCAGAGCCTCGTTTTATTG  TGGCCAGAACGACATGGCGTCCGAGGCAATCCAGAGATCCAGTGTCTGTTTAATATGTCG  ATGGCGGCAATGAGACACGAAATCGAGACCAATCACGCGCCGCTGAAGGGTGACGTCACC  GTGCTGGACACGCTTATCACCAAGATACCCACCTTCAGAGAACTGTCGCTGATGCATTTG  GAAGCGCTCTGCCGCTTCAAGACGGCCCATCCACATCATGTGTTCCCGGCGCTCTACAAA  GAGTTATTTTCTTTAGACAGCGTGTTAGACTACACACACGGC |
| 37750 | ATGTCAAAGAAACAGCGTGAAAAGGTGGAAGATGAGGTCCGAGAGTTACTACTACTCCAA  GGTGGGATGGAGGACCCAAAAAATGTGAAGTTCTACCGCGCACAGTCGCGTGCGCAGACG  GACGCCGCGCCCGACTCAGTGTACGACGCGCAGCAACAGACGCCAAGCTCCAGCGACCAG  TTCCACGGACACTATAACGGGTATCCCGGGTATGGTTCGCCCCTGTCCCCGTATGGGTAC  AACAACGCCGGGCCAGCGCTCACCTCCAACATGGGTTCGTTGGCGCAGGCGCCCCCCCAG  CAGCAGTCATACGACGTTTCTGCAGACTACGCTGTAGATTCGACCACATATGAGCCTAAG  CAGACAGGATTCCTCGATACAGACTTTATAGGAAACGCTGAAGGCGACATCAGCAAAGTC  CTCGTGAAGAGCCTGGCCGAGGCTCACGCCAACACCAACCCCAAGCTGGAGTACATACAT  GAGATGTTCCGGAAACCACCAGATGTCTCTAAGCTCCTATTCTACAACTCGATGACTTAC  GAGGAGATGTGGCTGGACTGTGCGGACAAGCTCACCGGGATGATCCAGAACATCATCGAG  TTCGCAAAACTGATACCAGGGTTTATGAAGCTCACGCAAGATGATCAGATTCTGCTGCTT  AAGTCAGGTTCCTTCGAGCTGGCTATCGTGCGTCTGTCGAGACTGATCGACGTGAACAGA  GACCAAGTGCTGTATGGAGACGTTGTTCTGCCTATCAGAGAATGTGTGCATGCGCGTGAT  CCACGTGATATGGCGCTAGTGGTGGGTATCTTCGACGCCGCCAAGACAATAGCGCGACTC  AAGCTCACGGAAACAGAGCTGGCGCTCTACCAGAGCCTCGTTTTATTGTGGCCAGAACGA  CATGGCGTCCGAGGCAATCCAGAGATCCAGTGTCTGTTTAATATGTCGATGGCGGCAATG  AGACACGAAATCGAGACCAATCACGCGCCGCTGAAGGGTGACGTCACCGTGCTGGACACG  CTTATCACCAAGATACCCACCTTCAGAGAACTGTCGCTGATGCATTTGGAAGCGCTCTGC  CGCTTCAAGACGGCCCATCCACATCATGTGTTCCCGGCGCTCTACAAAGAGTTATTTTCT  TTAGACAGCGTGTTAGACTACACACACGGC |
| 37751 | ATGTCAAAGAAACAGCGTGAAAAGGTGGAAGATGAGGTGAAGTTCTACCGCGCACAGTCG  CGTGCGCAGACGGACGCCGCGCCCGACTCAGTGTACGACGCGCAGCAACAGACGCCAAGC  TCCAGCGACCAGTTCCACGGACACTATAACGGGTATCCCGGGTATGGTTCGCCCCTGTCC  CCGTATGGGTACAACAACGCCGGGCCAGCGCTCACCTCCAACATGGGTTCGTTGGCGCAG  GCGCCCCCCCAGCAGCAGTCATACGACGTTTCTGCAGACTACGCTGTAGATTCGACCACA  TATGAGCCTAAGCAGACAGGATTCCTCGATACAGACTTTATAGGAAACGCTGAAGGCGAC  ATCAGCAAAGTCCTCGTGAAGAGCCTGGCCGAGGCTCACGCCAACACCAACCCCAAGCTG  GAGTACATACATGAGATGTTCCGGAAACCACCAGATGTCTCTAAGCTCCTATTCTACAAC  TCGATGACTTACGAGGAGATGTGGCTGGACTGTGCGGACAAGCTCACCGGGATGATCCAG  AACATCATCGAGTTCGCAAAACTGATACCAGGGTTTATGAAGCTCACGCAAGATGATCAG  ATTCTGCTGCTTAAGTCAGGTTCCTTCGAGCTGGCTATCGTGCGTCTGTCGAGACTGATC  GACGTGAACAGAGACCAAGTGCTGTATGGAGACGTTGTTCTGCCTATCAGAGAATGTGTG  CATGCGCGTGATCCACGTGATATGGCGCTAGTGGTGGGTATCTTCGACGCCGCCAAGACA  ATAGCGCGACTCAAGCTCACGGAAACAGAGCTGGCGCTCTACCAGAGCCTCGTTTTATTG  TGGCCAGAACGACATGGCGTCCGAGGCAATCCAGAGATCCAGTGTCTGTTTAATATGTCG  ATGGCGGCAATGAGACACGAAATCGAGACCAATCACGCGCCGCTGAAGGGTGACGTCACC  GTGCTGGACACGCTTATCACCAAGATACCCACCTTCAGAGAACTGTCGCTGATGCATTTG  GAAGCGCTCTGCCGCTTCAAGACGGCCCATCCACATCATGTGTTCCCGGCGCTCTACAAA  GAGTTATTTTCTTTAGACAGCGTGTTAGACTACACACACGGC |
| 37831 | ATGTCCCGGCACATGCTTTTGTCGCGGCTGGCGGAGCAGTTCAAGCCTTCGTCGCCGCCG  CCAGCGCACCGCTTCCCCTCGCCCGAGCGCAGCGTCACTCCTCACTCGCCCACCTCGCCG  CACTCGCCGCCGCACTCGCCCGACTCGCTCGCACACCACCGCCTCGACCTGCTGCAGCTG  GGCGACATCGAGATTGCAGACCGAGCGCTGAAGCAATACGCCCGAGCTACAGAGCTTGGT  GCGCTGATGTACTGCAGCGGTTTGGGCGCCCTCTACGGTGGCGCAGGAGTGTGGCCGCTG  CTGCTGCCGCGCGCTTCGCCACAGTTCGCCCATGCGCTCGACCCCGCGCGTCACACGCCG  CCCAGAGATGACGACGAGGAGGAGCTGCCATTGAATTTGTCAACGAAAAACCGCCAGATA  TGGTCCCCTGGTAGTGCATGCGAGCGCGAACAAGAAGCGGACTCGCCTGCCTCTCGGTGG  GACCCGGACGAGTGCGAAGCTCCCCTCGAGCTGGTGAAGCGGTGCCGCAGCAGCCCCGAC  GCCGACGAGCACGAGTCCAGCACGGAGTCCCGCCACTGCCCCTCCGCCCCCGCACACCAA  AACTATTCTCTACCAGCCCCTCAGCAAGACCTCAACTTCTCACTACTCATCAAAAATGAA  AACAAAAACGAAAAGTCATTCCAGTGTAAGCAATGTGGGAAGTGCTTCAAGCGGTCGAGC  ACGCTGTCCACGCACCTCTTGATCCACTCGGACACGCGACCTTACCCGTGCCAGTACTGC  GGGAAGCGCTTCCACCAGAAATCTGACATGAAGAAACACACCTACATACACACAGGAGAG  AAGCCGCACAAGTGCGTGGTTTGTTCAAAGGCGTTTAGTCAGAGCTCAAATCTCATCACG  CATATGCGCAAGCACACCGGGTACAAGCCCTTCTCGTGCGGGCTGTGCGACAAGGCGTTC  CAGCGCAAAGTCGACCTGCGCCGGCACCGGGACTCGCAGCACTCAGACGCAGCCGACGCA  CCCTTGACCGGCATCCCTCCGCCACACCGCTACAGGTTCTATGGAGACGAAAGCACACCC  CTCACACAGCTCATCACGAAT |
| 41695 | ATGCATAGGACGCGGCAGCGGTGGAAGAATTTGAAGTGTGAAAAAAGCGGGTTCATCACG  CCACCCCAACAGAATGGCGGGGAGGAGTCACCGCCCCCAGAGAAGGAGAGGGCGGACCAA  GAGGACACAGAGAAGAGCCACGCGGGGCCCGTGCCGCACTGCGCGGAGGGGCCCAACGAT  GACCTCTACGCCATCCCGGTCAAGCTGAGGCCGAAGAAGGAGCCGCAGTTGCCGCCGGGA  TGGGAGAAGCATGAGGACAACGACGGCCCCTACTACTGGCACATAAAAAGCGGCACAATC  CAACGCGAGATTCCCATGATGCCGCCAGTGGAAGCCAAGGAGTCTCGCATCTCCATGGTT  CGGGACTGCTCAGCATTGTCCGAGGCTGGGAAGTACGATGGCCCCATGACCACGTCTGTT  ACTCGGAGCACCACGAGCGGAGCGTTGGACCATGTCGACCAGGATCACGAGAGGAAGAGG  CGGGAAGAGATGTCTTACAAGCGCCGCAGCTTCCCCGCCCGCCCAGAGCCGGACAACGGG  CGCGCCGTCCGCTTCTTCGTCCGCTCCCTGGGGTGGGTGGAGATCTCGGAGGCCGACCTC  ACGCCGGAGCGCTCCAGCCGCGCCGTCAACAAGTGCATCGTGGATCTCAGCCTGGGACGC  AATGATTTGCTGGACCAAGTCGGTCGTTGGGGTGACGGTAAGGATCTCTTCATGGACCTG  GATGATGGGGCTTTGAAGCTGATCGACCCAGAGAGCCTGACTACACTTCACACACAGCCC  ATCCATACTATTCGCGTATGGGGTGTCGGCAGAGACAATGGACGGGATTTCGCGTACGTG  GCCCGCGACCGCGCGACGCGCAAGCACATGTGCCACGTGTTCCGCTGCGAGGCGCCGGCG  CGCAGCATCGCCAACGCGCTGCGCGACATCTGCAAGCGCATCATGATCGAGCGGTCGCTG  CAGCCGCCACCGCGGCCGACTGATTTGCCGGCTGCGCGCCGTCCGCGACCGCTAGCAGGC  GCGTCGTTCCCCACGCCGATGGAGGAGCCGCGCAAGACGGTGCGCGCGCGCTACCTGGGC  AGCGCGGAGGTGCCGCGCGCGACCGGCATGGACGTGCTCAACGACGCGCTCGACCGCCTG  GCCGCGGCGCGCGCGCCCTCCGCCTGGCGGCCCGTCGCCGTGGCCGTCGCTCCGTCCATG  ATCACCATTACTGAGGAAGGGGAGACGACTCCGATAGTGGAGTGTCGCGTGCGGTACCTT  TCGTTCCTGGGCATCGGGCGCGACGTGCGGCGCTGCGCGTTCATCGTCCACACGGCGCAG  GGGCTCTTCGTTGCGCACGCGTTCCACGCCGAGCCTTCTTCTGGCGCGCTTTGCAAGACC  ATTGAGGCCGCTTGCAAGCTCCGCTACCAGAAGTGTTTGGACGCTCACGGCGGCGCGGCG  TCGCTGGCCAGCGGCGGCAGCGGCGGCGGCAGCAGCGGCGGCAGCGACGCCAGCCGCGCG  TCGCTCGGGCAAGCGCTCAAGTCGCTCGTGGGCTCGCTCACCGGCCGCAGGGGCTCG |
| 41696 | ATGCATAGGACGCGGCAGCGGTGGAAGAATTTGAAGTGTGAAAAAAGCGGGTTCATCACG  CCACCCCAACAGAATGGCGGGGAGGAGTCACCGCCCCCAGAGAAGGAGAGGGCGGACCAA  GAGGACACAGAGAAGAGCCACGCGGGGCCCGTGCCGCACTGCGCGGAGGGGCCCAACGAT  GACCTCTACGCCATCCCGGTCAAGCTGAGGCCGAAGAAGGAGCCGCAGTTGCCGCCGGGA  TGGGAGAAGCATGAGGACAACGACGGCCCCTACTACTGGCACATAAAAAGCGGCACAATC  CAACGCGAGATTCCCATGATGCCGCCAGTGGAAGCCAAGGAGTCTCGCATCTCCATGGTT  CGGGACTGCTCAGCATTGTCCGAGGCTGGGAAGTACGATGGCCCCATGACCACGTCTGTT  ACTCGGAGCACCACGAGCGGAGCGTTGGACCATGTCGACCAGGATCACGAGAGGAAGAGG  CGGGAAGAGATGTCTTACAAGCGCCGCAGCTTCCCCGCCCGCCCAGAGCCGGACAACGGG  CGCGCCGTCCGCTTCTTCGTCCGCTCCCTGGGGTGGGTGGAGATCTCGGAGGCCGACCTC  ACGCCGGAGCGCTCCAGCCGCGCCGTCAACAAGTGCATCGTGGATCTCAGCCTGGGACGC  AATGATTTGCTGGACCAAGTCGGTCGTTGGGGTGACGGTAAGGATCTCTTCATGGACCTG  GATGATGGGGCTTTGAAGCTGATCGACCCAGAGAGCCTGACTACACTTCACACACAGCCC  ATCCATACTATTCGCGTATGGGGTGTCGGCAGAGACAATGGACGGGATTTCGCGTACGTG  GCCCGCGACCGCGCGACGCGCAAGCACATGTGCCACGTGTTCCGCTGCGAGGCGCCGGCG  CGCAGCATCGCCAACGCGCTGCGCGACATCTGCAAGCGCATCATGATCGAGCGGTCGCTG  CAGCCGCCACCGCGGCCGACTGATTTGCCGGCTGCGCGCCGTCCGCGACCGCTAGCAGGC  GCGTCGTTCCCCACGCCGATGGAGGAGCCGCGCAAGACGGTGCGCGCGCGCTACCTGGGC  AGCGCGGAGGTGCCGCGCGCGACCGGCATGGACGTGCTCAACGACGCGCTCGACCGCCTG  GCCGCGGCGCGCGCGCCCTCCGCCTGGCGGCCCGTCGCCGTGGCCGTCGCTCCGTCCATG  ATCACCATTACTGAGGAAGGGGAGACGACTCCGATAGTGGAGTGTCGCGTGCGGTACCTT  TCGTTCCTGGGCATCGGGCGCGACGTGCGGCGCTGCGCGTTCATCGTCCACACGGCGCAG  GGGCTCTTCGTTGCGCACGCGTTCCACGCCGAGCCTTCTTCTGGCGCGCTTTGCAAGACC  ATTGAGGCCGCTTGCAAGCTCCGCTACCAGAAGTGTTTGGACGCTCACGGCGGCGCGGCG  TCGCTGGCCAGCGGCGGCAGCGGCGGCGGCAGCAGCGGCGGCAGCGACGCCAGCCGCGCG  TCGCTCGGGCAAGCGCTCAAGTCGCTCGTGGGCTCGCTCACCGGCCGCAGGGGCTCG |
| 43038 | ATGGATGTACGTGTTGTGACTGGAGTAGCGGTATTCTTTATATCATGCGTGAGTACCGAG  CTACATCTGAGCGAGTGTGAAGTGGCTGCAAATATCGAGTCGGGCTACGTCTGCGGGAAG  CTGAGGCCTGACGCTGGCGGCAATGAATATGCAAGCTTCCGAGGGATTCCGTACGCCAAA  CAGCCGTTGGGAGACCTCAGGTTTAAGGAGCTAGAACCTTTAGAGCCTTTCACGGAAGTA  TTTGATGCCAGAGAAAGTGGCCCCGTTTGTCCCCAAACAGATGTATTTTACGGAGAACTT  ATGCAGCCTCGGTCTATGGGCGAAGACTGTATACGGATTAATGTACATGTACCCTCTAAA  GTGGTTCCGGATCTTGACAATTCTAGTAACAGTAAACCGCTTCTTCCGATCTTGTTCTTT  ATTCATGGAGGATCATTCGCATTTGGTTCTGGAGACGCCGATGTTTACGGGCCCGAGTAC  CTCGTCACTAAAGACATTATAGTTGTCACATTTAACTATAGGATAAATGTTCTGGGATTC  CTCTCCCTTGACACAAATGACATTCCAGGCAACAATGGCATGAGAGATGCCATCACAGCG  CTGCGTTGGCTCCAAAGGAATGCCAAATCGTTCGGCGGAGACCCAGATGAAGTTACCCTG  GCAGGTCATAGCTCCGGAGCGGTCATGGCGCATCTCATATCTATCTCTCCTGCCGTCGAA  TCTGGAGAACTTTTTAAGAGGGTCATCGCTCTGAGCGGAAACGCGCTTTCAAACTTTTAC  AACAGCTCGCCACAATCATCAGCATTTATGAATGCCGCTTTTTTCACTTTACTAGGAATA  TCACTATTAGAATCCACAGAAGTCATTTACAAAAATCTTGTGGCCGCAAATATAGAGGAC  CTGTTAGAAGCCAACAAATCTATACTGAACCTTTCAGGCTTGCAAGCTTTCGGCCCCGTC  GTAGAATCGACGAAAGATAACATCACAAGTATACTACCAAAGTATGCTGAAGATCTTGTA  AAGGAAGGAAAAGGCAAAGAATATCCCCTATTACTAGGTTTTACTAATAACGAATTTGAG  GCGTTTACGGTGAGGTACAAAGCGATAAATGTTGAATTGAAGATGCTTACCGAACCAGAT  GTGGTCACGCCGTTAATACCGGGGTGTGTAGCGCCTTTTCTGAATCCGTTGACCTCTCCG  ATAGTTTCTTTGAAAATATTGGCCCAATATTTCAATAGTTCTATAATGACTTTTCCGAAG  TACATACCTTATGCGAATGACAAATTTTATGTGTACCCAGTCATAAGGTTAAGCCAGAGA  AGGGTGGCTGCTGGTGCAGCACCAACGTATGTGTACCAATTTGCATACAGTAACGAAGAT  AGTGTCTTGAAGCGAGTGCAAAAGAGAGATTTCAAAGGAGCTGGCCATTTTGAGGACATC  ACTTACTTCTTTAGAGCAAATTCTGTACTAGGACCAGTGACAGAAGAAGACCAAACAAGT  TCCAACTCAAAAATGAGGGAAAAGATGACAACAGTTATTACTAACTTTGTGAAATCTGGG  AAACCAGGTCCAGATGTAGCTTTGTTTTTAGGATTTATTCCCTTATGGACGGCTACCTCA  TGGCCAGCAACCACTACGACTATGATGATTCAACAAATCACTGATCCCGTAGACTTTAAA  AACCGGATAGCCACTAACCAAGAGATAGAAATAAAAAAATTCTTTGATAACATCTACTGG  ATTGCTGGATTGGGAAAG |
| 43754 | ATGGTGATGGAGAGCGTGCAGGTGGAGCTGGACCCCAGCGAGCTGCTGCTGCCGGACCAG  GACTTCGACATGGAGACCGTCGAGATCGAGGACACGCAGCCGGAAAAAGAGGATCCAATC  AGCCTCCTCACATCATCGGATGAAGATGACGTCATAATTGAAGAGCCACACATTGACACC  GTGGAGGTGTCCGATGAGACGGACGAGGACGATATGCCCCTCGTCAAACTCCTCAAGAGA  AGACGAGCGGACAAGAAGAAGAACCTCGCCAGCCTGACTCAGGCCTCCATCGCTGCCAAC  GGGGAGACAGATTTAGCCAAGATTCTCTGGGGGATGTATGAGTATTACTGCGTTCAGTGT  CATTTTACAACGTCTAGTGGAACGGAGTATAGACGCCATACCGCGAGCCACGCGAAAGTG  TTAATGATGTGCCAAATTTGCGGTTACATGACCGCTAGTGAGAGTCAGTTTACGAAGCAC  GAGAAACAACACGGTGAAAAGAAGTATAAGTGTCATTTGTGTACGTACAAAGCGAAGCAT  AATATGAGTTTGCTGTACCATCTAAAAGGGCACAAAGCCGAGGACTTTATCAAGTCGGCT  ACAGTGTCCACGTTGAAAAGGCTGAAGGCAACGGGACGGGTGTTTGAGTGTAAGTCTTGT  TCGTATACAACTAAGAAGCGGTGTGATTTGAAAAGGCACATCACTCGTAGGCATAAGGAG  GACAATGATGAGGATTTTATACCT |
| 44378 | ATGGATCCCGCGTCGGCTACTGTAGCCTCCGTGCAGGGACTCCAGGGCGCCTTGCATGTC  CTCACGGCTCTTCAGCTCACTTCATACCGGTTTCCCGAACCGAAAGTTCTTCAAGATGGA  GCCACCTTTGACTACATAGTAGTCGGTGGAGGAACAGCTGGCTGTATCGTAGCAAGTCGA  CTATCTGAAAACCCCAACTACACTGTGGCGCTTATAGAAGCTGGTGGTATACCGCCTCTG  GAATCCGTGCTTCCACCATTCTACCCGCTGCTTACAAATACTTCTTATGACTACGGCTTT  CAAACAGAAAGGGACCAATTCAGTCAACAATACATACAAGGAGAAGCCGTTACCATGTTT  GCTGGCAAAATGTTGGGAGGAAGCAGTTCGTTACAAGCTCTCCTTCACTTCAAAGGAAAC  CCTCGTGAGTACCAGCGTTGGGCAGACGCCGCCAACGACAGTTCGTGGAACTACGACGGA  TTACTGCCGTATTTCATCAAAAGCGAACGACTTGAAGACCCAGAACTCCTCAATTCTCCT  AGCGTTAAAAACTACGGTACCTCCGGCAAAATAGCTCTGACTCGGGAACCAAATGACGCT  AACACAGGAATCTTTGAATCCTTTCAAGAACTTGGATATGAACACCTTCCAGATCAAAAT  GGTAGAAATACTTTAGGCATAGCTGATGGTCTCTTTGACATATCGTTAGGCGATGGTACA  GATGGCGTTCGCCAAAGCACTGCTGAATCCTACTTGCGTCTGGTAAAGGATCGTAGTAAT  TTATTCGTATTTGACGAAACAACCGTTACTAAGATTCTTTTTGATGGCAATAAGAATGCT  GTTGGAGTAGAAGCTTTGACCTCAAATAAAAAAGTTGTTAAAGTAAATGCTCTAAAAGAA  GTAGTGCTTGCAGCAGGAGTGTTCAAAACCCCACAGTTACTACTCTTATCTGGAGTTGGT  CCTAAGAGATATCTACAAAACTTCGGCATTCCCCTTATTTCTGACTTGCCTGTAGGCGAG  ACTTTGCAGGACCACGCATGCACGGTCCTGTTTTACAAATTGGAGCGGTCAAATAAAACG  CCTCCACCCGTAAATCCACATAAGTTTAATTCTCCTATAACTGTTGCAAATAAGGCTTTG  AATAAGTCAGATCCACATGACGATATACAGTCGATCAATTTGAGATTCCCTCGCGATCCC  TCATCTTCTGGAATTAATCAGTTTTGTAACATAATTATTAAGTATAAAAAAAATATTTGT  AACGAACTAGCTTTTGCTAACACTGAAAGAGATATAATGATGTTTGAAATTTACAAATTG  CAGCCTCTTTCTTATGGGAAAGTGGGATTGCGGACCACAAATCCTCTTGATGATCCATGG  ATTTATATGGGCTATTTTAACAACCTTACTGATTTAGATAATTTGGCGACGTACTTAAAA  GAGTTCTCAGCAATTGTTAACACTACTTACTTCAAAAATGTGGATGGTGAAAGGGTAGTT  TTTGATCTGAAGGAATGCGATTACTTGGAGAAAGAGTCTTGGGATTACTGGAGATGTTAT  GCGAGGGTCATGTCGGCTACTGTGTGGCATCCCTTGGGAACATGTCCAATGGGGCCTGTT  TTAGACTCGAAACTGAAGGTGCGAGGAGTTCAGAAGCTCCGTGTAGTGGACGCAGCGGGA  ATACCAAGTCATAATAGCGGGAAATTGTTCGCGGCGGTGGTGGTCTTTGCAGAAAAGGCT  TCAGATTTGATCAAAGCAGACATGAATTCGAAAACAACT |
| 44963 | ATGGACGACGCCGGCAGCAACAAGCAACGGCAGGCGACGCGAGTCTTCAAGAAGAGTTCA  CCCAATGGAAAGATAACAGTGTATTTAGGGAAGAGGGACTTCGTAGACCACATTACACAT  GTTGACCCTATTGACGGGGTAGTACTAATCGACCCCGAATATGTAAAGGAGAGGAAGGTG  TTCGGTCACGTACTGGCCGCCTTCAGGTATGGCCGCGAGGACCTGGACGTACTCGGGCTC  ACCTTCCGCAAGGACCTCTACCTGGCCGCCGAACAGATATACCCACCGACGAACACGGCG  AAGAGGCCGCTGACGCGGCTGCAGGAGCGGCTGGTGCGCAAGCTGGGCCCCGCCGCGCAC  CCCTTCTACTTCGAGCTGCCCCCGCACTGCCCCGCTTCCGTCACGCTGCAGCCCGCGCCC  GGCGACACCGGCAAGCCGTGCGGCGTCGACTACGAGCTCAAGGCCTTCGTCGCCGACTCG  CAGGATGACAAACCACACAAGAGAAATTCTGTCCGATTGGCGATCAGGAAGATAATGTAC  GCGCCGAGCAAGCAGGGGGAGCAGCCTTCCGTAGAGGTCTCCAAAGAGTTCATGATGAGC  CCCAATAAACTGTACCTGGAAGCTTCTTTAGATAAGGAGTTATACCACCATGGCGAGAAC  ATCGCAGTGAACGTGCACATAGCGAACAACTCTAACCGGTCCGTGAAGCGCATCAAGGTG  TCGGTGCGGCAGTTCGCCGACATCTGCCTCTTCTCCACCGCGCAGTACAAGTGTACCGTC  GCTGAGGCTGAGAGCGAGGAGGGCTGCCCCGTGGGCCCGGGGTTCACGCTGAGCAAGGTG  TTCACGCTGACACCACTGCTGGCCAACAACAAGGACAAGTGGGGGCTGGCGCTCGACGGA  CAGCTCAAGCATGAGGACACCAACCTCGCGTCCAGCACCTTGATCGCAGACCCATCACAG  CGTGAAAATCTAGGTATCATAGTCCAATACAAGGTCAAAGTAAAACTCTGTCTAGGCCCG  TTAGGAGGCGACCTGAGCGCGGAGCTGCCGTTCATCCTGATGCACCCCAAGCCCGAGGAG  GAGACCCCGCGCGCGCCGCCCGAGCCCGCGCCGCAGGACCACGACCTCATCCAGCTCGAC  CCGCATCCTGACGAGAACGGGCAGGAGCAGGACGACGACATAATATTCGAAGACTTCGCG  CGACTGCGGCTCAAGGGCGCCGAGTCCGACGCC |
| 46823 | ATGACATCTCCGATTGGAGCAACAAATATCAACAACAACCACCGACACGCTGTGAATGCC  GCAATGTCTGCACATCTTGGGGGCGGAAGCCAGGCTGCCAATTTTGGCATGGCCGTGGCC  GTGCCTTCTCAAGGATTGTCTGCGGGGTATGGACTACACGCGGCCAGCTGCAGTGATTCT  TCATCAGAATCCCATTCTCCTGAAATGCCATCTGCCAAAGCGAGCGATCGAGAGTCACGA  ATTATCGCTGAGAAACAACGAAGAAGTCAGTATAATTCTCACATAACTCAATTAACGGCT  TTGTTGTCGGATATAGTACATGCACCACGCAAAGTAGATAAAACAAGCGTTCTAAGGCTT  GCAGCGAATAAACTACGCAATGAGCATGTTTTCGGTGATACCATCAAATGTGGCCATATA  GAAACATGGTCAACTGCTTTTATCAAGTATTTTGATCTTTTTGGCTCAATTTTATTTGCT  GTTACTTGCCGGGGCCGAATATTTCTTTTTTCTCCCAATGTACAAGAAAAGTTAGGTTAT  TGCCATGTTGATTTACTAGGTCAAGACTTCTACAATTATATTCACAATGATGACAAGAAT  ATTCTCCGTCAACATATTTATCCTCAAGAATTGAGAAGTGGTTGTGAACAAAGACTCTTT  GAACATCATCACACTTTCCATATTCGCATTATGAGAGCTGGTGCGAAATCTGACCCTCCT  CGTTATGAACATTGCCGTATAGATGGTGTATTGAGACGGTCTGATCATGCTACAGCAAAT  GGGGTACAAGATCAACAAATAATTAGAAGGCAGCGTGTGAGAAGAATCCGCACATTTTCA  TCAAGTGGTAATGATTATGTTTTTATTGGCATGATTAAAGTTATGTCAAATGCCTTGCCA  ACGCAGATTTTACCTCCAACTGCATATTCCGAATATTGGACAAGGCATTTAGTAGATGGC  CGCATAGTGCAGTGTGATCAAAGTATCTCCCTGGCACTAGGATACATGATAGAAGAGGTG  ACAGGTACTTCAGCATTTGTGTTTATGCATAAGGATGATGTCAGATGGGTCATTTGTGTG  CTAAGGCAAATGTATGATCAGAGCAGAGAATTTGGGGAATCATATTACAGGCTTATGTCA  CGTTCAGGTCACTTTATTTATATGAGAACTCGTGGTTTTCTTGAGATTGACAAGGATTCC  AAGAAAGTCCAGAGCTTTGTATGTGTCAACAGTGTAATTGGAGAGGATTATGGACGTCGA  ATGATGGATGAAATGAAACGTAAATATTCTGTGATAGTAGACATGGCAAAACAGGAGCAT  GATAATGGAGATATTAGAGATGAAGCCCCAGTTGAGCATCCAAAACAATTGGAGAGAATT  GTGATGCATCTGGTAGAGCCTTCAGGGAGCGAGAGTGCTGATGAGCTAAAACTGGTACCT  CCCTCCAAAGAAAATATTATAACTGCTATTAAAAACAGTGAGAGAGTAGTTCAAGAAACT  GGGGTGCGGTTTGATTCACGCAAACGTAAGAACTCTGACAGTGAAAACAGTGACCAATTA  AAAAGGCACAGTGGATTACAGGATTTCAGTAGTTAT |
| 46824 | ATGACATCTCCGATTGGAGCAACAAATATCAACAACAACCACCGACACGCTGTGAATGCC  GCAATGTCTGCACATCTTGGGGGCGGAAGCCAGGCTGCCAATTTTGGCATGGCCGTGGCC  GTGCCTTCTCAAGGATTGTCTGCGGGGTATGGACTACACGCGGCCAGCTGCAGTGATTCT  TCATCAGAATCCCATTCTCCTGAAATGCCATCTGCCAAAGCGAGCGATCGAGAGTCACGA  ATTATCGCTGAGAAACAACGAAGAAGTCAGTATAATTCTCACATAACTCAATTAACGGCT  TTGTTGTCGGATATAGTACATGCACCACGCAAAGTAGATAAAACAAGCGTTCTAAGGCTT  GCAGCGAATAAACTACGCAATGAGCATGTTTTCGGTGATACCATCAAATGTGGCCATATA  GAAACATGGTCAACTGCTTTTATCAAGTATTTTGATCTTTTTGGCTCAATTTTATTTGCT  GTTACTTGCCGGGGCCGAATATTTCTTTTTTCTCCCAATGTACAAGAAAAGTTAGGTTAT  TGCCATGTTGATTTACTAGGTCAAGACTTCTACAATTATATTCACAATGATGACAAGAAT  ATTCTCCGTCAACATATTTATCCTCAAGAATTGAGAAGTGGTTGTGAACAAAGACTCTTT  GAACATCATCACACTTTCCATATTCGCATTATGAGAGCTGGTGCGAAATCTGACCCTCCT  CGTTATGAACATTGCCGTATAGATGGTGTATTGAGACGGTCTGATCATGCTACAGCAAAT  GGGGTACAAGATCAACAAATAATTAGAAGGCAGCGTGTGAGAAGAATCCGCACATTTTCA  TCAAGTGGTAATGATTATGTTTTTATTGGCATGATTAAAGTTATGTCAAATGCCTTGCCA  ACGCAGATTTTACCTCCAACTGCATATTCCGAATATTGGACAAGGCATTTAGTAGATGGC  CGCATAGTGCAGTGTGATCAAAGTATCTCCCTGGCACTAGGATACATGATAGAAGAGGTG  ACAGGTACTTCAGCATTTGTGTTTATGCATAAGGATGATGTCAGATGGGTCATTTGTGTG  CTAAGGCAAATGTATGATCAGAGCAGAGAATTTGGGGAATCATATTACAGGCTTATGTCA  CGTTCAGGTCACTTTATTTATATGAGAACTCGTGGTTTTCTTGAGATTGACAAGGATTCC  AAGAAAGTCCAGAGCTTTGTATGTGTCAACAGTGTAATTGGAGAGGATTATGGACGTCGA  ATGATGGATGAAATGAAACGTAAATATTCTGTGATAGTAGACATGGCAAAACAGGAGCAT  GATAATGGAGATATTAGAGATGAAGCCCCAGTTGAGCATCCAAAACAATTGGAGAGAATT  GTGATGCATCTGGTAGAGCCTTCAGGGAGCGAGAGTGCTGATGAGCTAAAACTGGTACCT  CCCTCCAAAGAAAATATTATAACTGCTATTAAAAACAGTGAGAGAGTAGTTCAAGAAACT  GGGGTGCGGTTTGATTCACGCAAACGTAAGAACTCTGACAGTGAAAACAGTGACCAATTA  AAAAGGCACAGTGGATTACAGGATTTCAGTAGTTAT |
| 48099 | ATGGATGTCCTGCCGAGCGGTAACCTATTCCGCGAGTTGCAGGATGTGACTGACACTGGA  TATTTCGAGTGGAAGCTGTCCCTGGAGGACTACTGGCAACAGACATGCTACGAAATGGAG  AGGTACCTCCGGGAAGAACCCAGAGGAAAGCGGCGCGAGCCGTCCCACGAGGAAGAGTGG  GCCATATCGACAGCATCCTCCCCACCAACACCGCCCCGGTCTCCTCCCTCCCCACCGCCA  AGGCTCTTCACTATCACTGCCGTGAAAAGCGAGCCGCCAAGCGACTCCGACGAACCGAGA  GATGAGCCTCCGAGCCCAGCCCCCGCCCGCCGGGCGCATGCGCCGCGCCACCACCCCCAC  CCCCATGCGCACGCGACTGACCCGCGCCGTCGCGTCCACCGTTGCGAGTTCCCGGCCTGC  GACAAAGTCTATACGAAGAGTTCGCATCTCAAAGCGCATAAGAGAACCCATACTGGAGAA  AAACCATACAAGTGTTCATGGGAAGGGTGCGAGTGGCGGTTCGCGCGTTCCGACGAACTG  ACGCGGCATTACCGCAAACACACGGGCGCGAAGCCGTTCCGGTGCCGCCACTGCGAGCGC  TGCTTCTCCCGCTCCGACCATCTCGCGCTACATGCCAAGCGCCACGCG |
| 52909 | ATGTATAAACAAGTCAAATCCATCATATCTTTAGTAAATAAACAGAAGGCTGCCCTTCTA  AAATTGCAACCTATTCCGAAGCAAATCGGAGAACAAAACGATTCTCTACTGTTTGTTACT  CCATGCCACGTGAATGCAATACGAGAAGCGAAGACCAATTTGGATGATTTAATAAATCGA  GTAGACAGTTTATTAAATCACAATCGATTTATTTTGAAACACTCATCTGTCTTCGGCGTG  GATGATACACTACATCCACTATTACGAAGCATGAGTGAGTTAAAAGACTACTTGAAAGAA  ACAGTTGAAAATATTGACGCGCTAGATAAACCTGATGATGCAACAATGGAGGAGAATTTA  ACTGACGCTGTGATCAGTCAGTCTGAGGACTTGATAGCCACGATGCTTTTGATAATCCAG  TCTGTATACAAGAAGCATTTGCCTGAAGGGAATGGTAGTAGAGAATTGTTACAAGCAATT  GATGATATTATTGAAAAAGACAAGGTTGAGAAGGAGAGTGAGGAATCGAAAGAGTTGCTT  GAAGATAAGCATTTGAAGGAACATTTGCAGGACAAATTGTCGGGAGATGCGAAGATGTTG  CAGTTGGAGTCTGTGATAGGTAAATGTGAGAAGTTGTTGGCGACATTTGTGCAAAATATT  GCTACTAATAAAGCACTAGATGGCGCGTCTGATGCAGTCACAAGGACGATCCTGGTGTTG  GAGCAAACGGTTCTGTTTGTGCAGTATTTCGTGACACAGAAGGTGGCCGTTCACAGAGTG  ACCTGTAAGATGCTTTCTGTCCTGTTGAAGATATTCTCCGATTTAGCAGCTAAAGGGTTC  TGCAGACCCTCAGATTTGGACACTGAAGAAGGCGAGGGAGAAGGTGGCCCTGGAAAGTTA  TCTGGTGGCACTGGTTTGGGAGAAGGAGAGGGACAGAAGGATGTTTCTGATAGAATAGAA  AACCAGGATCAGCTCGAAGACGCTCATCGACCAGGTGAAGAGAAGAAAGAAGAAGAAAGG  GATTGTAAAGAAGAAGAGAAAGGCGTAAACATGACTGATGATTTTGACTCGCATCTACAA  GACGTTGAGAAGAAAGAAGGAGATGAGTCAGAACAAGAGGATGATGAAGATGACGCCGAT  AAACAAATGGGCGACACCGACAATGCTGCTGAAAAACTTGACCAGCAAATATGGGGATCC  GAAGATGAAGACGTCGATGATAACCAAGAGAAAAAAGACAAGGAAGAGAAAGGACAAGGC  GAGAGCACGGGCGAGAAAGAGATGGGTGCTAAGGAAGAAGAGCAAGGCGCTGATGACGGC  TCGGAAGGGAAGGAACGGAAAGAGAAAAAGGATATCAATGAGATGAAGGAGCCCGAGATA  GATGACGATCATGTCGACCCGTACCACGGCAACCACCCGCAGCTGCCTGAACCGGAAGAT  TTTGAACTACCTGACAATATGGATGTGGGTAGCGAAGACGAAGATAAGGAGGGTGAAACA  GAGACGGAGAATCCGTTTGAAATAGACGTGACAAAGGACGATATCCCGAAAGAGCAGGAA  GAAGTCGAACAGGACGATAAGGGGGAGGAGGGAAAAGACACGAAGCATGGCACTGAAGTG  TCCAGCGATGAAGAGGGCGAAGGGGATGAGGAGAATAAGGAACAGCAGGATAAAGAAGAC  GACAGTCAGCCAGAAGAAACTGGACCCGATTCAGAATCTCAAGAAGAAGACAAGGGTGAC  TTAGATGAGAACGCTAACGCTGATCCTCAAAATCCAGAAGAAGCAGAACAGCCAGAAGAA  ACCGAGACGGAAAAAGAAGACTTACCAAATAATCCAGAGAAGATGGACGTAGATGATGAA  GTGGAACAGAAGAATCACGAAGCCAATCCTCAGGCGAATCCGTCGAATGACGATCAGTCT  GCGGAGGATAGAGCAGAGAACGCGCAAATGGATAGAGGAACAGATGATAACGTTGAGACG  AATGCAGAACAGAAGAAAGACGAGGAAACTGCGCCTTATGAGCAAGCTCAAGAACAAGTA  GGCGAGGAAAATCAGTCGATGGGACGATCGGAGCTGGACAAAAGTGAGAAGGGACATAGG  GGGGAGAAGCAAGCCGCTAGCCGGGCGGAACGCGCCCAGGAGAAGCGGGATAGACATGAG  AACAAGCCTGGAAAGACTGACCAAGAACGCACTTTGGGCGATGTAACCGACAAGAAACAC  AAGCAATCGCAGACACTGAATGTGGAGCGCGAGGAAGAGACTGAAGAGGGCGCGCGTGGC  GCAGATGAAGAGGAGAAGGATGCTGACGCCTACCAACACGTCAAGCAAGCTAATAAGGAC  GACTTACAGGCTATAGACGCGGCTACCAAAGAGCAAGCGGAGCAGCAGCCCACTTTGCAG  CAAGAAGACGAGGAGGCTGACAAGCCGAAGGAAGATGAGGAGATTGCGATGGACGTAGAC  GAAGAAGAGTTGCAGGTGGAAAAGTCGGAAGAATTAAAGCCAGAAAAAGTGAAAGAGGGC  GCGGACAAAGAGAAAGACAAGTCTGGCACGAAGAAGGACGGTGATGAGGAGGCGACTGGG  GAGACCGGCATTGAAGTGGAGGGCGAGAAGGTGGTCACACAGCATGTGCCACGCGGAACT  GACACTACATTCCATACAAGACTCGAAGAACCACAAGCAGAGCGATCCGAAGATATGTCT  ATGGAGCAATATATGAGCATTCGTGAGTGGCTGCGCAGCGGGCCTGCGCAGGCGCAAGGC  TCAGCCAACGCGTGGCGCTCGCTGTGGCAGGAGTGCGGCCCGGCGGCGCGGGCGCTGTGC  GAGCGGCTGCGGCTGGTGCTGGAGCCCACGGGCCGCTCCAGGAGAGCAGGTGACTTCCGG  ACGGGGCGCGCCATCAACATGCGGCGCGTGATCCCGTACATCGCGTCGCACTTCCGCAAG  GACCGCATCTGGCTGCGCCGCACCAAGCCGGCCAAGCGCGAGTACAAGATCGCCATCGCC  GTCGACGACTCTAGCTCCATGAGCGACAACAGAAGCAAGGAGTTGGCTTTCGAGAGCCTC  GCGCTCGTCTCACAGGCTTTGAACTTGCTCGAATCTGGCGATCTCGCCGTCCTCAGCTTC  GGCGAGCAACCGAACCTGCTCCACCCATTCACTGAGCAGTTCTCCGAACACTCCGGCTCC  AAGATCCTTGAGCAGCTCCGCTTCGAGCAGACGAAGACCAAAATAGCTCAGCTGCTCGAC  TTCGTAACGGTCATGTTTGAGGAGCAGTCAGTCAGGAGTGATGCTGTGAACGCCAAGTTG  CTGGTGGTGGTCAGCGATGGGAGAGGAATCTTCTCGGAGGGAGAGACCCGTGTGGTGCAA  GCGGTGAGGAGAGCGCGGCAGCAGGGGATATTCCTCGTTTACGTTATAATCGACAATCCG  GATAATAAGGACTCCATAATGGACATCAGGCGACCACTCCTAGACCCAGTGAACTTCAGC  CTGTCCGGATTCGTTCCATACCTGGATACGTTCCCGTTCCCCTTCTATCTGATCCTTCGG  GATATGTCGGCGTTGCCCACTGTATTAGGAGATGCATTACGTCAGTGGTTCGAGTTGGCT  GCTAATGTTGCCAGT |
| 52910 | ATGTATAAACAAGTCAAATCCATCATATCTTTAGTAAATAAACAGAAGGCTGCCCTTCTA  AAATTGCAACCTATTCCGAAGCAAATCGGAGAACAAAACGATTCTCTACTGTTTGTTACT  CCATGCCACGTGAATGCAATACGAGAAGCGAAGACCAATTTGGATGATTTAATAAATCGA  GTAGACAGTTTATTAAATCACAATCGATTTATTTTGAAACACTCATCTGTCTTCGGCGTG  GATGATACACTACATCCACTATTACGAAGCATGAGTGAGTTAAAAGACTACTTGAAAGAA  ACAGTTGAAAATATTGACGCGCTAGATAAACCTGATGATGCAACAATGGAGGAGAATTTA  ACTGACGCTGTGATCAGTCAGTCTGAGGACTTGATAGCCACGATGCTTTTGATAATCCAG  TCTGTATACAAGAAGCATTTGCCTGAAGGGAATGGTAGTAGAGAATTGTTACAAGCAATT  GATGATATTATTGAAAAAGACAAGGTTGAGAAGGAGAGTGAGGAATCGAAAGAGTTGCTT  GAAGATAAGCATTTGAAGGAACATTTGCAGGACAAATTGTCGGGAGATGCGAAGATGTTG  CAGTTGGAGTCTGTGATAGGTAAATGTGAGAAGTTGTTGGCGACATTTGTGCAAAATATT  GCTACTAATAAAGCACTAGATGGCGCGTCTGATGCAGTCACAAGGACGATCCTGGTGTTG  GAGCAAACGGTTCTGTTTGTGCAGTATTTCGTGACACAGAAGGTGGCCGTTCACAGAGTG  ACCTGTAAGATGCTTTCTGTCCTGTTGAAGATATTCTCCGATTTAGCAGCTAAAGGGTTC  TGCAGACCCTCAGATTTGGACACTGAAGAAGGCGAGGGAGAAGGTGGCCCTGGAAAGTTA  TCTGGTGGCACTGGTTTGGGAGAAGGAGAGGGACAGAAGGATGTTTCTGATAGAATAGAA  AACCAGGATCAGCTCGAAGACGCTCATCGACCAGGTGAAGAGAAGAAAGAAGAAGAAAGG  GATTGTAAAGAAGAAGAGAAAGGCGTAAACATGACTGATGATTTTGACTCGCATCTACAA  GACGTTGAGAAGAAAGAAGGAGATGAGTCAGAACAAGAGGATGATGAAGATGACGCCGAT  AAACAAATGGGCGACACCGACAATGCTGCTGAAAAACTTGACCAGCAAATATGGGGATCC  GAAGATGAAGACGTCGATGATAACCAAGAGAAAAAAGACAAGGAAGAGAAAGGACAAGGC  GAGAGCACGGGCGAGAAAGAGATGGGTGCTAAGGAAGAAGAGCAAGGCGCTGATGACGGC  TCGGAAGGGAAGGAACGGAAAGAGAAAAAGGATATCAATGAGATGAAGGAGCCCGAGATA  GATGACGATCATGTCGACCCGTACCACGGCAACCACCCGCAGCTGCCTGAACCGGAAGAT  TTTGAACTACCTGACAATATGGATGTGGGTAGCGAAGACGAAGATAAGGAGGGTGAAACA  GAGACGGAGAATCCGTTTGAAATAGACGTGACAAAGGACGATATCCCGAAAGAGCAGGAA  GAAGTCGAACAGGACGATAAGGGGGAGGAGGGAAAAGACACGAAGCATGGCACTGAAGTG  TCCAGCGATGAAGAGGGCGAAGGGGATGAGGAGAATAAGGAACAGCAGGATAAAGAAGAC  GACAGTCAGCCAGAAGAAACTGGACCCGATTCAGAATCTCAAGAAGAAGACAAGGGTGAC  TTAGATGAGAACGCTAACGCTGATCCTCAAAATCCAGAAGAAGCAGAACAGCCAGAAGAA  ACCGAGACGGAAAAAGAAGACTTACCAAATAATCCAGAGAAGATGGACGTAGATGATGAA  GTGGAACAGAAGAATCACGAAGCCAATCCTCAGGCGAATCCGTCGAATGACGATCAGTCT  GCGGAGGATAGAGCAGAGAACGCGCAAATGGATAGAGGAACAGATGATAACGTTGAGACG  AATGCAGAACAGAAGAAAGACGAGGAAACTGCGCCTTATGAGCAAGCTCAAGAACAAGTA  GGCGAGGAAAATCAGTCGATGGGACGATCGGAGCTGGACAAAAGTGAGAAGGGACATAGG  GGGGAGAAGCAAGCCGCTAGCCGGGCGGAACGCGCCCAGGAGAAGCGGGATAGACATGAG  AACAAGCCTGGAAAGACTGACCAAGAACGCACTTTGGGCGATGTAACCGACAAGAAACAC  AAGCAATCGCAGACACTGAATGTGGAGCGCGAGGAAGAGACTGAAGAGGGCGCGCGTGGC  GCAGATGAAGAGGAGAAGGATGCTGACGCCTACCAACACGTCAAGCAAGCTAATAAGGAC  GACTTACAGGCTATAGACGCGGCTACCAAAGAGCAAGCGGAGCAGCAGCCCACTTTGCAG  CAAGAAGACGAGGAGGCTGACAAGCCGAAGGAAGATGAGGAGATTGCGATGGACGTAGAC  GAAGAAGAGTTGCAGGTGGAAAAGTCGGAAGAATTAAAGCCAGAAAAAGTGAAAGAGGGC  GCGGACAAAGAGAAAGACAAGTCTGGCACGAAGAAGGACGGTGATGAGGAGGCGACTGGG  GAGACCGGCATTGAAGTGGAGGGCGAGAAGGTGGTCACACAGCATGTGCCACGCGGAACT  GACACTACATTCCATACAAGACTCGAAGAACCACAAGCAGAGCGATCCGAAGATATGTCT  ATGGAGCAATATATGAGCATTCGTGAGTGGCTGCGCAGCGGGCCTGCGCAGGCGCAAGGC  TCAGCCAACGCGTGGCGCTCGCTGTGGCAGGAGTGCGGCCCGGCGGCGCGGGCGCTGTGC  GAGCGGCTGCGGCTGGTGCTGGAGCCCACGGGCCGCTCCAGGAGAGCAGGTGACTTCCGG  ACGGGGCGCGCCATCAACATGCGGCGCGTGATCCCGTACATCGCGTCGCACTTCCGCAAG  GACCGCATCTGGCTGCGCCGCACCAAGCCGGCCAAGCGCGAGTACAAGATCGCCATCGCC  GTCGACGACTCTAGCTCCATGAGCGACAACAGAAGCAAGGAGTTGGCTTTCGAGAGCCTC  GCGCTCGTCTCACAGGCTTTGAACTTGCTCGAATCTGGCGATCTCGCCGTCCTCAGCTTC  GGCGAGCAACCGAACCTGCTCCACCCATTCACTGAGCAGTTCTCCGAACACTCCGGCTCC  AAGATCCTTGAGCAGCTCCGCTTCGAGCAGACGAAGACCAAAATAGCTCAGCTGCTCGAC  TTCGTAACGGTCATGTTTGAGGAGCAGTCAGTCAGGAGTGATGCTGTGAACGCCAAGTTG  CTGGTGGTGGTCAGCGATGGGAGAGGAATCTTCTCGGAGGGAGAGACCCGTGTGGTGCAA  GCGGTGAGGAGAGCGCGGCAGCAGGGGATATTCCTCGTTTACGTTATAATCGACAATCCG  GATAATAAGGACTCCATAATGGACATCAGGCGACCACTCCTAGACCCAGTGAACTTCAGC  CTGTCCGGATTCGTTCCATACCTGGATACGTTCCCGTTCCCCTTCTATCTGATCCTTCGG  GATATGTCGGCGTTGCCCACTGTATTAGGAGATGCATTACGTCAGTGGTTCGAGTTGGCT  GCCAATGTGGCCAGT |
| 52911 | ATGCTTTCTGTCCTGTTGAAGATATTCTCCGATTTAGCAGCTAAAGGGTTCTGCAGACCC  TCAGATTTGGACACTGAAGAAGGCGAGGGAGAAGGTGGCCCTGGAAAGTTATCTGGTGGC  ACTGGTTTGGGAGAAGGAGAGGGACAGAAGGATGTTTCTGATAGAATAGAAAACCAGGAT  CAGCTCGAAGACGCTCATCGACCAGGTGAAGAGAAGAAAGAAGAAGAAAGGGATTGTAAA  GAAGAAGAGAAAGGCGTAAACATGACTGATGATTTTGACTCGCATCTACAAGACGTTGAG  AAGAAAGAAGGAGATGAGTCAGAACAAGAGGATGATGAAGATGACGCCGATAAACAAATG  GGCGACACCGACAATGCTGCTGAAAAACTTGACCAGCAAATATGGGGATCCGAAGATGAA  GACGTCGATGATAACCAAGAGAAAAAAGACAAGGAAGAGAAAGGACAAGGCGAGAGCACG  GGCGAGAAAGAGATGGGTGCTAAGGAAGAAGAGCAAGGCGCTGATGACGGCTCGGAAGGG  AAGGAACGGAAAGAGAAAAAGGATATCAATGAGATGAAGGAGCCCGAGATAGATGACGAT  CATGTCGACCCGTACCACGGCAACCACCCGCAGCTGCCTGAACCGGAAGATTTTGAACTA  CCTGACAATATGGATGTGGGTAGCGAAGACGAAGATAAGGAGGGTGAAACAGAGACGGAG  AATCCGTTTGAAATAGACGTGACAAAGGACGATATCCCGAAAGAGCAGGAAGAAGTCGAA  CAGGACGATAAGGGGGAGGAGGGAAAAGACACGAAGCATGGCACTGAAGTGTCCAGCGAT  GAAGAGGGCGAAGGGGATGAGGAGAATAAGGAACAGCAGGATAAAGAAGACGACAGTCAG  CCAGAAGAAACTGGACCCGATTCAGAATCTCAAGAAGAAGACAAGGGTGACTTAGATGAG  AACGCTAACGCTGATCCTCAAAATCCAGAAGAAGCAGAACAGCCAGAAGAAACCGAGACG  GAAAAAGAAGACTTACCAAATAATCCAGAGAAGATGGACGTAGATGATGAAGTGGAACAG  AAGAATCACGAAGCCAATCCTCAGGCGAATCCGTCGAATGACGATCAGTCTGCGGAGGAT  AGAGCAGAGAACGCGCAAATGGATAGAGGAACAGATGATAACGTTGAGACGAATGCAGAA  CAGAAGAAAGACGAGGAAACTGCGCCTTATGAGCAAGCTCAAGAACAAGTAGGCGAGGAA  AATCAGTCGATGGGACGATCGGAGCTGGACAAAAGTGAGAAGGGACATAGGGGGGAGAAG  CAAGCCGCTAGCCGGGCGGAACGCGCCCAGGAGAAGCGGGATAGACATGAGAACAAGCCT  GGAAAGACTGACCAAGAACGCACTTTGGGCGATGTAACCGACAAGAAACACAAGCAATCG  CAGACACTGAATGTGGAGCGCGAGGAAGAGACTGAAGAGGGCGCGCGTGGCGCAGATGAA  GAGGAGAAGGATGCTGACGCCTACCAACACGTCAAGCAAGCTAATAAGGACGACTTACAG  GCTATAGACGCGGCTACCAAAGAGCAAGCGGAGCAGCAGCCCACTTTGCAGCAAGAAGAC  GAGGAGGCTGACAAGCCGAAGGAAGATGAGGAGATTGCGATGGACGTAGACGAAGAAGAG  TTGCAGGTGGAAAAGTCGGAAGAATTAAAGCCAGAAAAAGTGAAAGAGGGCGCGGACAAA  GAGAAAGACAAGTCTGGCACGAAGAAGGACGGTGATGAGGAGGCGACTGGGGAGACCGGC  ATTGAAGTGGAGGGCGAGAAGGTGGTCACACAGCATGTGCCACGCGGAACTGACACTACA  TTCCATACAAGACTCGAAGAACCACAAGCAGAGCGATCCGAAGATATGTCTATGGAGCAA  TATATGAGCATTCGTGAGTGGCTGCGCAGCGGGCCTGCGCAGGCGCAAGGCTCAGCCAAC  GCGTGGCGCTCGCTGTGGCAGGAGTGCGGCCCGGCGGCGCGGGCGCTGTGCGAGCGGCTG  CGGCTGGTGCTGGAGCCCACGGGCCGCTCCAGGAGAGCAGGTGACTTCCGGACGGGGCGC  GCCATCAACATGCGGCGCGTGATCCCGTACATCGCGTCGCACTTCCGCAAGGACCGCATC  TGGCTGCGCCGCACCAAGCCGGCCAAGCGCGAGTACAAGATCGCCATCGCCGTCGACGAC  TCTAGCTCCATGAGCGACAACAGAAGCAAGGAGTTGGCTTTCGAGAGCCTCGCGCTCGTC  TCACAGGCTTTGAACTTGCTCGAATCTGGCGATCTCGCCGTCCTCAGCTTCGGCGAGCAA  CCGAACCTGCTCCACCCATTCACTGAGCAGTTCTCCGAACACTCCGGCTCCAAGATCCTT  GAGCAGCTCCGCTTCGAGCAGACGAAGACCAAAATAGCTCAGCTGCTCGACTTCGTAACG  GTCATGTTTGAGGAGCAGTCAGTCAGGAGTGATGCTGTGAACGCCAAGTTGCTGGTGGTG  GTCAGCGATGGGAGAGGAATCTTCTCGGAGGGAGAGACCCGTGTGGTGCAAGCGGTGAGG  AGAGCGCGGCAGCAGGGGATATTCCTCGTTTACGTTATAATCGACAATCCGGATAATAAG  GACTCCATAATGGACATCAGGCGACCACTCCTAGACCCAGTGAACTTCAGCCTGTCCGGA  TTCGTTCCATACCTGGATACGTTCCCGTTCCCCTTCTATCTGATCCTTCGGGATATGTCG  GCGTTGCCCACTGTATTAGGAGATGCATTACGTCAGTGGTTCGAGTTGGCTGCTAATGTT  GCCAGT |
| 52912 | ATGCTTTCTGTCCTGTTGAAGATATTCTCCGATTTAGCAGCTAAAGGGTTCTGCAGACCC  TCAGATTTGGACACTGAAGAAGGCGAGGGAGAAGGTGGCCCTGGAAAGTTATCTGGTGGC  ACTGGTTTGGGAGAAGGAGAGGGACAGAAGGATGTTTCTGATAGAATAGAAAACCAGGAT  CAGCTCGAAGACGCTCATCGACCAGGTGAAGAGAAGAAAGAAGAAGAAAGGGATTGTAAA  GAAGAAGAGAAAGGCGTAAACATGACTGATGATTTTGACTCGCATCTACAAGACGTTGAG  AAGAAAGAAGGAGATGAGTCAGAACAAGAGGATGATGAAGATGACGCCGATAAACAAATG  GGCGACACCGACAATGCTGCTGAAAAACTTGACCAGCAAATATGGGGATCCGAAGATGAA  GACGTCGATGATAACCAAGAGAAAAAAGACAAGGAAGAGAAAGGACAAGGCGAGAGCACG  GGCGAGAAAGAGATGGGTGCTAAGGAAGAAGAGCAAGGCGCTGATGACGGCTCGGAAGGG  AAGGAACGGAAAGAGAAAAAGGATATCAATGAGATGAAGGAGCCCGAGATAGATGACGAT  CATGTCGACCCGTACCACGGCAACCACCCGCAGCTGCCTGAACCGGAAGATTTTGAACTA  CCTGACAATATGGATGTGGGTAGCGAAGACGAAGATAAGGAGGGTGAAACAGAGACGGAG  AATCCGTTTGAAATAGACGTGACAAAGGACGATATCCCGAAAGAGCAGGAAGAAGTCGAA  CAGGACGATAAGGGGGAGGAGGGAAAAGACACGAAGCATGGCACTGAAGTGTCCAGCGAT  GAAGAGGGCGAAGGGGATGAGGAGAATAAGGAACAGCAGGATAAAGAAGACGACAGTCAG  CCAGAAGAAACTGGACCCGATTCAGAATCTCAAGAAGAAGACAAGGGTGACTTAGATGAG  AACGCTAACGCTGATCCTCAAAATCCAGAAGAAGCAGAACAGCCAGAAGAAACCGAGACG  GAAAAAGAAGACTTACCAAATAATCCAGAGAAGATGGACGTAGATGATGAAGTGGAACAG  AAGAATCACGAAGCCAATCCTCAGGCGAATCCGTCGAATGACGATCAGTCTGCGGAGGAT  AGAGCAGAGAACGCGCAAATGGATAGAGGAACAGATGATAACGTTGAGACGAATGCAGAA  CAGAAGAAAGACGAGGAAACTGCGCCTTATGAGCAAGCTCAAGAACAAGTAGGCGAGGAA  AATCAGTCGATGGGACGATCGGAGCTGGACAAAAGTGAGAAGGGACATAGGGGGGAGAAG  CAAGCCGCTAGCCGGGCGGAACGCGCCCAGGAGAAGCGGGATAGACATGAGAACAAGCCT  GGAAAGACTGACCAAGAACGCACTTTGGGCGATGTAACCGACAAGAAACACAAGCAATCG  CAGACACTGAATGTGGAGCGCGAGGAAGAGACTGAAGAGGGCGCGCGTGGCGCAGATGAA  GAGGAGAAGGATGCTGACGCCTACCAACACGTCAAGCAAGCTAATAAGGACGACTTACAG  GCTATAGACGCGGCTACCAAAGAGCAAGCGGAGCAGCAGCCCACTTTGCAGCAAGAAGAC  GAGGAGGCTGACAAGCCGAAGGAAGATGAGGAGATTGCGATGGACGTAGACGAAGAAGAG  TTGCAGGTGGAAAAGTCGGAAGAATTAAAGCCAGAAAAAGTGAAAGAGGGCGCGGACAAA  GAGAAAGACAAGTCTGGCACGAAGAAGGACGGTGATGAGGAGGCGACTGGGGAGACCGGC  ATTGAAGTGGAGGGCGAGAAGGTGGTCACACAGCATGTGCCACGCGGAACTGACACTACA  TTCCATACAAGACTCGAAGAACCACAAGCAGAGCGATCCGAAGATATGTCTATGGAGCAA  TATATGAGCATTCGTGAGTGGCTGCGCAGCGGGCCTGCGCAGGCGCAAGGCTCAGCCAAC  GCGTGGCGCTCGCTGTGGCAGGAGTGCGGCCCGGCGGCGCGGGCGCTGTGCGAGCGGCTG  CGGCTGGTGCTGGAGCCCACGGGCCGCTCCAGGAGAGCAGGTGACTTCCGGACGGGGCGC  GCCATCAACATGCGGCGCGTGATCCCGTACATCGCGTCGCACTTCCGCAAGGACCGCATC  TGGCTGCGCCGCACCAAGCCGGCCAAGCGCGAGTACAAGATCGCCATCGCCGTCGACGAC  TCTAGCTCCATGAGCGACAACAGAAGCAAGGAGTTGGCTTTCGAGAGCCTCGCGCTCGTC  TCACAGGCTTTGAACTTGCTCGAATCTGGCGATCTCGCCGTCCTCAGCTTCGGCGAGCAA  CCGAACCTGCTCCACCCATTCACTGAGCAGTTCTCCGAACACTCCGGCTCCAAGATCCTT  GAGCAGCTCCGCTTCGAGCAGACGAAGACCAAAATAGCTCAGCTGCTCGACTTCGTAACG  GTCATGTTTGAGGAGCAGTCAGTCAGGAGTGATGCTGTGAACGCCAAGTTGCTGGTGGTG  GTCAGCGATGGGAGAGGAATCTTCTCGGAGGGAGAGACCCGTGTGGTGCAAGCGGTGAGG  AGAGCGCGGCAGCAGGGGATATTCCTCGTTTACGTTATAATCGACAATCCGGATAATAAG  GACTCCATAATGGACATCAGGCGACCACTCCTAGACCCAGTGAACTTCAGCCTGTCCGGA  TTCGTTCCATACCTGGATACGTTCCCGTTCCCCTTCTATCTGATCCTTCGGGATATGTCG  GCGTTGCCCACTGTATTAGGAGATGCATTACGTCAGTGGTTCGAGTTGGCTGCCAATGTG  GCCAGT |
|  |  |
